# Alginate foraging is conserved in geographically and taxonomically distinct ruminant microbiomes

**DOI:** 10.1038/s41467-026-72045-z

**Published:** 2026-07-16

**Authors:** Jeffrey P. Tingley, Alessandra Ferrillo, Marissa L. King, Alemayehu Kidane, Barinder Bajwa, Xiaohui Xing, Tina Johannessen, Alexsander Lysberg, Liv Torunn Mydland, Margareth Øverland, Greta Reintjes, Anna Y. Shearer, Leeann Klassen, Kristin E. Low, Trushar R. Patel, Stephanie A. Terry, Phillip B. Pope, D. Wade Abbott, Live H. Hagen

**Affiliations:** 1 Lethbridge Research and Development Centre, Agriculture and Agri-Food Canada, Lethbridge, AB Canada; 2 Department of Chemistry and Biochemistry, University of Lethbridge, Lethbridge, AB Canada; 3 Faculty of Chemistry, Biotechnology and Food Science, Norwegian University of Life Sciences, Ås, Norway; 4 Faculty of Biosciences, Norwegian University of Life Sciences, Ås, Norway; 5 Microbial-Carbohydrate Interactions Group, Faculty of Biology/Chemistry, University of Bremen, Bremen, Germany; 6 Alberta RNA Research and Training Institute and Department of Chemistry and Biochemistry, Lethbridge, AB Canada; 7 Department of Microbiology, Immunology and Infectious Disease, Cumming School of Medicine, University of Calgary, Calgary, AB Canada; 8 Li Ka Shing Institute of Virology and Discovery Lab, University of Alberta, Edmonton, AB Canada; 9 Centre for Microbiome Research, School of Biomedical Sciences, Queensland University of Technology (QUT), Translational Research Institute, Woolloongabba, QLD Australia

**Keywords:** Microbiome, Glycobiology

## Abstract

Seaweed plays a crucial role in carbon cycling and is expected to be a valuable resource for sustainable biomass, with applications in biofuel production, human nutrition, and animal feed. Although seaweed has historically been used as a feed source for livestock grazing near coastlines, the process by which it is digested in the rumen remains unknown. Here, we show how the brown alga *Saccharina latissima* is catabolized within the rumen ecosystem of two different ruminant species using in vivo and in vitro experimental systems. Evidence of digestion was obtained using a combination of animal models, bacterial imaging, multilayered meta-omics, and enzyme biochemistry. Our results demonstrate that geographically distinct ruminants harbor conserved alginate utilization loci, of which essential enzymes were expressed in response to *S. latissima* in the diet. While core enzymes involved in alginate metabolism have been maintained throughout populations, ancillary enzymes appear to be gained or lost through gene duplication or loss events. The conservation of these systems indicates that the ruminant microbiome retains a latent capacity to metabolize marine polysaccharides.

## Abstract (web summary)

Here, the authors reveal conserved enzymes in geographically distinct ruminant microbiomes that break down brown seaweed alginate, showcasing the rumen’s remarkable capacity to adapt to novel dietary polysaccharides.

## Introduction

The effects of climate change, such as extreme drought and flooding, are deleterious to food production and reduce access to cultivable land. Thus, seeking solutions from the ocean to address the challenges associated with the harvest of terrestrial food and feed is garnering interest. Cultivated and wild-harvested macroalgae are emerging as a promising source of biomass for future application in biofuel production, human nutrition, and animal feed^1^. Certain varieties of marine macroalgae, such as kelp, grow faster than terrestrial feed crops^2,3^, and create highly productive coastal forests that sequester substantial amounts of carbon globally while improving habitat for other marine life^4^. Importantly, macroalgae cultivation does not rely on freshwater, nor compete with arable land or land areas dedicated to conservation, which makes it an attractive source of sustainable biomass. Algal biomass is also rich in micronutrients and proteins, thus has the potential to supplement livestock diets^5^.

The brown macroalga *Saccharina latissima* (‘sugar kelp’) belongs to the *Laminariaceae* family and is widely distributed across temperate coastal waters, including the North Atlantic and North Pacific. Like most brown macroalgae, the cell wall of *S. latissima* is largely comprised of structural polysaccharides, such as alginate, cellulose, and fucoidans, which together account for 30-50% of the algal total dry weight^6^. Depolymerization of alginate is catalyzed by a highly specialized group of polysaccharide lyase (PL) enzymes, called alginate lyases, that cleave glycosidic bonds through β-elimination, resulting in unsaturated products^7^. A number of alginate lyases have been studied from various organisms, primarily originating from marine environments, including marine algae^8^, fungi^9^, viruses^10^, marine mollusks^11,12^ and human gut bacteria^13^. Intriguingly, previous studies have highlighted possible transmission of seaweed-degrading enzymatic machinery from marine bacteria to the human gut microbiome^14–17^. This includes alginate lyases^13^ and polysaccharide utilization loci (PULs) for red-algal galactan degradation in human gut bacteria^15,18^. In most cases, these events have been observed to occur within *Bacteroidota*, a phylum known to harbor complex polysaccharide utilization loci (PULs) or systems dedicated to saccharifying distinct polysaccharides. Exchange of PULs within *Bacteroidota* promotes the acquisition of novel genetic machinery and the ability to subsist on available polysaccharide substrates^19^.

**Fig. 1:**
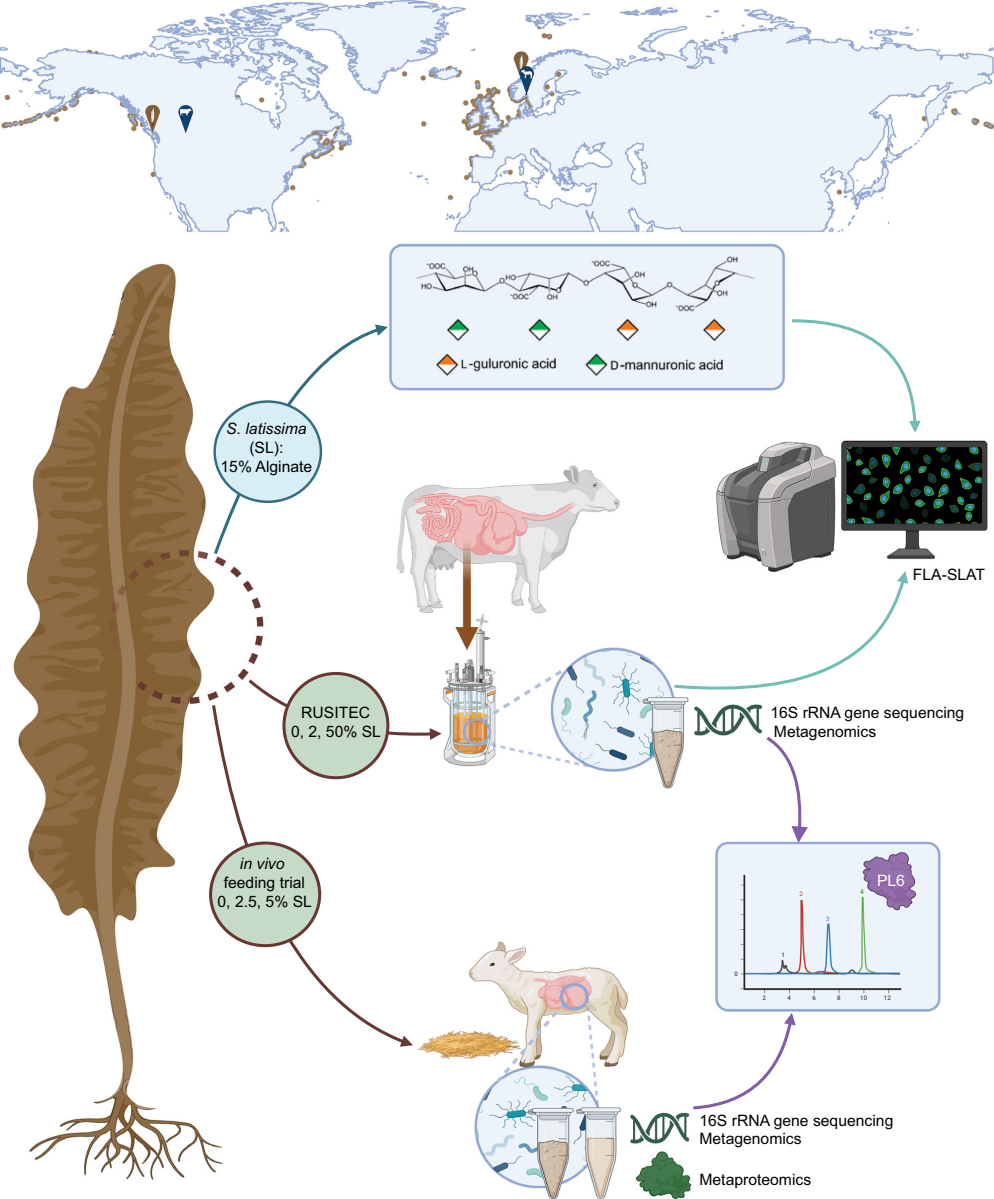
Study design. *Saccharina latissima* is a brown alga commonly found in the North Atlantic, Arctic and Pacific oceans. *S. latissima* was collected from the west coasts of Canada and Norway for microbiome studies. Alginate constitutes a substantial portion of the cell wall of *S. latissima* (SL), and its digestion requires a specific set of enzymes known as alginate lyases. We investigated if and how *S. latissima* is metabolized in geographically distinct rumen ecosystems through in vivo lamb feeding experiments (2.5% and 5.0% inclusion, DM basis) and in vitro cattle-based rumen simulation technique (RUSITEC) experiments with inclusion level up to 50%. Evidence supporting ruminal degradation of alginate was explored using a combination of multilayered meta-omics, physiology (fluorescently labeled *S. latissima* hot water extracts, FLA-SLAT) and biochemical characterization of PL6 alginate lyases. Schematic representation of the methodological framework was created in BioRender. Hagen, L. (2025) https://BioRender.com/mf3zvcw.

In recent years, the practice of adding seaweed to livestock feed has gained momentum due to the potential of certain species to reduce enteric methane emissions^20,21^. However, feeding seaweed to cattle is not a new strategy; farmers in coastal areas have historically collected kelp to supplement livestock feed in years with poor harvests^22^. In some instances, animals have survived almost entirely on seaweed^23,24^. While some ruminant populations, such as the North Ronaldsay sheep, have consumed seaweed for generations, the evolutionary events that endowed intestinal microorganisms with the potential to convert their chemically complex polysaccharides into a source of nutrition for their hosts are as-yet unknown. In this study, we investigated the adaptation of two geographically distinct ruminant microbiomes to dietary kelp alginates. To evaluate microbiome responses, we first analyzed rumen samples from an in vivo feeding experiment, where naïve lambs were fed 2.5% and 5.0% *S. latissima* on dry matter (DM) basis. Through meta-omic analyses, we demonstrate the presence of functionally active rumen-associated *Prevotella* populations with the enzymatic machinery required to catabolize brown seaweed alginate through ‘alginate utilization loci’ (AULs). These results were validated in an unrelated rumen ecosystem using the rumen simulation technique (RUSITEC), where rumen content from cattle was incubated with *S. latissima* at low (2.0%) and high (50%) inclusion rates. Interactions between alginate and microbial cells were confirmed using fluorescently labeled polysaccharides and extracted rumen communities. The activity of *Bacteroidota* AULs was further validated using recombinant enzymes derived from both lamb and cattle datasets (Fig. 1). Alginate-consuming microbial populations were present across different production systems and host species, yet with high identity among alginate lyases within the identified AULs. Our results underscore the remarkable ability of rumen microbial inventory to rapidly adapt to novel and complex polysaccharides.

## Results

### Algal polysaccharides in *S. latissima*

**Fig. 2:**
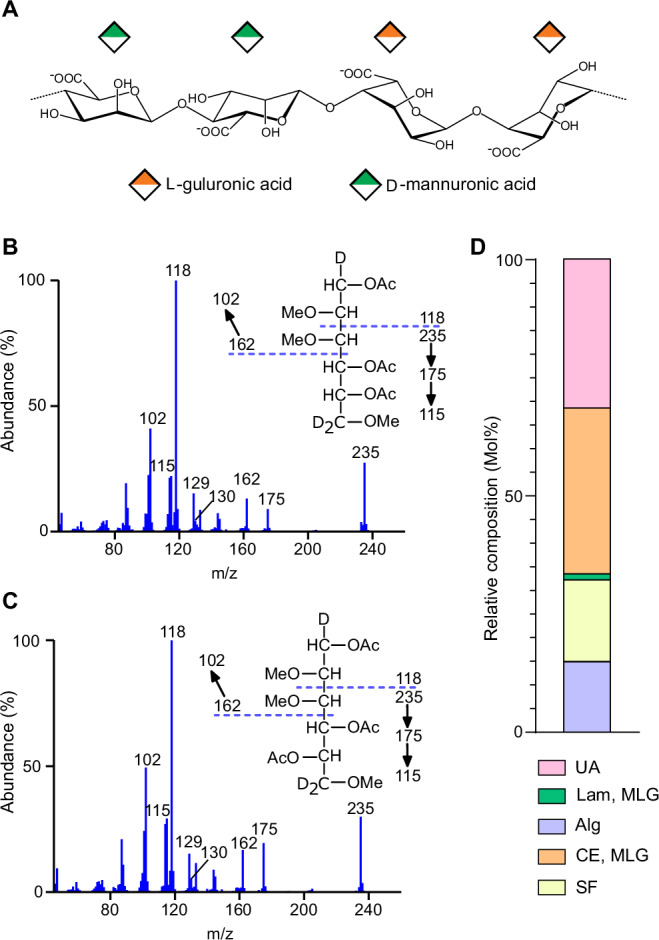
Linkage analysis and polysaccharide estimation of the brown algae *S. latissima* cell walls. **A** Alginate is a high molecular weight, unbranched matrix polymer composed of two uronic acids: α-l-guluronic acid and β-d-mannuronic acid. These are linked by 1,4-glycosidic bonds and arranged in blocks of either homodimers (polyM or polyG) or heterodimers (polyMG). **B** EI-MS spectra and ion fragmentation patterns for 4-Gul*p*A and **C** 4-Man*p*A from PMAAs of *S. latissima* alginate. **D** Estimated polysaccharide composition (Mol%) of cell walls extracted from *Saccharina latissima* (*n* = 2). The following abbreviations were used for their respective polysaccharides and assigned according to the literature^26^: SF (fucoidans – all Fuc*p*, all 2-Man*p*, all 6-Gal*p*, t-Gal*p*, 3,4-Gal*p*, 4-Glc*p*A, 3-Glc*p*A, t-Xyl*p*); CE/MLG (cellulose, mixed-linked glucan – 4-Glc*p* + 4,6-Glc*p* + (t-Glc*p* – 3,6-Glc*p*)); Alg (alginate – all Gul*p*A and Man*p*A linkages); Lam/MLG (laminarin, mixed-linkage glucans – 3-Glc*p* + 3,6-Glcp*2); UA (unassigned – all remaining linkages).

A complete understanding of ruminal kelp digestion requires knowledge of the chemical and structural diversity of kelp polysaccharides. To determine the linkage composition of *S. latissima*, kelp harvested off the west coast of Canada was subjected to GC-MS based glycosidic linkage analysis using a recently established procedure optimized for unfractionated cell wall of brown seaweeds^25^. The identification of all permethylated alditol acetates (PMAAs), including those from 4-Gul*p*A and 4-Man*p*A, was based on their characteristic MS fragmentation patterns and retention times (Fig. 2B, C). The relative abundance of linkages identified from *S. latissima* (Supplementary Table 1; Supplementary Fig. 1) were used to estimate the relative compositions of total cell wall polysaccharides including cellulose, mixed-linkage glucan, laminarin, fucoidan, and alginate (Fig. 2D). Cellulose was found to be the most abundant polysaccharide, as indicated by the dominance of 4-Glc*p*, which is consistent with our comparative analysis of various brown seaweeds^25^. Fucoidans, whose linkages were assigned based on previous study^26^, exhibited a highly diverse linkage pattern, including t-Fuc*p* (2.1%), 2-Fuc*p* (1.2%), 3-Fuc*p* (3.2%), 4-Fuc*p* (1.7%), 2,3-Fuc*p* (1.1%), 2,4-Fuc*p* (0.8%), 3,4-Fuc*p* (2.0%), and 2,3,4-Fuc*p* (5.1%) linkages, together representing 17.3% of the cell wall polysaccharides in *S. latissima*. Alginate constituted 14.9% of the total relative abundance, with Gul*p*A linkages (10.1%) being more prevalent than Man*p*A linkages (4.6%), suggesting an M/G ratio of 0.46. The laminarin composition was calculated to be 1.3%, based on the sum of 3-Glc*p* and 3,6-Glc*p*, multiplied by two to account for the corresponding t-Glc*p* of 3,6-Glc*p* side chains. Of note, mixed-linkage glucan linkages may be confounded with those found in laminarin and cellulose.

### Niche specialization dictates decomposition of brown algae polysaccharides in naïve ruminants

In our initial study of the rumen microbiome from Norwegian White lambs (*n* = 24), 16S rRNA gene analysis was conducted on both fluid and fiber-attached rumen samples obtained from all animal dietary groups participating in the month-long feeding trial, including control, low seaweed (2.5% *S. latissima* on DM basis) and high seaweed (5.0% *S. latissima* on DM basis). As expected, notable differences in the community structure between the fluid and fiber-attached samples were observed across all three dietary groups. However, no definitive evidence indicating a macroalgae-induced alteration in the microbiome structure was identified (Supplementary Fig. 2). In the bovine RUSITEC experiment, where the range of seaweed inclusion was between 2.0% and 50% *S. latissima* (DM basis), significant changes to rumen microbial communities were observed between time points (Supplementary Fig. 3A; ANOSIM and PERMANOVA, *p* = 0.001) and 50% *S. latissima* inclusion (Supplementary Fig. 3B; ANOSIM, *p* = 0.005; PERMANOVA, *p* = 0.008) compared to the control forage. RUSITEC communities supplemented with *S. latissima* also showed lower Shannon and inverse Simpson diversity values compared to control communities, although the Chao1 richness index was not significantly altered over time, or by seaweed supplementation (Supplementary Fig. 3C). Notably, an evident increase in the relative abundance of *Bacteroide*s was seen at the 8-day time point in the 50% *S. latissima* treatment (Supplementary Fig. 3D). The capability of the RUSITEC microbial communities to interact with *S. latissima* polysaccharides was investigated in vivo using fluorescent *S. latissima* extract (FLA-SLAT). Cells within the 2.0% and 50% *S. latissima*-treatments demonstrated uptake of FLA-SLAT, which overlaid with 4’6-diamidino-2-phenylindole (DAPI) DNA staining (Supplementary Fig. 4). In both rumen systems, the digestibility of seaweed was observed with no detrimental impacts (Supplementary Text 1, Supplementary Table 2 and 3).

**Fig. 3:**
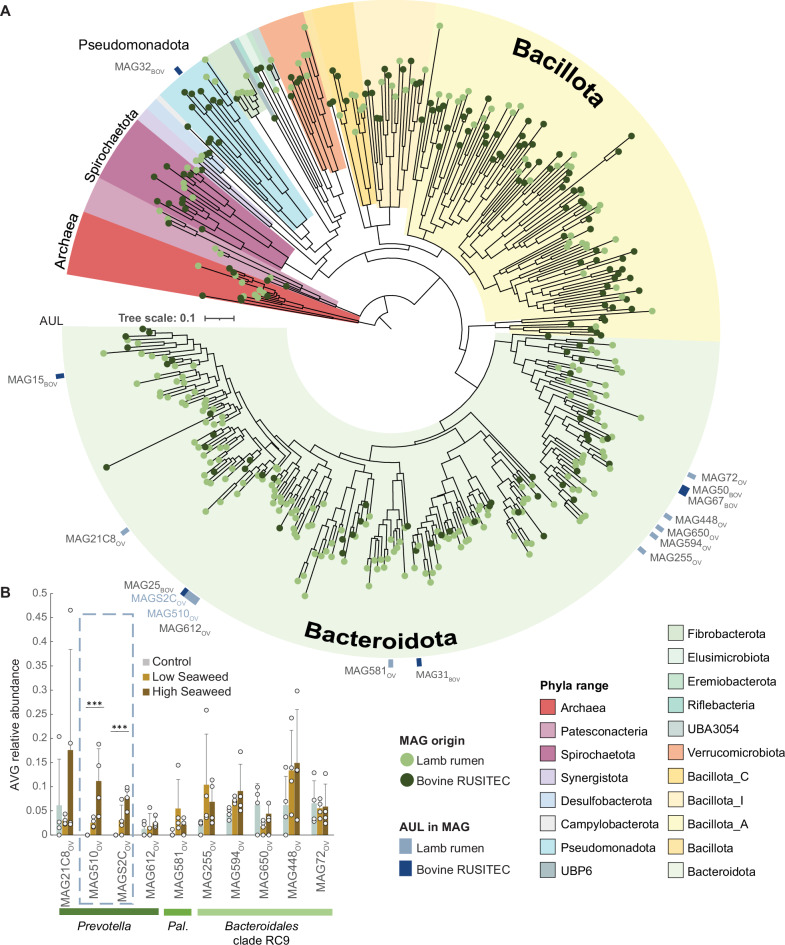
Rumen microbes encode enzymes for alginate utilization. **A** Phylogenetic tree of MAGs recovered from lamb rumen (light green circle on branch) and the bovine RUSITEC (dark green circle on branch) microbiomes. In total 16 MAGs, of which all except one (MAG32_BOV_) are affiliated to the *Bacteroidota*-phylum, encoded AUL for alginate decomposition. **B** Relative abundance of the lamb rumen MAGs encoding alginate lyases were calculated for each sample using CoverM and displayed as the average of each diet group. Error bars denote standard deviation, and individual data points are shown (circles) Two closely related *Prevotella* MAGs harboring AUL (MAGS2C_OV_ and MAG510_OV_) demonstrated dose-dependent increase in relative abundance in response to inclusion of *S. latissima* in the diet. Asterisks indicate significant Spearman correlation between MAG relative abundance and dietary seaweed inclusion level (FDR-adjusted *p* < 0.001). All p-values are provided in Supplementary Table 4. Pal.; *Paludibacteraceae*.

Although the overall rumen community structure seemed unaffected by dietary supplementation of seaweed when given at biologically relevant doses, we wanted to further elucidate the genetic potential to utilize brown seaweed within the microbiomes. For this, shotgun metagenomic sequencing and MAG reconstruction were carried out on lamb rumen samples from all three dietary groups, as well as for the control and 50% *S. latissima-*treated RUSITEC communities. Reconstruction of the lamb rumen metagenome yielded 260 MAGs (named “MAGX_OV_”) of medium to high quality, defined by the MIMAG standard for completeness and contamination^27^. The majority of these MAGs were affiliated with the *Bacteroidota* phylum (*n* = 148), followed by *Bacillota* (*n* = 36) and *Methanobacteriota* (*n* = 7) (Fig. 3A). When investigating the gene content of these MAGs, we identified enzymes annotated as alginate lyases encoded within 28 MAGs (Supplementary Data 1C). Most of these MAGs were associated with members of *Bacteroidota*, along with *Fibrobacterota* and *Verrucomicrobiota*. Due to the structural composition of alginate, alginolytic systems often appear in gene clusters, or AULs, in Gram-negative bacteria; these systems consist of Sus-like proteins and alginate lyases with complementary specificities^16,28,29^. We therefore extended our gene search to include the co-occurrence of neighboring TonB-dependent receptors (SusC-like) and SusD-like substrate-binding proteins, which are archetypical components of PULs^30–32^. This refinement led to the identification of ten *Bacteroidota* MAGs, of which four were classified as *Prevotella* species, five assigned to the *Bacteroidales* RC9 clade, and one to the *Paludibacteriaceae* family (Fig. 3A).

The genomes recovered from the RUSITEC systems accounted for another 132 MAGS (named “MAGX_BOV_”), with a noticeable fraction being *Bacillota* (*n* = 64) and *Bacteroidota* (*n* = 32) (Fig. 3A). Similar to the lamb microbiome, the majority of alginate lyases were found within *Bacteroidota* phylum members originating from the *S. latissima-*treated RUSITEC system (Supplementary Data 1F); six *Bacteroidota* MAGs contained alginate lyases, five of which also harbored intact AULs (Fig. 3A). Notably, an alginate utilization cluster (AUC) was also identified within MAG32_BOV_ classified as *Brevimundis bullata* (87% complete; contamination <1%), which does not have the traditional SusC/D-like system.

Intriguingly, among the lamb-originating MAGs encoding alginate lyases, two closely related *Prevotella*-affiliated MAGs, MAG510_OV_ and MAGS2C_OV_, demonstrated a pronounced increase in relative abundance in response to increasing levels of *S. latissima* in the diet. In contrast to the other potential alginate-degrading genotypes, neither of these two *Prevotella* MAGs was detected in fiber-attached rumen samples from the control diet group (Fig. 3B), further indicating that their presence correlates with *S. latissima* inclusion in the diet. Relative abundance and quality information of all MAGs can be found in Supplementary Data 1A–E.

### Functionally active alginate-consuming *Prevotella* populations

**Fig. 4:**
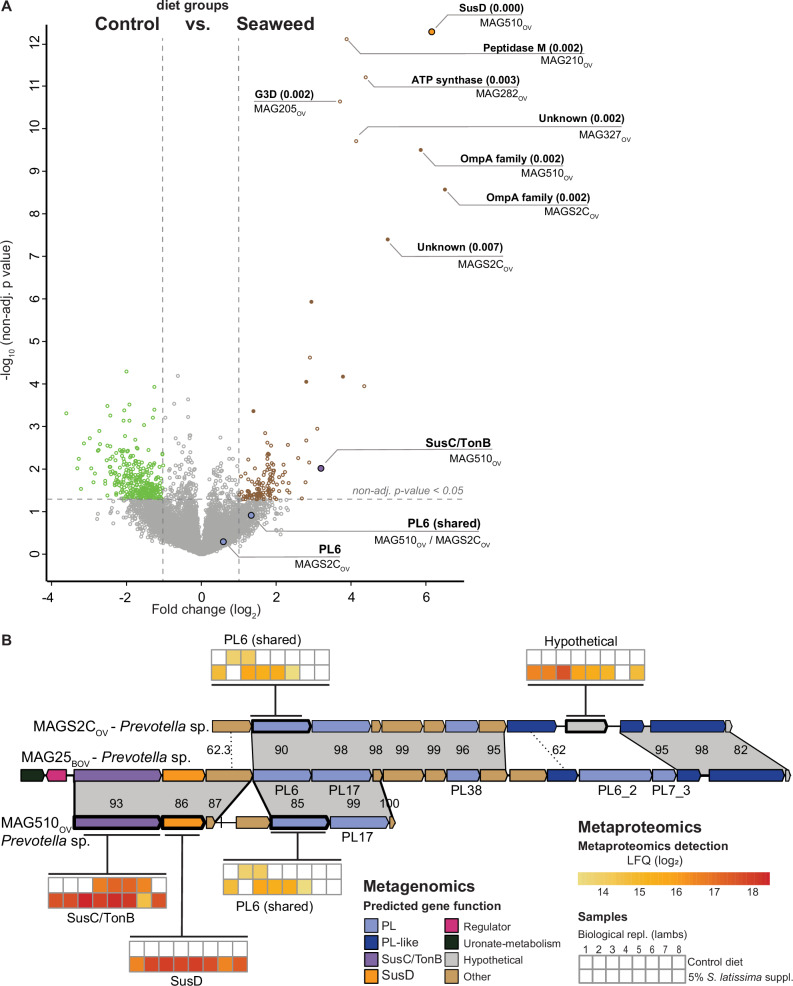
Detection of alginate lyases within AULs. **A** Volcano plot of all protein groups identified in lamb rumen samples demonstrates detection of two alginate lyases (PL6) from *Prevotella* spp. (MAG510_OV_ and MAGS2C_OV_), along with a SusC-like and a significantly higher detected SusD-like protein in the 5% seaweed-supplemented diet group compared to the control group. Protein groups with significant changes in LFQ intensities were identified using a two-sided Student’s T-test with a permutation-based FDR correction (FDR < 0.05, S0 = 0.1). Green circles indicate proteins upregulated (non-adjusted *p* value < 0.05) in the control diet, while brown indicates those upregulated in the seaweed-supplemented diet. Filled circles denote proteins affiliated to selected *Prevotella* MAGs. Significantly (FDR-adjusted *p* < 0.05) upregulated proteins are highlighted with annotations (i.e., function, MAG-affiliation and FDR-adjusted *p* value). **B** According to the predicted gene organization, the proteome-detected PL6 enzymes and SusC/SusD-like proteins were located within AULs that showed high synteny to AULs extracted from the *Prevotella* species MAG25_BOV_ found in the cattle-based RUSITEC microbiome. Gray shading indicates homology and percent identity values. Genes with bolded outlines were detected within the lamb metaproteome, and the heatmaps show the protein intensities (LFQ values for the eight replicates sampled in control and 5% *S. latissima*-fed lambs).

To investigate whether the putative alginate-degrading populations were functionally active, we analyzed the metaproteome of rumen samples from all lambs in the control and high *S. latissima* (5.0%) dietary groups. Our metagenome-centered metaproteomic analysis resulted in the detection of 10,312 protein groups that mapped back to our sample-specific database (Supplementary Data 2A–C). Among these protein groups, we detected two groups annotated as alginate lyases (Fig. 4A), suggesting a role in the depolymerization of alginate. Notably, these two alginate lyases, both PL6, mapped back to the two *Prevotella* MAGs (MAG510_OV_ and MAGS2C_OV_) that demonstrated a dose-dependent increase in relative abundance in response to the inclusion of *S. latissima* in the diet. While the peptides constituting one of the detected PL6 enzymes appeared to uniquely match MAGS2C_OV_, the other PL6 undistinguishably mapped back to protein sequences found in both *Prevotella*-affiliated MAGs. Alignment of the full-length protein sequences within the shared PL6 protein group revealed a high degree of sequence similarity (BLASTp: 96% id), suggesting a common ancestral origin and functional redundancy in their substrate specificity. The label-free quantification (LFQ) of the detected proteins confirmed that the two PL6 enzymes were enriched in the animal group fed with high inclusion of *S. latissima* (Fig. 4B). Of note, in both MAG510_OV_ and MAGS2C_OV_, the genes of the expressed PL6 enzyme were neighboring another gene encoding an enzyme from a known alginate lyase family; PL17 (no protein detection).

In addition to the detection of the PL6 enzyme, a proteome inspection of MAG510_OV_ also unveiled differential expression of a TonB-dependent receptor (SusC-like) and its adjacent SusD-like substrate-binding protein in the 5% *S. latissima* dietary group compared to the control. Specifically, the MAG510_OV_-affiliated SusD-like protein was the most upregulated protein with *S. latissima* supplementation in the entire dataset (Fig. 4A). Another essential component of the Sus system, the SusE-like outer membrane protein, was also detected in the proteome of MAG510_OV_, yet at a lower LFQ detection level. Within a PUL, the *susC-* and *susD*-like genes are typically situated in close proximity to the relevant CAZymes^32^. However, due to genome fragmentation, the *susC/susD*-like genes and the alginate lyases (*pl6* and *pl17*) were found towards the edges on two separate contigs, and thus, a complete AUL could not be determined. Despite their physical separation on different contigs, possibly due to incomplete genome reconstruction, the explicit co-expression of *susC*/*susD* and *pl6* implies that these genes may still function as a part of a co-regulated AUL.

### *Prevotella* spp. AULs in globally separated rumen ecosystems

**Fig. 5:**
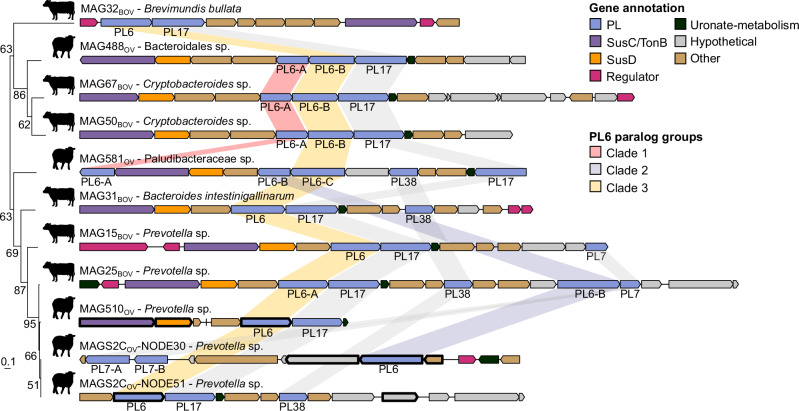
Synteny between lamb and cattle alginate AULs. OrthoFinder was used to generate a species tree composed MAGs containing AUL/AUCs. ClustalOmega, BLAST, and SACCHARIS were used to identify orthologs and similarity between AUL/AUC genes. The AULs and AUC were created with the gggenes package. Bolded genes were identified within the lamb metaproteomics.

We observed that AULs found in *Bacteroidota*, originating from the lamb rumen microbiome and cattle-based RUSITEC system from two different continents, were highly syntenic and contained highly similar sequences to the other AULs. In total, eight *Bacteroidota* phyla members had intact AULs, along with the AUC from the *B. bullata* MAG32_BOV_. All MAGs contained at least one PL6 and PL17 pair, with some containing either additional PL6, PL7, or PL38 sequences within the AULs (Fig. 5). Between RUSITEC and lamb *Bacteroidota* species MAGs, the highest similarity was discovered between *Prevotella* MAGs, specifically the cattle-based RUSITEC MAG25_BOV_, and lamb rumen MAGs MAGS2C_OV_ and MAG510_OV_, showing amino acid identity as high as 99% (Fig. 4B, Supplementary Data 1G). This similarity extends to the alginate lyases, as the PL17 members across the three AULs shared 98% amino acid identity, while the shared PL6 members exhibit 85% identity. The PL38 protein shared between MAG25_BOV_ and MAGS2C_OV_ displayed 96% sequence identity.

**Fig. 6:**
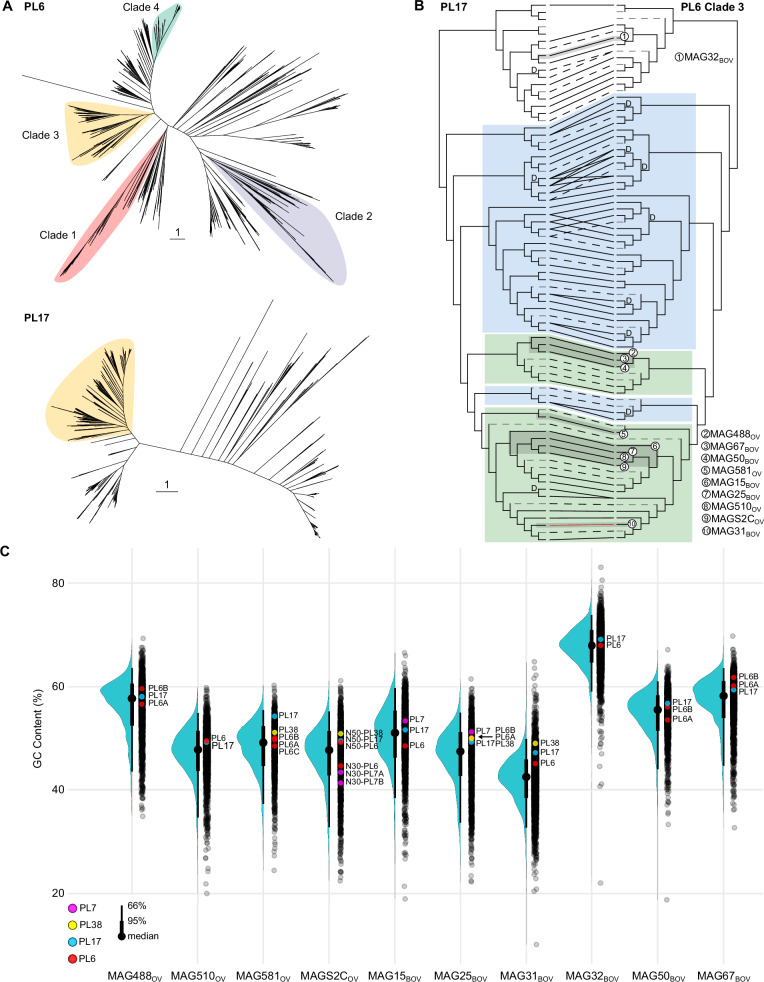
PL6 and PL17 alginate lyases evolved through gene duplication and speciation events. **A** Phylogenetic trees generated from all sequences from PL6 and PL17 within the CAZy database, combined with those predicted from lamb and cattle MAGs. Phylogenetic clades with uncharacterized MAG sequences are shaded and given labels 1–4. **B** Comparison of PL17 and PL6 Clade 3 NOTUNG gene trees. Gene loss (dotted lines) and duplications “D” are marked per species and lines are drawn between trees to connect sequences within the same genome. MAGs identified in this study are shaded gray, while terrestrial *Bacteroidota* species are shaded green and marine *Bacteroidota* species are shaded blue. The full labeled tree is present in Supplementary Fig. 9. **C** GC content of each gene within ruminant MAGs was plotted using half-eye plots made with ggdist^97^. The median is shown as a point, and the 66% and 95% percentile intervals are visualized by the vertical lines on the density plot. Sample size, median and percentile values are provided in Supplementary Table 7. Key alginate PL family members within AULs are highlighted.

Using SACCHARIS 2.0^33^, the PL6 enzymes within *Bacteroidota* AULs, along with homologous PL6s found on shorter contigs in both the RUSITEC and lamb MAGs, partitioned into three distinct clades based on amino acid identity. In addition, the PL6 enzyme found within *B. bullata* MAG32_BOV_ formed a more distantly related group (Clade 4) compared to the other three clades. Aligning these PL6 members to all PL6 enzymes within the CAZy database revealed that Clade 1, 3, and 4 consisted entirely of PL6 subfamily 1 members, while Clade 2 was composed of subfamily 2 members (Fig. 6A; Supplementary Fig. 5 and 6). Members of Clade 1 and 2 differed in structure from those of Clade 3 and 4, as they lacked the predicted inactive C-terminal domain (Supplementary Fig. 7)^34^. Further inspection of the phylogeny showed that PL6 members of both Clade 1 and Clade 2 were most closely related to characterized sequences from *Rhodothermus marinus* DSM 4252. Specifically, Clade 1 members shared the highest sequence similarity (37-40% identity) with Rmar_1165, an enzyme active on poly-MG alginate blocks (consisting of both Guluronate: G and Manuronate: M). Similarly, Clade 2 members were most closely related to Rmar_1386 (38-42%), also active on poly-MG alginate (Supplementary Fig. 5)^35^. A majority of Clade 3 members had the highest sequence similarity (41-48%) to an exo-type alginate lyase of human gut bacterium *Bacteroides clarus*^34^, except MAG581_OV_-C, which was more similar (33.43%) to a poly-MG-active PL6 enzyme characterized from the marine bacterium *Nonlabens ulvanivorans*^35^. Lastly, the MAG32_BOV_ PL6 member shared 55% sequence similarity with an endo-poly-MG alginate active PL6 from *Stenotrophomas maltophilia* KJ-2 (Supplementary Fig. 6)^36^. The dispersed clustering of PL6 contrasted with PL17 members, which shared high similarity and clustered together within subfamily 1 (Fig. 6A).

The similarities between *Bacteroidota* PL6s from the gastrointestinal tracts of terrestrial animals and those found in marine/marine sediment microbiota suggested that some level of gene transfer may be involved in the acquisition of alginate saccharification. NOTUNG^37^ was used to reconcile OrthoFinder species trees with PL17 and PL6s gene trees across 75 genomes from marine environments and terrestrial gastrointestinal tracts (Supplementary Table 5). PL6 members were either separated into paralog clades or kept together as an ortholog group. The presence and absence of PL6 and PL17 members were predicted to be a result of gene duplication and loss events, rather than horizontal gene transfer events (HGT). The only HGT event predicted was observed within Clade 2 between terrestrial host MAGs: MAG581_OV_ (*Paludibacteraceae* sp.) and the *Prevotellaceae* family (MAG25_BOV_ and MAGS2C_OV_) (Supplementary Fig. 8**;** Supplementary Table 6). Among nearly all species analyzed, PL17 and PL6 Clade 3 members were frequently co-located and formed the core component of AULs in *Bacteroidaceae* (Fig. 6B). Furthering this data, the GC content of the AUL/AUC-encoded PLs was comparable to the remainder of their respective genomes (Fig. 6C), suggesting these genes were not part of a HGT event.

**Fig. 7:**
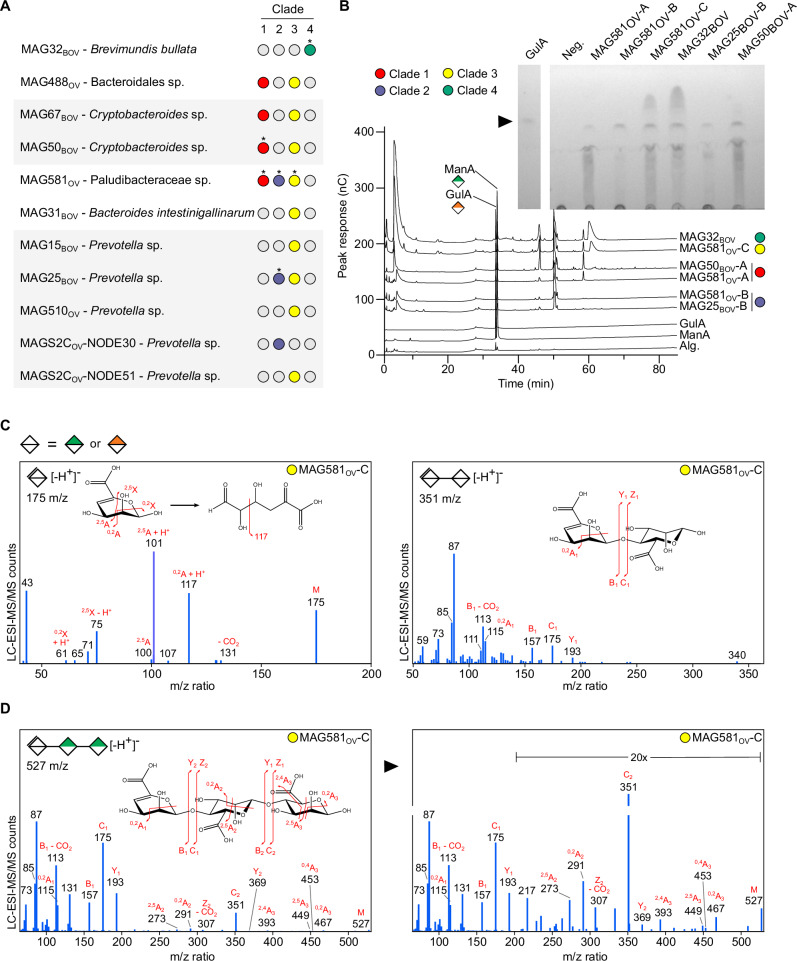
Characterization of alginate oligosaccharide products from *Bacteroidota* species PL6 alginate lyases. **A** Summary of which lamb and rumen MAGs contain AUL/AUCs PL6 members within clades 1–4. Black stars indicate sequences which were selected for further characterization. **B** TLC and HPAEC-PAD product analysis of brown seaweed alginate digested with lamb and cattle-associated MAG PL6 enzymes. ManA and GulA represent monosaccharide standards. **C** Alginate lyase oligosaccharide products were analyzed by LC-ESI-MS/MS. Ions of m/z 175 (left) and 351 (right) corresponding to unsaturated mono- and disaccharide products were observed, and spectra were extracted. ESI-MS/MS with HCD was performed in order to confirm identity of the extracted ions, and MS2 product ion spectra are shown with the carbohydrate fragmentation depicted and identified. **D** MS2 spectra of ion 527, corresponding to unsaturated trisaccharide, identified as ΔHexA-ManA-ManA based on the presence of signature ions^50^. Monosaccharide symbols are displayed according to the Symbol Nomenclature for Glycans system^114^. For monosaccharides unable to be discerned between mannuronate and guluronate by LC-MS, white symbols are used.

Overall, PL6 members of Clade 3 were found in all *Bacteroidota* MAGs encoding AULs, but not within the AUC identified in MAG32_BOV_. Nearly half of the PULs contained multiple PL6 members belonging to different clades, of which the combination of Clade 1 and Clade 3 was most prevalently occurring in both lamb rumen (*Bacteroidales* sp. MAG488_OV_ and *Paludibacteraceae* sp. MAG581_OV_) and cattle-based RUSITEC MAGs (*Cryptobacteriales* spp. MAG50_BOV_ and MAG67_BOV_) (Fig. 7A). The *Prevotella* MAGs seemed to rather combine PL6 Clade 3 with Clade 2 within the same AUL (MAG25_BOV_ and MAG581_OV_), or at separate loci (MAGS2C_OV_). Of note, the two *Prevotella*-affiliated PL6 proteins detected in the metaproteome belonged to Clade 2 and Clade 3 (shared PL6).

To confirm the predicted alginate activity of AUL/AUCs, PL6 members from each clade (Clade 1: MAG581_OV_-A, MAG50_BOV_-A; Clade 2: MAG581_OV_-B, MAG25_BOV_-B; Clade 3: MAG581_OV_-C; Clade 4: MAG32_BOV_) were selected for gene synthesis and functional characterization. All selected PL6 members contained SPI signal peptides, except for MAG50_BOV_-A, which had an SPII signal peptide (not shown, as signal peptides were ultimately removed for protein expression in this study). Enzymes were incubated with alginate from brown seaweed and observed after overnight digestion, as well as continuous direct measurement of unsaturated product formation at 232 nm. Direct measurement was used to determine the optimum pH and initial velocities of the selected PL6 enzymes in the linear range of the reaction (Supplementary Fig. 10). All PL6 members, except MAG581_OV_-B, produced absorbance at 232 nm, indicating enzymatic activity. However, the kinetics of most PL6 enzymes demonstrated inefficient digestion of alginate, with the exception of MAG50_BOV_-A, and Michaelis-Menten could not be accurately fitted (Supplementary Fig. 10F). Despite variations in catalytic efficiencies, all PL6 members produced oligosaccharides when incubated with alginate (Fig. 7B), however only MAG50_BOV_-A sufficiently degraded poly-G and poly-M oligosaccharides (Supplementary Fig. 11). Using High-Performance Anion-Exchange Chromatography with Pulsed Amperometric Detection (HPAEC-PAD) and thin-layer chromatography (TLC), PL6 members from the same clade displayed overlapping activities. LC-MS was used to further characterize the structure of unsaturated oligosaccharides (Fig. 7C, D).

All PL6s produced alginate oligosaccharides of varying degrees of depolymerization. Clade 3 and Clade 4 members produced monosaccharides that appear to decompose through a keto-enol intermediate into 4-deoxy-l-erythro-5-hexoseulose uronate (DEH)^38–40^. Both MAG581_OV_-B and MAG25_BOV_-B appeared less active on brown seaweed alginate, producing only small amounts of alginate oligosaccharides.

## Discussion

The matrix cell wall polysaccharides of brown seaweeds are largely comprised of anionic polysaccharides, such as alginate and fucoidan^6^. Their charge density corresponds with the ionic nature of ocean water and enables metal-dependent, higher-order polymer cell wall dynamics, such as gelation. The polysaccharide composition of *S. latissima* was confirmed to consist of cellulose and mixed-linkage glucans, and the matrix polysaccharides fucoidan and alginate. Alginate comprised approximately 15% of the total plant cell wall polysaccharides in S*. latissima* (Fig. 2D; Supplementary Table 1), which is in agreement with previous reports^25^. Fucoidan comprised ~17% of the estimated polysaccharide content, almost equivalent to that of alginate. *S. latissima* fucoidan is known to be structurally complex, consisting of highly sulfated α-1,3-l-fucan backbones, with side chains composed of β-1,6-d-galactose or alternating β-d-glucuronic acid and α-d-mannuronic acid extensions^26^. Despite the abundance of fucoidan, no putative fucanases were detected from the lamb or bovine RUSITEC meta-omics datasets, suggesting that fucoidan is likely not digested by the rumen microbiomes of either host. To date, the detection of fucanase or fucoidan-specific sulfatase from a terrestrial gut microorganism has not been reported. Whether this stems from its structural complexity, bioavailability, or other factors remains to be determined.

*Bacteroidota* MAGs with the genetic capacity to degrade alginate were reconstructed from the lamb feeding experiment and bovine RUSITEC sequencing data. Intriguingly, MAGs that encoded the capacity to consume alginate included two closely related *Prevotella* strains that exhibited diet-induced dose responses in their relative abundance (Fig. 3). Metaproteomics further confirmed that these *Prevotella* species produced polysaccharide lyases specific for the digestion of alginate (Fig. 4). Reconstruction of the pathway architecture containing these alginate lyases indicated they were functional components of AULs, similar to those previously observed in marine and human gut bacteria^13,16,41^ (Fig. 5). Likewise, PL17 members were found co-located with PL6 subfamily 1 members in Clade 3^13,16^. In contrast, members of PL6 subfamily 2 and subfamily 1 Clade 1 appear to be ancillary components, likely arising from gene duplication events. Furthermore, the detection of elevated levels of SusC-like and SusD-like proteins in the *S. latissima* diet group reinforces the relevance of these AULs for alginate digestion. Surprisingly, a large-scale diet-induced difference was not observed in the overall microbial community structure when evaluated by 16S rRNA gene sequencing. This suggests that a limited number of specific rumen microbial species play roles by occupying a specialized niche for digestion of seaweed-derived polysaccharides. Notably, other PL enzymes associated with alginate consumption were also identified within the AULs, though their expression was not detected. This could be due to several technical factors, such as their amenability to protein extraction methods and their absolute levels being below proteomic detection thresholds. Moreover, a plethora of biological factors could plausibly explain an absence of detection, including alginate specificity and/or percent inclusion level required to necessitate expression, compartmentalized secretion, specificity of induction products (i.e., which digestion products induce AULs)^42,43^, or insufficient adaptation time for efficient utilization of seaweed. Others have suggested that alginate polymers decompose slowly^41,44^.

Increasing the overall alginate concentration to 50% of the total mixed ration (25-fold over the lamb feeding experiment) within the RUSITEC experiment allowed us to discern multiple *Bacteroidota* organisms capable of digesting alginate (Fig. 5). In contrast, *Prevotella* spp. abundance was not significantly different between control and *S. latissima*-supplemented RUSITECs, and, although expected^45^, we identified multiple *Prevotella* spp. within RUSITEC-derived MAGs containing AULs.

To measure direct interactions between polysaccharides and microorganisms in the rumen, we previously developed a technique using fluorescent polysaccharides, or FLAPS^46^. This enables the response of microorganisms to labeled substrates to be quantified at the single-cell level. Accumulation of fluorescent polysaccharide extracts from *S. latissima* (FLA-SLAT) (Supplementary Fig. 4) in microbial cells, provided further support that alginate is consumed and this occurs in response to incubation with *S. latissima*. SR-SIM imaging following FLA-SLAT incubation showed that most interactions with FLA-SLAT occurred in the periphery of cells, which is reminiscent of Gram-negative bacteria that employ a selfish mechanism of foraging^46–48^.

The operonic AUL structures within this study were identified using previously characterized lyase families: PL6, PL7, PL17, and PL38. While members of PL6 have been shown to be both endo- and exo-acting, they typically display an endo-mode of action, indiscriminately cleaving internal glycosidic bonds within the alginate polymer to produce oligosaccharides with C4,C5 unsaturations at their non-reducing ends. In contrast, PL17 enzymes are often categorized as exo-acting oligoalginate lyases that primarily act on the polyM structure at the end of an alginate chain, releasing unsaturated mono- or disaccharides of mannuronate^13^. Previous studies of alginate-consuming marine and human gut bacteria have shown that PL6 and PL17 members are frequently co-located, strongly suggesting that they may operate in a coordinated manner to efficiently depolymerize alginate^13,16^. In addition to PL6 and PL17, some MAGs encoded alginate lyases belonging to the PL7 and PL38 families. To confirm the function of representative enzymes from these AULs, several PL6 members were selected for biochemical characterization. PL6 enzymes can possess “vanguard” status by catalyzing the first stages of alginate depolymerization at the cell surface^49^. Several recombinant PL6 enzymes were shown to produce substantial amounts of unsaturated products by TLC, HPAEC-PAD, and LC-MS. Due to the complexity of M/G orientation and ratio, the ability of PL6 enzymes to change mode of action (endo/exo) depending on either alginate substrate (poly-M or poly-G)^35^, and the absence or presence of non-catalytic domains^34^, precise activity was not demonstrated within this study. Notably, delineation between ManA and GulA signatures is often indistinguishable using methods other than NMR; however, prediction of some oligosaccharide structures is possible based upon a previous study that defined patterns of differential MS2 fragmentation abundance of alginate oligosaccharides. For example, an identified unsaturated tri-saccharide was found to contain signature ions (m/z of 307, and a high ratio of 449/453), suggesting the oligosaccharide product consists of a ΔHexA-ManA-ManA^50^ (Fig. 6C). Further, EI-MS and MS2 fragmentation revealed that some uronic acid residues of alginate may be sulfated (Supplementary Fig. 12). This unexpected result warrants further study.

Seaweed polysaccharides tend to be structurally and chemically complex, highly charged, and absent from terrestrial plants typically consumed by livestock. Members of the human microbiome are known to have catabolic pathways dedicated to the digestion of polysaccharides from red^14,17,18^ and brown^13^ seaweeds. Multiple hypotheses, including the ‘sushi factor’^15,17^ and ancient transfer^13^, have been proposed to explain the origin of these pathways in gut microbiomes. In contrast to humans, much less is known about the mechanisms by which livestock microbiomes adapt to the introduction of exotic dietary polysaccharides, such as alginate from brown seaweed. Previously, alginate-consuming *Prevotella* spp. were isolated from North Ronaldsay sheep on the Orkney Islands, which are known for their diet enriched with brown seaweeds, likely species of *Laminaria*, *Fucus*, and *Ascophyllum*^23^. Recent studies have also revealed that wild Svalbard reindeer populations are adapting to the warming Arctic climate by supplementing their diet with kelp, driven by an increasing prevalence of ice-locked pastures^51^. How these animals survived a radical change in their diet and lifestyle, and the evolutionary processes that drove microbial adaptation towards seaweed polysaccharide catabolism, have not been thoroughly studied.

Alginate is produced by *Pseudomonas* spp. found natively within the rumen^52,53^. However, it differs from seaweed alginates, containing lower M/G ratios and high levels of O-2, O-3 acetylation of mannuronic acid^53,54^. Therefore, although we show that dietary *S. latissima* results in expression of AULs, it remains to be shown if alginate produced by autochthonous bacteria help drive the evolution of these pathways. It has been previously speculated that xanthan gum utilization loci in human gut *Ruminococcaceae* evolved under selection of exopolysaccharides before being exposed to structural analogs found in food additives^55^. Likewise, it is possible that structurally related alginate substrates present a common niche for microbiota to fill in the rumen. One distinguishing factor is the absence of carbohydrate esterases in the seaweed-alginate associated MAGs, which is a conceivable requirement for bacterial alginate metabolism, suggesting there is differential specificity^13,56^. It is unclear from our data how adaptation to alginate effects other microorganisms, as lower Shannon diversity with the 50% RUSITEC trial (Supplementary Fig. 3) suggests overall lower microbial diversity and increasing algal content did not result in a noticeable shift in community structure. This suggests alginate does not function as a prebiotic in the rumen. In another study, it was found that carrageenan supplementation drastically altered the fecal microbiome composition, but not the rumen microbiome composition^57^. Thus, the role of the lower gut microbiota in alginate digestion should be explored in future work.

Here, we discover and characterize the structure of syntenic AULs responsible for alginate catabolism from both cattle RUSITEC and lamb microbial communities exposed to dietary *S. latissima*. Alginate catabolism appears to be a core functional trait performed by autochthonous members of the ruminant microbiome^58^, indicating that these genomic loci were not recently transferred from the surface microbiome of *S. latissima* or another marine ecosystem. The conservation of PL6 and PL17 pairs within *Bacteroidota* species from geographically and taxonomically distinct ruminants suggests that these genetic loci were acquired through one or a series of evolutionary events, possibly via speciation or duplication. However, there is little evidence of horizontal gene transfer. In this light, perhaps AULs may have evolved in an ancestor common to humans and cattle. Despite conservation in function, there is noticeable structural variation in the gene content and organization of AULs (Fig. 5). Regardless of how individual pathways assembled, the overall functional conservation of AULs within the ruminant microbiomes is clearly maintained throughout lineages and between populations. In this manner, the maintenance of seaweed alginate catabolism in the absence of substrate availability represents a “latent trait” within the rumen microbiome. Often, these pathways are encoded within the genomes of saccharolytic generalists, which likely provides a mechanism for retention of alginate competent strains when these substrates become scarce. Ultimately, this provides a nutritional benefit to the host and justifies the fitness cost required to maintain this unique catalytic machinery within *Bacteroidota* species^59,60^. Regardless, acquisition and maintenance of a diverse metabolic capacity appears to position the animal to rapidly adapt to abrupt changes in its diet and provides tantalizing clues into the ancient foraging habits of its ancestors.

The adaptation of livestock to novel feedstocks may provide a solution to pressures associated with climate change and land use competition with food crops. Wild and cultivated seaweed represent novel sources of feed ingredients for ruminant livestock; however, how and at what rate ruminant microbiomes can adapt to the introduction of new carbon sources may dictate the overall feasibility of their incorporation into production systems. Here, using molecular informatic approaches, we investigated the ability of two geographically and taxonomically distinct ruminant microbiomes to adapt to *S. latissima*, a widely distributed, commercially grown brown seaweed. Our study demonstrates that rumen *Bacteroidota* species contain highly conserved AULs that are inherited through vertical transmission from ancestral species, and are upregulated in *S. latissima* supplemented diets. Indeed, these PULs appear to be native to the ruminant microbiome, not the marine environment, and are capable of digesting dietary alginate. This suggests that ruminant-associated microorganisms may have adapted to other exotic polysaccharides and novel forages by the retention of latent traits that are carried within the microbiome of ruminant livestock.

## Methods

### Ethics statement

The lamb feeding experiment was conducted at The Livestock Production Research Center in Ås, Norway. The experiment was approved by the committee overseeing the rules and regulations governing animal experiments in Norway under the surveillance of the Norwegian Food Safety Authority (FOTS-ID: 16406). Donor heifers used in the RUSITEC experiment were cared for in accordance with the guidelines of the Canadian Council on Animal Care (2009) and were approved by the Institutional Animal Care and Use Committee (ACC2304).

### Preparation of unfractionated cell wall of *S. latissima*

*S. latissima* was harvested from the waters near Vancouver Island, B.C., Canada on May 1st, 2022. The cell wall extractions were prepared in accordance to Avci, Pattathil^61^ with modifications according to previous reports^25,62^. Approximately 100 mg of ball-milled dry sample was extracted in 40 mL of absolute ethanol (EtOH) for 8 h, followed by three 8 h extractions in 40 mL of 80% (v/v) EtOH, and then two 20 min washes with absolute EtOH. After each alcohol extraction and wash, samples were centrifuged at 3000 × *g* for 30 min. The final alcohol insoluble residue (AIR) was vacuum-dried by SpeedVac (Thermo Scientific, USA).

The sampling locations for *S. latissima* are illustrated in Fig. 1, along with an overview of its distribution across the Northern Hemisphere. Procedures for generating the distribution map are detailed in Supplementary Text 2.

### Glycosidic linkage analysis of the cell wall

The AIR of *S. latissima* was analyzed as described Bajwa, Xing^25^. Briefly, polysaccharides in dry AIR power (10 mg) were methylated by 1.2 mL of methyl iodide in 2 mL of dimethyl sulfoxide in the presence of excess sodium hydroxide^63,64^. The methylation product was suspended in dichloromethane (DCM) and partitioned against 10% acetic acid in water (v/v) over ice once then against deionized water three times. After each partitioning, the upper phase was collected and pooled. Following the final partitioning, the lower phase was evaporated to dryness, and the pooled upper phase was mixed with the evaporated lower phase. The mixture was dialyzed with a molecular weight cut-off of 6000–8000 Da against 0.1 M triethylamine hydrochloride (TEAH) deionized water solution, then against pure deionized water, followed by freeze-drying. Two additional rounds of methylation were conducted with the 0.1 M TEAH dialysis step omitted^25^. The permethylated sample was converted to partially methylated alditol acetates (PMAAs) by hydrolysis in 2 mL of 4 M trifluoroacetic acid (TFA) at 100 °C for 4 h, reduction with 20 mg of sodium borodeuteride (NaBD_4_, 99 atom % D, Alfa Aesar) in 2 mL of deionized water for 16 h^65^, and acetylation by heating in a mixture of acetic anhydride and TFA (5:1, v/v) at 60 °C for 1 h^25^. In a separate experiment, the dry AIR powder (10 mg) was subjected to weak methanolysis (0.5 M methanolic HCl, 80 °C, 20 min), followed by NaBD_4_ reduction to convert uronic acids to their corresponding 6,6’-dideuterated neutral sugars^66–68^. Excess NaBD_4_ was neutralized by acetic acid, followed by evaporation of the product to dryness and repeated evaporation in 10% acetic acid in methanol (v/v), then in absolute methanol to remove borate, before the sample was converted to PMAAs^25^. All PMAAs were analyzed using an Agilent 7890A-5977B GC-MS system (Agilent Technologies, Santa Clara, CA, USA) coupled to a medium-polarity Supelco SP-2380 column (60 m × 0.25 mm × 0.2 µm; Sigma-Aldrich, USA) under a consistent flow of helium at 0.8 mL/min. Inlet temperature was maintained at 250 °C. The sample was injected with a 10:1 split ratio. The oven program started at 120 °C (hold 1 min) and then increased at 3 °C/min to 200 °C (hold 50 min) then at 3 °C/min to 250 °C (hold 20 min). The PMAAs were identified by comparing their EI-MS fragmentation patterns and retention times to those of prepared PMAA standards^25^ and also by referencing the literature (Carpita and Shea, 1989). Relative molar linkage compositions of the PMAAs were determined from the TIC chromatogram, following the principle that a PMAA’s quantity correlates with the ratio of its TIC peak area to its molecular weight, as described in the published protocol^69^. The peak corresponding to the PMAA of 4-Glcp was used to normalize the relative abundances of neutral sugar PMAAs in the untreated sample and the PMAAs derived from uronic acid linkages in the sample pretreated with weak methanolysis-NaBD₄ reduction^25^. Three separate experiments were conducted to the AIR sample.

### In vivo experimental design

A total of 24 lambs (*Ovis aries*) of the commercial breed Norwegian White were used in the in vivo feeding experiment, as previously explained in detail by Grabež, Devle^70^. In brief, weaned ewe lambs with average age of 132 days at start of the experiment and body weight 37.3 ± 1.6 kg were randomly assigned to three dietary treatment groups (*n* = 8 per group). All lambs had free access to clean drinking water and were fed the experimental diet *ad libitum* twice a day (at 08:00 h and 14:00 h) in individual pens (76 cm×157 cm, with plastic slatted floors). The control group (no seaweed included) were fed a total mixed ration of grass silage and compound feed (DRØV Lam produced by Norgesfôr AS, Norway), rolled barley, and mineral premix (VitaMineral ® Normal Sau produced by NORMIN, Norway). The two other treatment groups were additionally supplemented with 2.5% and 5.0% *S. latissima* on a dry matter (DM) basis. The *S. latissima* used in the in vivo feeding experiment was harvested by Seaweed Solution AS (Trondheim, Norway) at their cultivation site outside Frøya, Norway (collected May 28^th^ 2018), and immediately frozen. Prior to the feeding experiment, the seaweed biomass was chopped to uniform particle size and wilted, before being stored frozen again until final preparation of experimental diets. The experimental period lasted for 35 days, during which the lamb growth rate, daily feed intake and feed dietary composition were regularly monitored.

### RUSITEC experimental design

The RUSITEC experiment followed a 2 × 2 + 1 factorial design with two experimental runs and two treatments. Each RUSITEC unit was equipped with eight 920 mL fermenters, designated with three replicates per treatment (*n* = 6) in each unit across both runs. Here, the basal substrate consisted of a 50:50 (DM basis) barley silage and barley straw diet. The two dietary treatments included control (no seaweed included) and *S. latissima* (collected from Vancouver Island, B.C., Canada, May 1st 2022) included at 2% dietary DM. An additional fermenter in each run was fed 50% *S. latissima* for microbial profiling. The seaweed replaced equal proportions of barley silage and barley straw, and the treatment was randomly allocated to a RUSITEC fermenter in each unit. Prior to the experiment, the barley straw, barley silage, and seaweeds were dried at 55 °C for 48 h and then ground through a 4 mm screen using a Wiley Mill (standard model 4; Arthur H. Thomas Co., Philadelphia, PA, USA). A total of 10 g DM was included in nylon bags (R1020; 10 × 20 cm; 50 ± 10 μM porosity; 107 R1020, ANKOM Technology, Macedon, NY, USA) for incubation within the two RUSITEC units.

Rumen inoculum was obtained from three ruminally cannulated Angus-cross heifers (*Bos taurus*), 16 months of age, that had previously adapted to a barley silage and barley straw-based diet. The rumen contents were collected 2 h pre-feeding from four different sites within the rumen and squeezed through PECAP mesh (mesh size 250 µm; PA66CG-250 136 cm, Sefar Nytal, Gilbert Saguenay, QC, CA). Both the solid and liquid proportions were pooled in equal proportions from each heifer, and transported to the laboratory in an insulated thermos kept at 39 °C. All fermenters were maintained at 39 °C in a water bath before filling with 180 mL of pre-warmed McDougall’s buffer (McDougall, 1948), and 720 mL of double-strained rumen fluid was added under a stream of CO_2_ to maintain anaerobic conditions. One R1020 Ankom bag filled with 20 g (wet weight) of mixed rumen solids, and one bag with the dietary treatment, were added to each fermenter. The fermenters were then fitted within each RUSITEC unit, contained within a circulating water bath retained at 39 °C. After 24 h, the bag containing the rumen solids was replaced with a dietary treatment bag, such that each day the bag incubated for 48 h was replaced with a new bag. Artificial saliva was continuously infused into fermenters (26 mL h^–1^) using a peristaltic pump set to achieve a dilution rate of 2.9% h^–1^. The effluent and gas were collected within 2 L Erlenmeyer flasks, and 4 L reusable gas tight collection bags Curity®; Conviden Ltd., Mansfield, MA, USA, respectively, connected through a closed tubing system. The RUSITEC experimental period lasted for 15 days, with days 1–7 used for adaptation and days 8–15 used for sampling and measurements. The DM, OM, CP, and NDF degradability, and total VFA concentration was determined from day 8–15 and analysis was conducted as previously described in Terry, Krüger^71^. The capability of the RUSITEC microbial communities to interact with *S. latissima* polysaccharides was investigated in vivo using fluorescent *S. latissima* extract (FLA-SLAT), as described in Supplementary Text 3.

### Microbial sampling and DNA extraction

Samples for microbial analysis were collected from both the RUSITEC experiment and the in vivo feeding trial. These samples were subsequently used for 16S rRNA and shotgun metagenomics.

From each RUSITEC fermenter, 2 mL of liquor was collected at day 0, 1, 8, and 15. Samples were centrifuged at 20,000 × *g* for 20 min and supernatants were removed. Pellets were suspended in 1 mL of DNA/RNA Shield™ (Zymo Research, USA) before genomic extractions were performed using the DNeasy PowerSoil Pro kit (Qiagen, Canada) following the manufacturer’s protocols. DNA was stored at –80 °C until analysis.

In the in vivo feeding trial, rumen samples were collected at the end point of the experiment period. Here, all animals were slaughtered at a commercial slaughterhouse (Rudshøgda, Nortura SA, Norway) and their intact gastrointestinal tracts were directly moved to a working bench. The stomach was opened, and the reticulo-rumen content was hand-mixed prior to sampling. The mixed sample was transferred into a sterile strainer blender bag (0.50 mm pore size; Stomacher® 400 Seward BA 6041, Worthing, UK) and gently squeezed to separate the fluid and particle phases. The phases were then sampled into cryotubes, transported in liquid nitrogen to the laboratory and stored at − 80 °C until analysis. Upon microbiome analysis, samples from both fluid and particle phases were thawed on ice and homogenized by vortexing prior to cell lysis and DNA extraction. For cell lysis, 0.25 g homogenized biomass was weighted out for bead-beating using a FastPrep-24 Homogenizer (MP Biomedicals LLC., Ohio, USA) at 4 m/s for 45 s. DNA were extracted using DNeasy PowerLyzer PowerSoil kit (Qiagen, Hilden, Germany) following the manufacturer’s protocol. The extracted DNA were quantified using a Qubit Fluorimeter and the Qubit dsDNA BR Assay Kit (ThermoFisher Scientific, Waltham, MA, USA) and stored at –80 °C until further use.

### 16S rRNA gene sequencing

In the RUSITEC experiment, the purified DNA samples were sent to Génome Québec (Montréal, QC, Canada) for Illumina MiSeq PE250 16S rRNA gene sequencing using the primers: 515 F (5’-GTGCCAGCMGCCGCGGTAA-3’) and 806 R (5’-GGACTACHVGGGTWTCTAAT-3’) targeting the V4 region of the 16S rRNA gene. Paired-end reads were quality trimmed using Trimmomatic^72^ with a sliding window of 5:20, and adapters were automatically removed. Trimmed FASTA files were merged and classified using Kraken 2^73^ according to the Silva Database release 138.1^74^. Bracken^75^ was used to estimate bacterial and archaeal abundance at the genus level.

Liquid and particle phase samples from all 24 lambs were collected for the amplification of the 16S rRNA gene. The 16S rRNA gene was PCR-amplified using the primers: Pro341F (5’-CCTACGGGNBGCASCAG-3’) and Pro805R (5’-GACTACNVGGGTATCTAATCC-3’)^76^, which targets the V3-V4 region of the 16S rRNA gene. The 25 μL PCR reactions consisted of 1× iProof High-Fidelity Master Mix (Bio-Rad, Hercules, CA, USA), 2.5 μL each primer, and 7.5 μL template DNA. PCR thermal cycling began with a hot start step at 98 °C for 3 min, and they were followed by 25 cycles of 98 °C denaturation for 30 s, 53 °C annealing for 30 s and 72 °C extension for 30 s, followed by a final extension step at 72 °C. The amplicon libraries were purified with AMPure XP beads (Beckman Coulter, Indianapolis, IN, USA) and indexed with the Nextera XT Index Kit v2 (Illumina, San Diego, CA, USA). Equimolar concentrations of the libraries were pooled together, and further purified with AMPure XP beads. The pooled library was quantified using a Qubit Fluorometer, diluted, and denatured before being sequenced on an Illumina MiSeq sequencing system using 2 × 300 bp cycle runs with the MiSeq reagent kit v3. The generated 16S rRNA gene sequences were processed using the DADA2 pipeline^77^ in R v4.3.2^78^ which included quality control, elimination of primers and adapters. The sequences were clustered into Amplicon Sequence Variance (ASVs) and the ASV identified were taxonomically assigned using SILVA Database release 138.1. Downstream analysis of lamb and RUSITEC communities are described in detail in Supplementary Text 4.

### Metagenomics

To reconstruct a core rumen microbial genome catalog from each experimental group of lambs, extracted DNA from the corresponding fluid and particle from four animals in each diet group (*n* = 24 samples in total) was subjected to metagenome sequencing. The selection of these representative samples was guided by diversity and abundance metrics from the 16S rRNA gene analysis. Shotgun metagenomic sequencing was performed at the Norwegian sequencing center (Oslo, Norway), where the libraries were prepared using PCR-free TruSeq chemistry and sequenced on two lanes of the Illumina HiSeq 4000 to generate 2 × 150 bp pair-ended reads. From the RUSITEC system, DNA from samples collected on experimental day 15 was sent for shotgun metagenomics at Génome Québec (Montréal, QC, Canada). These samples were sequenced on an Illumina NovaSeq6000 platform, also generating 2 ×150 bp pair-ended reads.

### Generation of metagenome-assembled genomes (MAGs)

RUSITEC sequences were trimmed with Trimmomatic v0.39 and assembled using metaSPAdes v3.13.0^79^. Assembled contigs were binned using the MetaWRAP v1.3.2^80^ binning and bin-refinement pipeline, using CONCOCT v1.1.0^81^, Maxbin2 v2.2.6^82^, and Metabat2 v2.12.1^83^. Bins were dereplicated using dRep v3.2.2^84^ and filtered for 50% completion and under 10% contamination. CheckM v1.1.3^85^ was used to evaluate the quality, while MAG abundance was estimated using CoverM v0.6.1 (https://github.com/wwood/CoverM). MAGs were annotated using dbCAN v3.0.7^86^ and Bakta v1.9.3^87^. GTDB-Tk v2.4.0^88^ was used to infer taxonomic classification.

All metagenome raw reads from lamb samples were quality filtered using Trimmomatic v0.36 in pair-end mode before the trimmed reads were assembled into contigs. Both individual assemblies and co-assemblies of all samples were carried out metaSPAdes v3.13.0 and MegaHIT v1.2.9^89^, respectively. Contigs from the single assembly were binned using VAMB v3.0.2^90^, while the contigs from the co-assembly were binned using both MetaBAT2 v2.12.1 and MaxBin2 v2.2.7. Collectively, these three binning strategies generated 1,532 bins across all samples. These bins were re-dereplicated at 99% ANI using dRep v3.2.2, which resulted in 291 dereplicated bins, hereafter referred to as metagenome-assembled genomes (MAGs), with completeness above 50% and contamination below 25%. This collection of MAGs was used as a genome database for metagenome-centric metaproteome analysis, allowing for the retention of genetic information without compromising the sensitivity and false discovery rate estimation^91^. For biological interpretation, only MAGs with contamination <10% were considered. CheckM v1.1.3 was used to evaluate the quality parameters of each MAG, while CoverM v0.6.1 in genome mode was used to estimate the MAG abundance in each sample. Spearman’s rank correlation was used to assess relationship between MAG relative abundance and dietary seaweed inclusion levels (0%, 2.5% and 5.0%). Correlation coefficients and raw p-values were calculated in R using cor.test(), with p-values adjusted using Benjamini-Hochberg (FDR). Effect size is reported as Spearman’s rho. Taxonomically classification of the MAGs was assigned using GTDB-Tk v2.4.0, and function annotation carried out using dbCAN, PFAM, and KEGG, integrated in the DRAM v1.2.4^92^ annotation tool.

### AUL and AUC phylogenetic analysis

AULs from lamb and RUSITEC data were manually screened for SusC-like and SusD-like pairings. Syntenic AULs were compared using Diamond BLASTp v2.1.8^93^ and Clustal Omega^94^, and alignments were visualized using RStudio and the gggenes package^95^. MAG species trees were generated using OrthoFinder v2.5.5^96^. CAZyme (PL6 and PL17) phylogeny was completed using SACCHARIS 2.0 v2.0.0.dev19. RStudio packages ggdist^97^, gghalves^98^, and ggrepel^99^ was used to generate GC content plots.

In total 75 genomes (Supplementary Table 5) from marine environments and terrestrial gastrointestinal tracts, including AUL and AUC containing MAGs from this study, were selected based on presence of AULs (PULDB^100^) or PL6 and PL17 family members. In addition, NCBI reference strains without alginate-targeting CAZymes were included in the analysis. All genomes were run through OrthoFinder to generate a species tree (Supplementary Fig. 13). Additionally, genomes were analysed using dbCAN to identify putative PL6 and PL17 members, and SACCHARIS to identify ortholog clustering in the case of PL6 family members. Gene trees were generated by first generated alignments with MUSCLE v5.1^101^, followed by trimming poorly aligned regions with trimAI v1.5.0^102^ using the -automated1 option. The best-fit substitution model was determined with ModelTest—NG v0.1.7^103^, and phylogenetic trees were subsequently generated with RAxML-NG v1.2.2^104^. NOTUNG v3.0-beta^37^ was used to generate gene species tree alignments using default cost parameters, while RecPhyloXML^105^ was used to generate a summary of species-gene tree reconciliations. All gene reconciliations are shown in Supplementary Fig. 14.

### Metaproteomics

#### Protein extraction and sequencing

Sample preparation for metaproteomic analysis was carried out using the particle phase of rumen samples collected from all lambs in the control and 5% *S. latissima* experimental group. Lysis buffer (10 mM DTT, 100 mM Tris-HCl (pH = 8) and 4% SDS) and 4 mm glass beads ( ≤ 160 μm) were added to 500 μL of rumen samples, followed by brief mix and resting on ice for 30 min. Mechanical cell lyses was performed using FastPrep-24 Classic Grinder (MP Biomedical, Ohio, USA) for 3 × 45 s at 6.5 m s^–1^. Samples were then centrifuged at 16,000 × *g* for 15 min at 4 °C and lysate was transferred to a new tube, followed by a clean-up using Wessel-Flügge precipitation^106^. The precipitated proteins were dissolved in SDS-PAGE buffer, heated in the water bath for 5 min at 95 °C and ran on SDS-PAGE Any-kD Mini-PROTEAN TGX Stain-Free gels (Bio-Rad, California, USA) for 3 min. The gel was stained using Coomassie Blue R-250 and visual protein bands were carefully cut into 1×1 mm pieces. After washing the gel pieces, reduction of disulfides was carried out by incubation proteins in 10 mM DTT for 30 min at 56 °C, followed by carbamidomethylation by incubation with 55 mM iodoacetamide for 30 min in the dark, at room temperature. Subsequently, proteins were digested into peptides using trypsin. Peptides were concentrated and eluted using C18 stage tips, before dried on a SpeedVac, re-suspended in 0.1% formic acid, and quantified using a Nanodrop One instrument. Finally, the peptide samples were processed using a nano LC-MS/MS coupled to a timTOF Pro mass spectrometer (Bruker, Germany), as described in detail in Supplementary Text 5.

#### (Meta)genome-centric metaproteomics data analysis

The MS raw data were analysed using FragPipe v16.3, with the search engine MSFragger v3.3^107^ embedded, in addition to Philospopher v4.0.0^108^. Here, a protein sequence catalog retrieved from the 291 recovered MAGs, viral scaffolds (described in Supplementary Text 6), and publicly available rumen eukaryote genomes previously described in Andersen, Altshuler^109^, were used as a sample specific database. Common contaminants such as human keratin, bovine serum albumin and host genome (*Ovis Aries*) were included in the database, as well as decoy sequence entries based on the reverse protein sequences. Carbamidomethylation was used as fixed modification, while oxidation of methionine and protein N-terminal acetylation were added as variable modifications. Trypsin was used as digestive enzyme, with maximum one missed cleavage allowed. For label-free-quantification (LFQ), IonQuant was applied with FDR-controlled match-between-runs (MBR) enabled, followed by global median normalization of intensities across all runs^110^. Protein groups detected from FragPipe were uploaded to Perseus v1.6.15.0^111^, for further downstream analyses. First, protein groups identified as contaminants and host proteins were removed, and the intensities were log_2_(x) transformed. Furthermore, a protein group needed to be detected in minimum three of eight biological replicates in at least one of the experimental diet groups (control and/or 5% inclusion of *S. latissima*) to be considered. For statistical analysis in Perseus, missing values were imputed using the normal distribution-based approach with a width of 0.3 and a downshift of 2.8 relative to the distribution of measured intensities. Protein groups with significant changes in LFQ intensities were identified using a two-sided Student’s T-test with a permutation-based FDR correction (FDR < 0.05, S0 = 0.1). To account for differences in organism abundance, organism-level normalization was performed for target enzymes. For each MAG and sample, the linear LFQ intensities of the target protein were divided by the total LFQ intensities for all proteins assigned to that MAG. Normalized values are provided in Supplementary Data 2D.

#### Biochemical analysis of PLs

PL6 enzymes from *Bacteroidota* species AULs, and an outgroup OTU, *Brevundimonas Bullata* that does not have a traditional SusC/D-like system, were selected for their taxonomic and phylogenetic diversity within the PL6 family. Selected PL6 genes were trimmed to remove the signal peptide and synthesized into pET28a with a C-terminal His_6_-tag and codon optimized for *E. coli* production (Biobasic). Expression plasmids were transformed into BL21 (DE3) Tuner competent cells (Novagen) and grown to an OD_600nm_ 0.6–0.8 in LB Miller broth containing 50 μg mL^−1^ kanamycin before induction with 1 mM IPTG at 18 °C overnight. Induced cultures were pelleted at 6500 x *g* for 20 min and lysed using a combination of lysis buffer (20 mM Tris pH 8.0, 500 mM NaCl, 0.1 mg mL^–1^ lysozyme) and sonication (2 min of 1 s intervals of medium intensity sonic pulses at a power setting of 30 – Fisherbrand Model 705 Sonic Dismembrator and probe; Thermo Fisher Scientific). Cell lysate was centrifuged at 17,500 x *g* for 1 h, and the filtrate was purified using Ni-NTA resin columns (Cytiva) and immobilized metal affinity chromatography. Recombinant protein was eluted with an imidazole gradient in 20 mM Tris, pH 8. Recombinant PL6 enzymes were concentrated and dialyzed into 20 mM Tris pH 8.0, 500 mM NaCl, and 2% glycerol using 30 kDa cut-off Amicon Ultra centrifugal concentrators (Millipore Sigma).

50 nM of recombinant PL6 enzymes was incubated with 5 mg mL^–1^ brown seaweed alginic acid (A1112; Sigma), in 20 mM of varying buffers to determine pH optima at 37 °C: citrate-phosphate (pH 4–8.5), CHES (pH 8.5–10), Glycine (pH 8.5–11), and CAPs (pH 10–11). PL6 β-elimination reaction and the generation of C4-C5 unsaturation can be monitored with absorbance at 232 nm. Reactions were run for 10 min while continuously monitoring absorbance at 232 nm at 30 s intervals using a SpectraMax ID3 plate reader. Initial velocities of PL6 enzyme activity on brown seaweed alginates were determined using eight concentrations of substrate at the optimal pH for each enzyme. Rates of product formation were calculated in GraphPad Prism (9.0.2) using the extinction coefficient 6,150 M^–1^ cm^–1^ to convert absorbance to product concentration^112^. Due to substrate complexity in molecular weight, M/G ratio and organization, velocities were unable to be fit to the Michaelis-Menten equation.

1 µM of recombinant PL6 enzymes was incubated with 5 mg mL^–1^ brown seaweed alginic acid (Sigma), Mannuronate oligosaccharides DP20-35 (Elicityl) and Guluronate oligosaccharides DP25-45 (Elicityl). Reactions on brown seaweed alginic acid were ethanol precipitated by incubating in 50% EtOH overnight at –20 °C before centrifugation at 12,000 x *g* for 10 min and retrieving the supernatant to remove larger, undigested alginate polymers. Samples were dried on a SpeedVac to remove EtOH and resuspended in ultrapure 18 MΩ cm^–1^ H_2_O. Digests were run on TLC using a mobile phase of 2:1:1 butanol: acetic acid: H_2_O, and visualized using an orcinol solution (70:3 acetic acid: sulfuric acid, and 1% orcinol). HPAEC-PAD was performed on a Dionex ICS-3000 using a 3 × 150 mm CarboPac PA20 column (Thermo Scientific). Digests were eluted at a 0.35 mL min^–1^ flowrate at 30 °C in a background of 100 mM NaOH in an increase sodium acetate gradient (0–20 min, 0–0 M; 20–80 min, 0–1 M).

Liquid chromatography was performed on a Vanquish ultra-high performance liquid chromatography (UHPLC) system (Thermo Scientific). Alginate oligosaccharide from brown seaweed alginic acid digest samples were prepared in water and injected in a volume of 10 µL at a concentration of 200 µg/mL. Separation of the digested oligosaccharides was achieved using an Acquity UPLC BEH Amide (HILIC) Column, 130 Å, 1.7 µm, 2.1 mm × 150 mm (Waters) at a flow rate of 300 µL/min at 30 °C, using a gradient as shown in Supplementary Table 8^113^. Electrospray ionization mass spectrometry (ESI-MS) was performed on an Orbitrap Fusion Tribrid system (Thermo Scientific) in negative ion mode. Mass spectra parameters are shown in Supplementary Table 9. To select ions for MS2 experiments, an intensity threshold filter was employed with a minimum intensity of 25,000 and a maximum intensity of 1E + 20, and a dynamic exclusion filter was used after 1 time for 2.5 s with default mass tolerance values. Higher-energy collisional dissociation (HCD) was employed to generate fragments. MS spectra were analyzed using Xcalibur and Freestyle software packages (Thermo Scientific), and product ions were identified based on both manual interpretation and comparison to previous studies^50^.

A list of acronyms and abbreviations used in this paper is provided in Supplementary Table 10.

### Reporting summary

Further information on research design is available in the Nature Portfolio Reporting Summary linked to this article.

## Supplementary information

Supplementary information

Description of Additional Supplementary Files

Dataset 1

Dataset 2

Reporting Summary

Transparent Peer Review file

## Source data

Source Data

## Data Availability

The raw metagenomic sequencing data, metagenomes and metagenome-assembled genomes (MAGs) have been deposited to the Sequence Read Archive (SRA) under the project accession numbers PRJEB83690 for the lamb in vivo feeding experiment and PRJNA1200888 for the bovine RUSITEC experiment. The MAGs are additionally publicly available via Figshare for lamb rumen MAGs [10.6084/m9.figshare.28024343.v1] and bovine RUSITEC MAGs [10.6084/m9.figshare.28024394.v1]. The proteomics data, including the complete database, have been deposited in the ProteomeXchange Consortium (http://proteomecentral.proteomexchange.org) via the PRIDE partner repository with the dataset identifier PXD059090. All LC-MS data is deposited to the GlycoPost repository under the ID GPST000612. Source data are provided with this paper.

## Code availability

The codes generated in this study are available at Zenodo [10.5281/zenodo.16326647].

## References

[1] Ahmar, S., Hensel, G. & Gruszka, D. CRISPR/Cas9-mediated genome editing techniques and new breeding strategies in cereals—current status, improvements, and perspectives. *Biotechnol. Adv.* **69**, 108248 (2023). 37666372 10.1016/j.biotechadv.2023.108248

[2] Hughes, A. D., Kelly, M. S., Black, K. D. & Stanley, M. S. Biogas from macroalgae: Is it time to revisit the idea?. *Biotechnol. Biofuels* **5**, 86 (2012). 23186536 10.1186/1754-6834-5-86 PMC3542030

[3] Kraan, S. Mass-cultivation of carbohydrate rich macroalgae, a possible solution for sustainable biofuel production. *Mitig. Adapt Strat Gl.* **18**, 27–46 (2013).

[4] Krause-Jensen, D. & Duarte, C. M. Substantial role of macroalgae in marine carbon sequestration. *Nat. Geosci.* **9**, 737–742 (2016).

[5] Makkar, H. P. S. et al. Seaweeds for livestock diets: A review. *Anim. Feed Sci. Tech.* **212**, 1–17 (2016).

[6] Lafeuille, B., Tamigneaux, E., Berger, K., Provencher, V. & Beaulieu, L. Variation of the nutritional composition and bioactive potential in edible macroalga cultivated from atlantic canada subjected to different growth and processing conditions. *Foods* **12**, 1736 (2023). 37107531 10.3390/foods12081736 PMC10137355

[7] Lombard, V. et al. A hierarchical classification of polysaccharide lyases for glycogenomics. *Biochem J.* **432**, 437–444 (2010). 20925655 10.1042/BJ20101185

[8] Inoue, A. & Ojima, T. Functional identification of alginate lyase from the brown alga Saccharina japonica. *Sci. Rep.* **9**, 4937 (2019). 30894645 10.1038/s41598-019-41351-6 PMC6426991

[9] Pilgaard, B., Vuillemin, M., Holck, J., Wilkens, C. & Meyer, A. S. Specificities and synergistic actions of novel PL8 and PL7 alginate lyases from the marine fungus Paradendryphiella salina. *J. Fungi (Basel)* **7**, 80 (2021). 33503820 10.3390/jof7020080 PMC7911691

[10] Suda, K., Tanji, Y., Hori, K. & Unno, H. Evidence for a novel Chlorella virus-encoded alginate lyase. *FEMS Microbiol. Lett.* **180**, 45–53 (1999). 10547443 10.1111/j.1574-6968.1999.tb08776.x

[11] Hata, M. et al. Comparative study on general properties of alginate lyases from some marine gastropod mollusks. *Fish. Sci.* **75**, 755–763 (2009).

[12] Nisizawa, K., Fujibayashi, S. & Kashiwabara, Y. Alginate lyases in the hepatopancreas of a marine mollusc, Dolabella auricula Solander. * J. Biochem.* **64**, 25–37 (1968). 5707811 10.1093/oxfordjournals.jbchem.a128859

[13] Mathieu, S. et al. Ancient acquisition of “alginate utilization loci” by human gut microbiota. *Sci. Rep. -Uk* **8**, 8075 (2018). 10.1038/s41598-018-26104-1 PMC5966431 29795267

[14] Pudlo, N. A. et al. Diverse events have transferred genes for edible seaweed digestion from marine to human gut bacteria. *Cell Host Microbe* **30**, 314–328.e311 (2022). 35240043 10.1016/j.chom.2022.02.001 PMC9096808

[15] Hehemann, J. H., Kelly, A. G., Pudlo, N. A., Martens, E. C. & Boraston, A. B. Bacteria of the human gut microbiome catabolize red seaweed glycans with carbohydrate-active enzyme updates from extrinsic microbes. *P Natl. Acad. Sci. USA* **109**, 19786–19791 (2012). 10.1073/pnas.1211002109 PMC3511707 23150581

[16] Ronne, M. E. et al. Three alginate lyases provide a new gut isolate with the ability to grow on alginate. *Appl Environ. Micro* **89**, e0118523 (2023). 10.1128/aem.01185-23 PMC10617595 37791757

[17] Hehemann, J. H. et al. Transfer of carbohydrate-active enzymes from marine bacteria to Japanese gut microbiota. *Nature* **464**, 908–912 (2010). 20376150 10.1038/nature08937

[18] Pluvinage, B. et al. Molecular basis of an agarose metabolic pathway acquired by a human intestinal symbiont. *Nat. Commun.* **9**, 1043 (2018). 29535379 10.1038/s41467-018-03366-x PMC5849685

[19] Coyne, M. J., Zitomersky, N. L., McGuire, A. M., Earl, A. M. & Comstock, L. E. Evidence of extensive DNA transfer between bacteroidales species within the human gut. *mBio* **5**, e01305–e01314 (2014). 24939888 10.1128/mBio.01305-14 PMC4073490

[20] Abbott, D. W. et al. Seaweed and seaweed bioactives for mitigation of enteric methane: Challenges and opportunities. *Anim. -Basel* **10**, 2432 (2020). 10.3390/ani10122432 PMC7766277 33353097

[21] Meo-Filho, P., Ramirez-Agudelo, J. F. & Kebreab, E. Mitigating methane emissions in grazing beef cattle with a seaweed-based feed additive: Implications for climate-smart agriculture. *Proc. Natl. Acad. Sci.* **121**, e2410863121 (2024). 39621924 10.1073/pnas.2410863121 PMC11648605

[22] Evans, F. D. & Critchley, A. T. Seaweeds for animal production use. *J. Appl Phycol.* **26**, 891–899 (2014).

[23] Williams, A. G., Withers, S. & Sutherland, A. D. The potential of bacteria isolated from ruminal contents of seaweed-eating North Ronaldsay sheep to hydrolyse seaweed components and produce methane by anaerobic digestion in vitro. *Micro Biotechnol.* **6**, 45–52 (2013). 10.1111/1751-7915.12000 PMC3815384 23170956

[24] Hansen, H. R., Hector, B. L. & Feldmann, J. A qualitative and quantitative evaluation of the seaweed diet of North Ronaldsay sheep. *Anim. Feed Sci. Tech.* **105**, 21–28 (2003).

[25] Bajwa, B. et al. Characterization of unfractionated polysaccharides in brown seaweed by methylation-GC-MS-based linkage analysis. *Mar. Drugs* **22**, 464 (2024). 39452872 10.3390/md22100464 PMC11509683

[26] Bilan, M. I. et al. Further studies on the composition and structure of a fucoidan preparation from the brown alga Saccharina latissima. *Carbohydr. Res.* **345**, 2038–2047 (2010). 20701899 10.1016/j.carres.2010.07.009

[27] Bowers, R. M. et al. Minimum information about a single amplified genome (MISAG) and a metagenome-assembled genome (MIMAG) of bacteria and archaea. *Nat. Biotechnol.* **35**, 725–731 (2017). 28787424 10.1038/nbt.3893 PMC6436528

[28] D’Souza, G. et al. Interspecies interactions determine growth dynamics of biopolymer-degrading populations in microbial communities. *Proc. Natl. Acad. Sci.* **120**, e2305198120 (2023). 37878716 10.1073/pnas.2305198120 PMC10622921

[29] Lu, D. et al. Biochemical characteristics and synergistic effect of two novel alginate lyases from Photobacterium sp. FC615. *Biotechnol. Biofuels* **12**, 260 (2019). 31700543 10.1186/s13068-019-1600-y PMC6827250

[30] Terrapon, N., Lombard, V., Gilbert, H. J. & Henrissat, B. Automatic prediction of polysaccharide utilization loci in Bacteroidetes species. *Bioinformatics* **31**, 647–655 (2014). 25355788 10.1093/bioinformatics/btu716

[31] Naas, A. E. et al. Do rumen Bacteroidetes utilize an alternative mechanism for cellulose degradation?. *mBio* **5**, e01401–e01414 (2014). 25096880 10.1128/mBio.01401-14 PMC4128358

[32] Martens, E. C., Koropatkin, N. M., Smith, T. J. & Gordon, J. I. Complex glycan catabolism by the human gut microbiota: The Bacteroidetes Sus-like paradigm. *J. Biol. Chem.* **284**, 24673–24677 (2009). 19553672 10.1074/jbc.R109.022848 PMC2757170

[33] Fraser, A. S. C. et al. SACCHARIS v2: Streamlining prediction of carbohydrate-active enzyme specificities within large datasets. *Methods Mol. Biol.* **2836**, 299–330 (2024). 38995547 10.1007/978-1-0716-4007-4_16

[34] Wang, B., Dong, S., Li, F. L. & Ma, X. Q. Structural basis for the exolytic activity of polysaccharide lyase family 6 alginate lyase BcAlyPL6 from human gut microbe Bacteroides clarus. *Biochem Biophys. Res Commun.* **547**, 111–117 (2021). 33610038 10.1016/j.bbrc.2021.02.040

[35] Mathieu, S., Henrissat, B., Labre, F., Skjåk-Bræk, G. & Helbert, W. Functional exploration of the polysaccharide lyase family PL6. *Plos One* **11**, e0159415 (2016). 27438604 10.1371/journal.pone.0159415 PMC4954714

[36] Lee, S. I., Choi, S. H., Lee, E. Y. & Kim, H. S. Molecular cloning, purification, and characterization of a novel polyMG-specific alginate lyase responsible for alginate MG block degradation in Stenotrophomas maltophilia KJ-2. *Appl Microbiol Biotechnol.* **95**, 1643–1653 (2012). 22805784 10.1007/s00253-012-4266-y

[37] Stolzer, M. et al. Inferring duplications, losses, transfers and incomplete lineage sorting with nonbinary species trees. *Bioinformatics* **28**, i409–i415 (2012). 22962460 10.1093/bioinformatics/bts386 PMC3436813

[38] Kawai, S. & Hashimoto, W. 4-Deoxy-l-erythro-5-hexoseulose uronate (DEH) and DEH reductase: key molecule and enzyme for the metabolism and utilization of alginate. *Molecules* **27**, 338 (2022). 35056653 10.3390/molecules27020338 PMC8778563

[39] Mori, T. et al. Falsirhodobacter sp. alg1 harbors single homologs of endo and exo-type alginate lyases efficient for alginate depolymerization. *Plos One* **11**, e0155537 (2016). 27176711 10.1371/journal.pone.0155537 PMC4866713

[40] Hobbs, J. K. et al. KdgF, the missing link in the microbial metabolism of uronate sugars from pectin and alginate. *Proc. Natl. Acad. Sci. USA* **113**, 6188–6193 (2016). 27185956 10.1073/pnas.1524214113 PMC4896693

[41] Koch, H. et al. Biphasic cellular adaptations and ecological implications of degrading a mixture of algal polysaccharides. * ISME J.* **13**, 92–103 (2019). 30116038 10.1038/s41396-018-0252-4 PMC6298977

[42] Robb, C. S. et al. Metabolism of a hybrid algal galactan by members of the human gut microbiome. *Nat. Chem. Biol.* **18**, 501–510 (2022). 35289327 10.1038/s41589-022-00983-y

[43] Luis, A. S. et al. Dietary pectic glycans are degraded by coordinated enzyme pathways in human colonic Bacteroides. *Nat. Microbiol* **3**, 210–219 (2018). 29255254 10.1038/s41564-017-0079-1 PMC5784806

[44] Zhu, Y. M. et al. Complete genome sequence and transcriptomic analysis of a novel marine strain reveals the mechanism of brown algae degradation. *Sci. Rep.* **6**, 38248 (2016). 27901120 10.1038/srep38248 PMC5128808

[45] Fu, T., Pan, L., Shang, Q. & Yu, G. Fermentation of alginate and its derivatives by different enterotypes of human gut microbiota: Towards personalized nutrition using enterotype-specific dietary fibers. *Int J. Biol. Macromol.* **183**, 1649–1659 (2021). 34048831 10.1016/j.ijbiomac.2021.05.135

[46] Klassen, L. et al. Quantifying fluorescent glycan uptake to elucidate strain-level variability in foraging behaviors of rumen bacteria. *Microbiome* **9**, 23 (2021). 33482928 10.1186/s40168-020-00975-x PMC7825182

[47] Reintjes, G., Arnosti, C., Fuchs, B. M. & Amann, R. An alternative polysaccharide uptake mechanism of marine bacteria. * ISME J.* **11**, 1640–1650 (2017). 28323277 10.1038/ismej.2017.26 PMC5520146

[48] Hehemann, J. H. et al. Single cell fluorescence imaging of glycan uptake by intestinal bacteria. * ISME J.* **13**, 1883–1889 (2019). 30936421 10.1038/s41396-019-0406-z PMC6776043

[49] Briggs, J. A., Grondin, J. M. & Brumer, H. Communal living: Glycan utilization by the human gut microbiota. *Environ. Microbiol* **23**, 15–35 (2021). 33185970 10.1111/1462-2920.15317

[50] Zhang, Z. et al. Sequence analysis of alginate-derived oligosaccharides by negative-ion electrospray tandem mass spectrometry. *J. Am. Soc. Mass Spectrom.* **17**, 621–630 (2006). 16503152 10.1016/j.jasms.2006.01.002

[51] Hansen, B. B. et al. Reindeer turning maritime: Ice-locked tundra triggers changes in dietary niche utilization. *Ecosphere* **10**, e02672 (2019).

[52] Kibegwa, F. M. et al. Diversity and functional analysis of rumen and fecal microbial communities associated with dietary changes in crossbreed dairy cattle. *Plos One* **18**, e0274371 (2023). 36638091 10.1371/journal.pone.0274371 PMC9838872

[53] Urtuvia, V., Maturana, N., Acevedo, F., Peña, C. & Díaz-Barrera, A. Bacterial alginate production: an overview of its biosynthesis and potential industrial production. *World J. Microbiol Biotechnol.* **33**, 198 (2017). 28988302 10.1007/s11274-017-2363-x

[54] Remminghorst, U. & Rehm, B. H. Bacterial alginates: From biosynthesis to applications. *Biotechnol. Lett.* **28**, 1701–1712 (2006). 16912921 10.1007/s10529-006-9156-x

[55] Ostrowski, M. P. et al. Mechanistic insights into consumption of the food additive xanthan gum by the human gut microbiota. *Nat. Microbiol* **7**, 556–569 (2022). 35365790 10.1038/s41564-022-01093-0 PMC11537241

[56] Cha, Q. Q. et al. Comparison of alginate utilization pathways in culturable bacteria isolated from Arctic and Antarctic marine environments. *Front Microbiol* **12**, 609393 (2021). 33584613 10.3389/fmicb.2021.609393 PMC7874173

[57] Tingley J. P., et al. Global distribution of microbial carrageenan foraging pathways reveals widespread latent traits within the genetic “dark matter” of ruminant intestinal microbiomes. *bioRxiv*, 1-46 (2025). 10.1038/s41467-026-70776-7 PMC13168452 42120383

[58] Henderson, G. et al. Rumen microbial community composition varies with diet and host, but a core microbiome is found across a wide geographical range. *Sci. Rep.* **5**, 14567 (2015). 26449758 10.1038/srep14567 PMC4598811

[59] Feng, J. et al. Polysaccharide utilization loci in Bacteroides determine population fitness and community-level interactions. *Cell Host Microbe* **30**, 200–215.e212 (2022). 34995484 10.1016/j.chom.2021.12.006 PMC9060796

[60] Wu, M. et al. Genetic determinants of in vivo fitness and diet responsiveness in multiple human gut Bacteroides. *Science* **350**, aac5992 (2015). 26430127 10.1126/science.aac5992 PMC4608238

[61] Avci, U., Pattathil, S. & Hahn, M. G. Immunological approaches to plant cell wall and biomass characterization: immunolocalization of glycan epitopes. *Methods Mol. Biol.* **908**, 73–82 (2012). 22843390 10.1007/978-1-61779-956-3_7

[62] Wood, J. A. et al. Genetic and environmental factors contribute to variation in cell wall composition in mature desi chickpea (Cicer arietinum L.) cotyledons. *Plant Cell Environ.* **41**, 2195–2208 (2018). 29532951 10.1111/pce.13196

[63] Badhan, A. et al. Mechanistic insights into the digestion of complex dietary fibre by the rumen microbiota using combinatorial high-resolution glycomics and transcriptomic analyses. *Comput Struct. Biotechnol. J.* **20**, 148–164 (2022). 34976318 10.1016/j.csbj.2021.12.009 PMC8702857

[64] Bajwa, B., Xing, X. H., Terry, S. A., Gruninger, R. J. & Abbott, D. W. Methylation-GC-MS/FID-based glycosidic linkage analysis of unfractionated polysaccharides in red seaweeds. *Mar. Drugs* **22**, 192 (2024). 38786583 10.3390/md22050192 PMC11122361

[65] Low, K. E. et al. Combinatorial glycomic analyses to direct CAZyme discovery for the tailored degradation of canola meal non-starch dietary polysaccharides. *Microorganisms* **8**, 1888 (2020). 33260318 10.3390/microorganisms8121888 PMC7761036

[66] Muhidinov, Z. K. et al. Characterization of two types of polysaccharides from roots growing in Tajikistan. *Food Hydrocoll.* **105**, 105768 (2020).

[67] Hosain, N. A. et al. Isolation, structure elucidation, and immunostimulatory activity of polysaccharide fractions from Boswellia carterii frankincense resin. *Int J. Biol. Macromol.* **133**, 76–85 (2019). 30981779 10.1016/j.ijbiomac.2019.04.059

[68] Chong, H. H., Cleary, M. T., Dokoozlian, N., Ford, C. M. & Fincher, G. B. Soluble cell wall carbohydrates and their relationship with sensory attributes in Cabernet Sauvignon wine. *Food Chem.* **298**, 124745 (2019). 31260966 10.1016/j.foodchem.2019.05.020

[69] Pettolino, F. A., Walsh, C., Fincher, G. B. & Bacic, A. Determining the polysaccharide composition of plant cell walls. *Nat. Protoc.* **7**, 1590–1607 (2012). 22864200 10.1038/nprot.2012.081

[70] Grabež, V. et al. Sugar kelp (Saccharina latissima) seaweed added to a growing-finishing lamb diet has a positive effect on quality traits and on mineral content of meat. *Foods* **12**, 2131 (2023). 37297376 10.3390/foods12112131 PMC10253089

[71] Terry, S. A. et al. Evaluation of Rumen Fermentation and Microbial Adaptation to Three Red Seaweeds Using the Rumen Simulation Technique. *Anim. -Basel* **13**, 1643 (2023). 10.3390/ani13101643 PMC10215394 37238073

[72] Bolger, A. M., Lohse, M. & Usadel, B. Trimmomatic: a flexible trimmer for Illumina sequence data. *Bioinformatics* **30**, 2114–2120 (2014). 24695404 10.1093/bioinformatics/btu170 PMC4103590

[73] Wood, D. E., Lu, J. & Langmead, B. Improved metagenomic analysis with Kraken 2. *Genome Biol.* **20**, 257 (2019). 31779668 10.1186/s13059-019-1891-0 PMC6883579

[74] Quast, C. et al. The SILVA ribosomal RNA gene database project: improved data processing and web-based tools. *Nucleic Acids Res.* **41**, D590–D596 (2012). 23193283 10.1093/nar/gks1219 PMC3531112

[75] Lu J., Breitwieser F. P., Thielen P., Salzberg S. L. Bracken: estimating species abundance in metagenomics data. *Peerj Comput Sci*, e104 (2017). 10.7717/peerj-cs.104 PMC12016282 40271438

[76] Takahashi, S., Tomita, J., Nishioka, K., Hisada, T. & Nishijima, M. Development of a prokaryotic universal primer for simultaneous analysis of and using next-generation sequencing. *Plos One* **9**, e105592 (2014). 25144201 10.1371/journal.pone.0105592 PMC4140814

[77] Callahan, B. J. et al. DADA2: High-resolution sample inference from Illumina amplicon data. *Nat. Methods* **13**, 581–583 (2016). 27214047 10.1038/nmeth.3869 PMC4927377

[78] R Core Team. R: A language and environment for statistical computing. R Foundation for Statistical Computing (2023).

[79] Nurk, S., Meleshko, D., Korobeynikov, A. & Pevzner, P. A. metaSPAdes: A new versatile metagenomic assembler. *Genome Res* **27**, 824–834 (2017). 28298430 10.1101/gr.213959.116 PMC5411777

[80] Uritskiy, G. V., DiRuggiero, J. & Taylor, J. MetaWRAP-a flexible pipeline for genome-resolved metagenomic data analysis. *Microbiome* **6**, 158 (2018). 30219103 10.1186/s40168-018-0541-1 PMC6138922

[81] Alneberg, J. et al. Binning metagenomic contigs by coverage and composition. *Nat. Methods* **11**, 1144–1146 (2014). 25218180 10.1038/nmeth.3103

[82] Wu, Y. W., Simmons, B. A. & Singer, S. W. MaxBin 2.0: An automated binning algorithm to recover genomes from multiple metagenomic datasets. *Bioinformatics* **32**, 605–607 (2016). 26515820 10.1093/bioinformatics/btv638

[83] Kang, D. D. et al. MetaBAT 2: An adaptive binning algorithm for robust and efficient genome reconstruction from metagenome assemblies. *PeerJ* **7**, e7359 (2019). 31388474 10.7717/peerj.7359 PMC6662567

[84] Olm, M. R., Brown, C. T., Brooks, B. & Banfield, J. F. dRep: A tool for fast and accurate genomic comparisons that enables improved genome recovery from metagenomes through de-replication. * ISME J.* **11**, 2864–2868 (2017). 28742071 10.1038/ismej.2017.126 PMC5702732

[85] Parks, D. H., Imelfort, M., Skennerton, C. T., Hugenholtz, P. & Tyson, G. W. CheckM: Assessing the quality of microbial genomes recovered from isolates, single cells, and metagenomes. *Genome Res* **25**, 1043–1055 (2015). 25977477 10.1101/gr.186072.114 PMC4484387

[86] Zheng, J. et al. dbCAN3: Automated carbohydrate-active enzyme and substrate annotation. *Nucleic Acids Res.* **51**, W115–W121 (2023). 37125649 10.1093/nar/gkad328 PMC10320055

[87] Schwengers, O. et al. Bakta: rapid and standardized annotation of bacterial genomes via alignment- free sequence identification. *Micro Genomics* **7**, 000685 (2021). 10.1099/mgen.0.000685 PMC8743544 34739369

[88] Chaumeil, P.-A., Mussig, A. J., Hugenholtz, P. & Parks, D. H. GTDB-Tk: A toolkit to classify genomes with the Genome Taxonomy Database. *Bioinformatics* **36**, 1925–1927 (2019). 31730192 10.1093/bioinformatics/btz848 PMC7703759

[89] Li, D., Liu, C.-M., Luo, R., Sadakane, K. & Lam, T.-W. MEGAHIT: An ultra-fast single-node solution for large and complex metagenomics assembly via succinct de Bruijn graph. *Bioinformatics* **31**, 1674–1676 (2015). 25609793 10.1093/bioinformatics/btv033

[90] Nissen, J. N. et al. Improved metagenome binning and assembly using deep variational autoencoders. *Nat. Biotechnol.* **39**, 555–560 (2021). 33398153 10.1038/s41587-020-00777-4

[91] Van Den Bossche, T. et al. Critical Assessment of MetaProteome Investigation (CAMPI): A multi-laboratory comparison of established workflows. *Nat. Commun.* **12**, 7305 (2021). 34911965 10.1038/s41467-021-27542-8 PMC8674281

[92] Shaffer, M. et al. DRAM for distilling microbial metabolism to automate the curation of microbiome function. *Nucleic Acids Res.* **48**, 8883–8900 (2020). 32766782 10.1093/nar/gkaa621 PMC7498326

[93] Buchfink, B., Reuter, K. & Drost, H. G. Sensitive protein alignments at tree-of-life scale using DIAMOND. *Nat. Methods* **18**, 366–368 (2021). 33828273 10.1038/s41592-021-01101-x PMC8026399

[94] Madeira, F. et al. The EMBL-EBI Job Dispatcher sequence analysis tools framework in 2024. *Nucleic Acids Res.* **52**, W521–W525 (2024). 38597606 10.1093/nar/gkae241 PMC11223882

[95] Wilkins, D. & Kurtz Z. gggenes: draw gene arrow maps in ‘ggplot2’. (2019)

[96] Emms, D. M. & Kelly, S. OrthoFinder: phylogenetic orthology inference for comparative genomics. *Genome Biol.* **20**, 238 (2019). 31727128 10.1186/s13059-019-1832-y PMC6857279

[97] Kay, M. ggdist: Visualizations of distributions and uncertainty in the grammar of graphics. *IEEE Trans. Vis. Comput Graph* **30**, 414–424 (2024). 37883271 10.1109/TVCG.2023.3327195

[98] Tiedemann F. gghalves: Compose half-half plots using your favourite geoms (2019).

[99] Slowikowski, K. et al. ggrepel: Automatically position non-overlapping text labels with ‘ggplot2’ (2018).

[100] Terrapon, N. et al. PULDB: The expanded database of Polysaccharide Utilization Loci. *Nucleic Acids Res.* **46**, D677–D683 (2017). 10.1093/nar/gkx1022 PMC5753385 29088389

[101] Edgar, R. C. MUSCLE: Multiple sequence alignment with high accuracy and high throughput. *Nucleic Acids Res.* **32**, 1792–1797 (2004). 15034147 10.1093/nar/gkh340 PMC390337

[102] Capella-Gutiérrez, S., Silla-Martínez, J. M. & Gabaldón, T. trimAl: A tool for automated alignment trimming in large-scale phylogenetic analyses. *Bioinformatics* **25**, 1972–1973 (2009). 19505945 10.1093/bioinformatics/btp348 PMC2712344

[103] Darriba, D. et al. ModelTest-NG: A new and scalable tool for the selection of DNA and protein evolutionary models. *Mol. Biol. Evolution* **37**, 291–294 (2019). 10.1093/molbev/msz189 PMC6984357 31432070

[104] Kozlov, A. M., Darriba, D., Flouri, T., Morel, B. & Stamatakis, A. RAxML-NG: A fast, scalable and user-friendly tool for maximum likelihood phylogenetic inference. *Bioinformatics* **35**, 4453–4455 (2019). 31070718 10.1093/bioinformatics/btz305 PMC6821337

[105] Duchemin, W. et al. RecPhyloXML: A format for reconciled gene trees. *Bioinformatics* **34**, 3646–3652 (2018). 29762653 10.1093/bioinformatics/bty389 PMC6198865

[106] Wessel, D. & Flugge, U. I. A method for the quantitative recovery of protein in dilute-solution in the presence of detergents and lipids. *Anal. Biochem* **138**, 141–143 (1984). 6731838 10.1016/0003-2697(84)90782-6

[107] Kong, A. T., Leprevost, F. V., Avtonomov, D. M., Mellacheruvu, D. & Nesvizhskii, A. I. MSFragger: Ultrafast and comprehensive peptide identification in mass spectrometry-based proteomics. *Nat. Methods* **14**, 513–520 (2017). 28394336 10.1038/nmeth.4256 PMC5409104

[108] Leprevost, F. D. et al. Philosopher: A versatile toolkit for shotgun proteomics data analysis. *Nat. Methods* **17**, 869–870 (2020). 32669682 10.1038/s41592-020-0912-y PMC7509848

[109] Andersen, T. O. et al. Metabolic influence of core ciliates within the rumen microbiome. * ISME J.* **17**, 1128–1140 (2023). 37169869 10.1038/s41396-023-01407-y PMC10284877

[110] Yu, F. C., Haynes, S. E. & Nesvizhskii, A. I. IonQuant enables accurate and sensitive label-free quantification with FDR-controlled Match-Between-Runs. *Mol. Cell Proteom.* **20**, 100077 (2021). 10.1016/j.mcpro.2021.100077 PMC8131922 33813065

[111] Tyanova, S. et al. The Perseus computational platform for comprehensive analysis of (prote)omics data. *Nat. Methods* **13**, 731–740 (2016). 27348712 10.1038/nmeth.3901

[112] Iwamoto, Y. et al. Purification and characterization of bifunctional alginate lyase from Alteromonas sp. strain no. 272 and its action on saturated oligomeric substrates. *Biosci., Biotechnol., Biochem.* **65**, 133–142 (2001). 11272816 10.1271/bbb.65.133

[113] Remoroza, C. et al. Combined HILIC-ELSD/ESI-MS(n) enables the separation, identification and quantification of sugar beet pectin derived oligomers. *Carbohydr. Polym.* **90**, 41–48 (2012). 24751008 10.1016/j.carbpol.2012.04.058

[114] Varki, A. et al. Symbol nomenclature for graphical representations of glycans. *Glycobiology* **25**, 1323–1324 (2015). 26543186 10.1093/glycob/cwv091 PMC4643639

