# Supplementary material for: Alginate foraging is conserved in geographically and taxonomically distinct ruminant microbiomes
Source: Nat Commun. 2026 Jul 16;17:6394. doi: 10.1038/s41467-026-72045-z (PMC13377030; doi:10.1038/s41467-026-72045-z)
Supplement: Supplementary file 1 — Supplementary information [file 41467_2026_72045_MOESM1_ESM.pdf]

Supplementary information

Alginate foraging is conserved in geographically and taxonomically distinct ruminant microbiomes

Jeffrey P. Tingley[1,2]*, Alessandra Ferrillo[3]*, Marissa L. King[1], Alemayehu Kidane[4], Barinder Bajwa[1], Xiaohui Xing[1], Tina Johannessen[3], Alexsander Lysberg[3], Liv Torunn Mydland[4], Margareth Øverland[4], Greta Reintjes[5], Anna Y. Shearer[1,2], Leeann Klassen[1], Kristin E. Low[1], Trushar R. Patel[2,6,7,8], Stephanie A. Terry[1], Phillip B. Pope[3,4,9], D. Wade Abbott[1,2†], and Live H. Hagen[3†]

*These authors contributed equally
†To whom correspondence should be addressed

CONTENT:

I. Supplementary Figures
II. Supplementary Tables
III. Supplementary Text

Metagenome-Assembled Genome (MAG) information and metaproteomics data are provided in Supplementary Data 1 and Supplementary Data 2, respectively.

Supplementary Figures

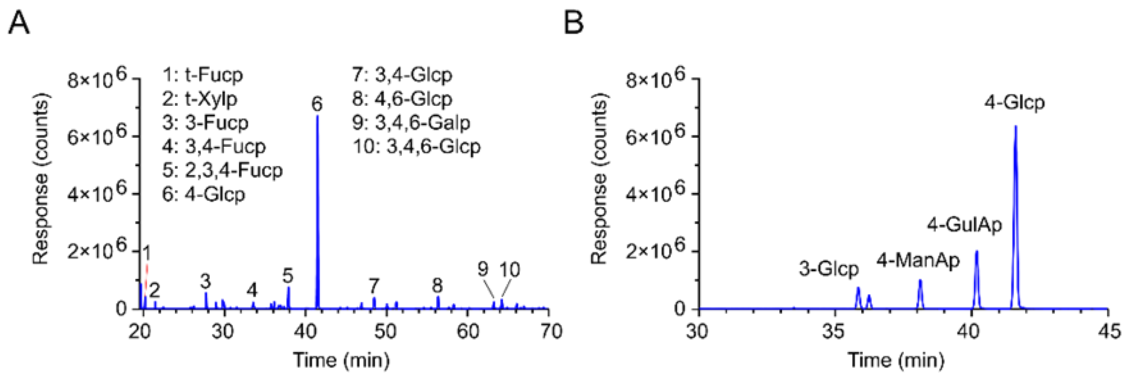

Supplementary Fig. 1: Linkage analysis chromatographs of *S. latissima*. Total ion current chromatograms of *S. latissima* with (A) standard methanolysis and (B) carboxyl reduced methanolysis with major PMMAs labelled.

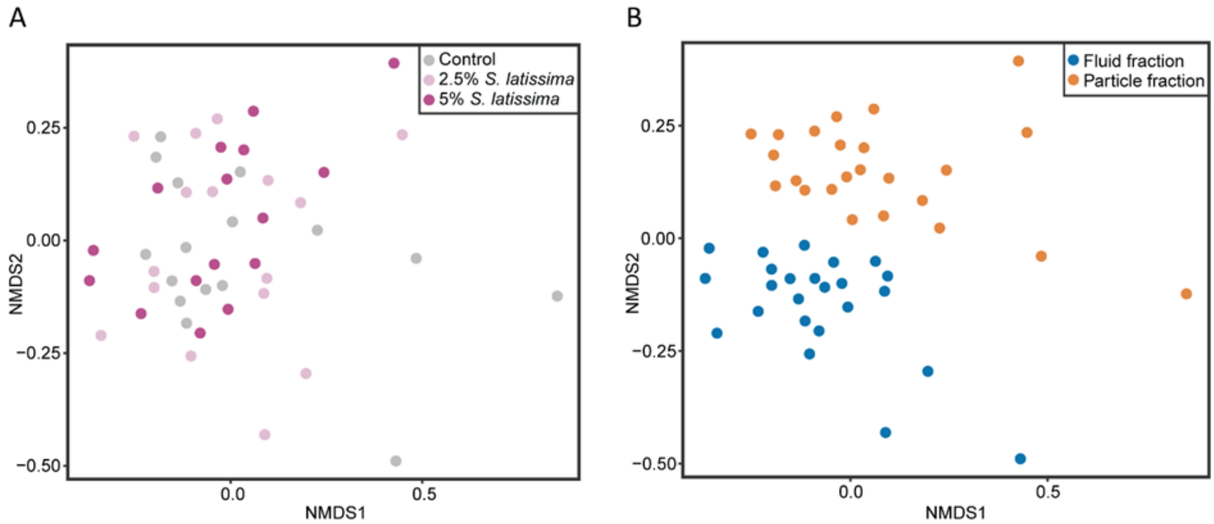

Supplementary Fig. 2: *In vivo* community analysis of lamb rumen microbial communities (16S rRNA gene) from dietary groups supplemented with 2.5% and 5% *S. latissima.* (A) Non-metric multi-dimensional scaling (NMDS) plot comparing the community composition in samples taken from control (grey), 2.5% (light purple) and 5% (dark purple) *S. latissima* dietary groups. B) The NMDS plot colored by sample fraction, either fluid (blue) or particle (orange).

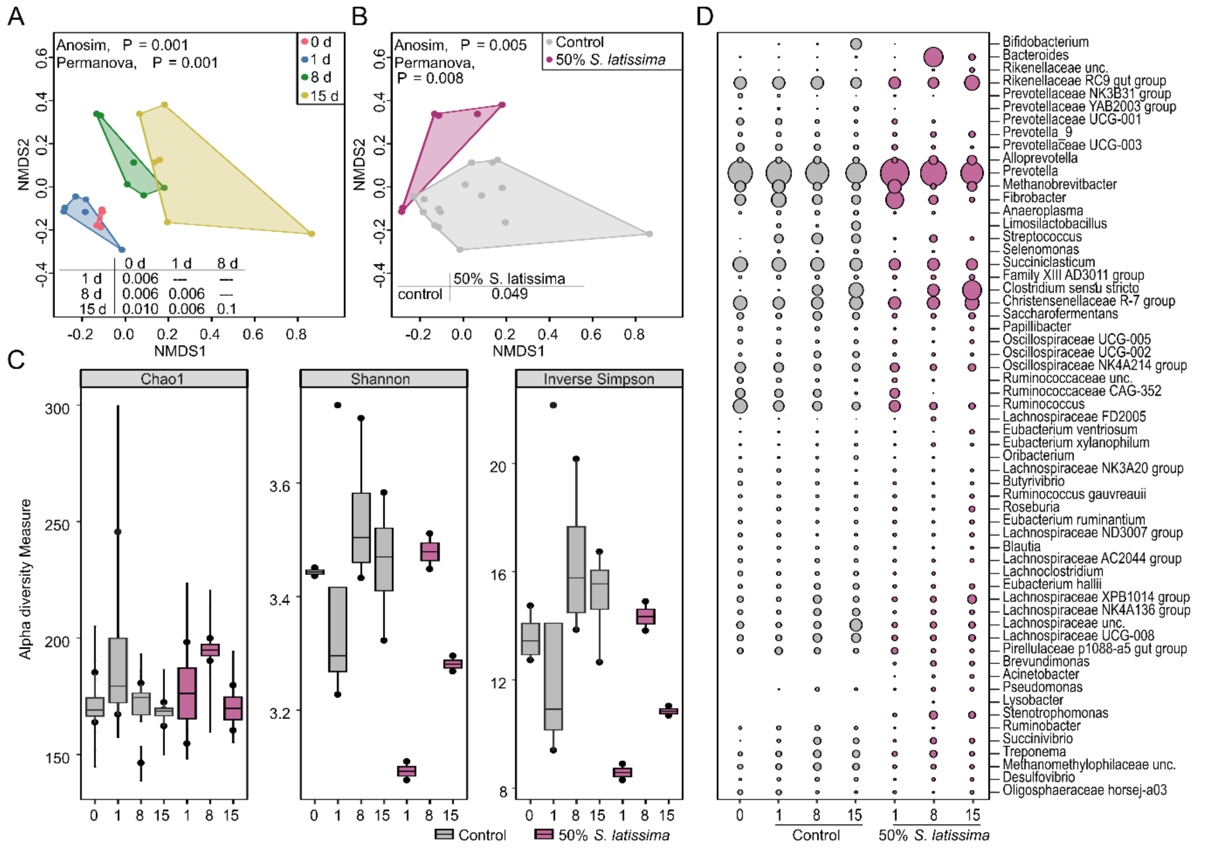

Supplementary Fig. 3: *Ex vivo* community analysis of RUSITEC rumen microbial communities (16S rRNA gene) supplemented with 50% *S. latissima.* A) Non-metric multi-dimensional scaling (NMDS) plot comparing the community composition at different time points: 0 d (pink), 1 d (blue), 8 d (green), and 15 d (yellow). B) NMDS plot comparing the effect each treatment has on the community composition where control (grey) and 50% *S. latissima* (purple). C) Alpha diversity (Chao1, Shannon, and inverse Simpson indices) in RUSITEC rumen microbial communities supplemented with 50% *S. latissima* over time. D) Bubble plot of different bacterial and archaeal genera that reached a minimum relative read abundance of 1% in control (grey) and 50% *S. latissima* (purple) RUSITEC rumen microbial communities.

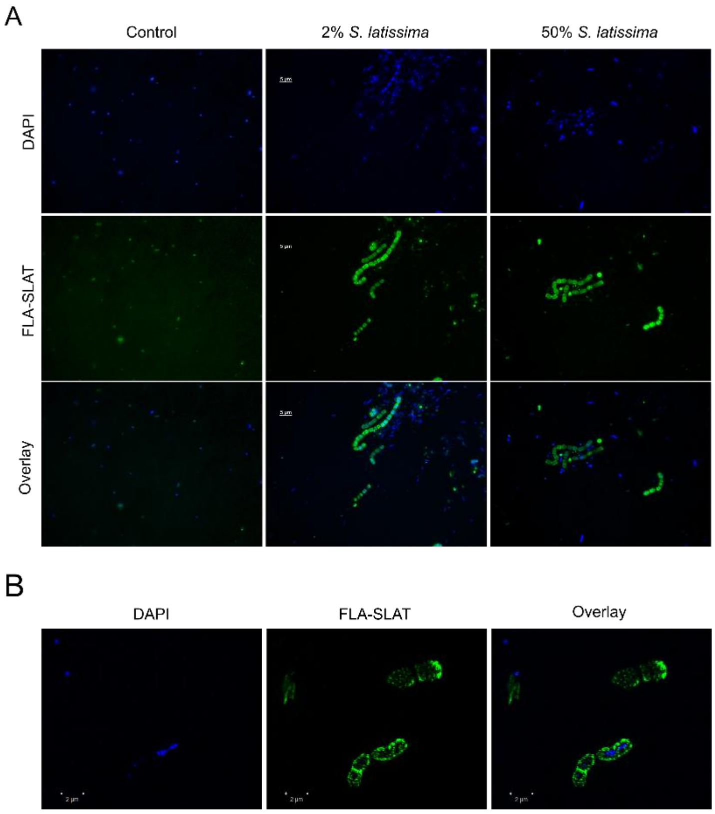

Supplementary Fig. 4: Epifluorescence visualization of FLA-SLAT interactions in RUSITEC rumen microbial communities that were enriched with 50% *S. latissima* for 15 days. A) FLA-SLAT interactions in control, 2% *S. latissima*, and 50% *S. latissima* RUSITEC microbial communities sampled from RUSITEC vessels after 15 days, stained with DAPI (blue) and incubated with 0.2 % FLA-SLAT (green) for 1 day. B) SR-SIM of cells from 50% *S. latissima* experiment after 1 day FLA-SLAT incubation. Cells were stained by DAPI, FLA-SLAT, and an overlay of DAPI and FLA-SLAT is shown.

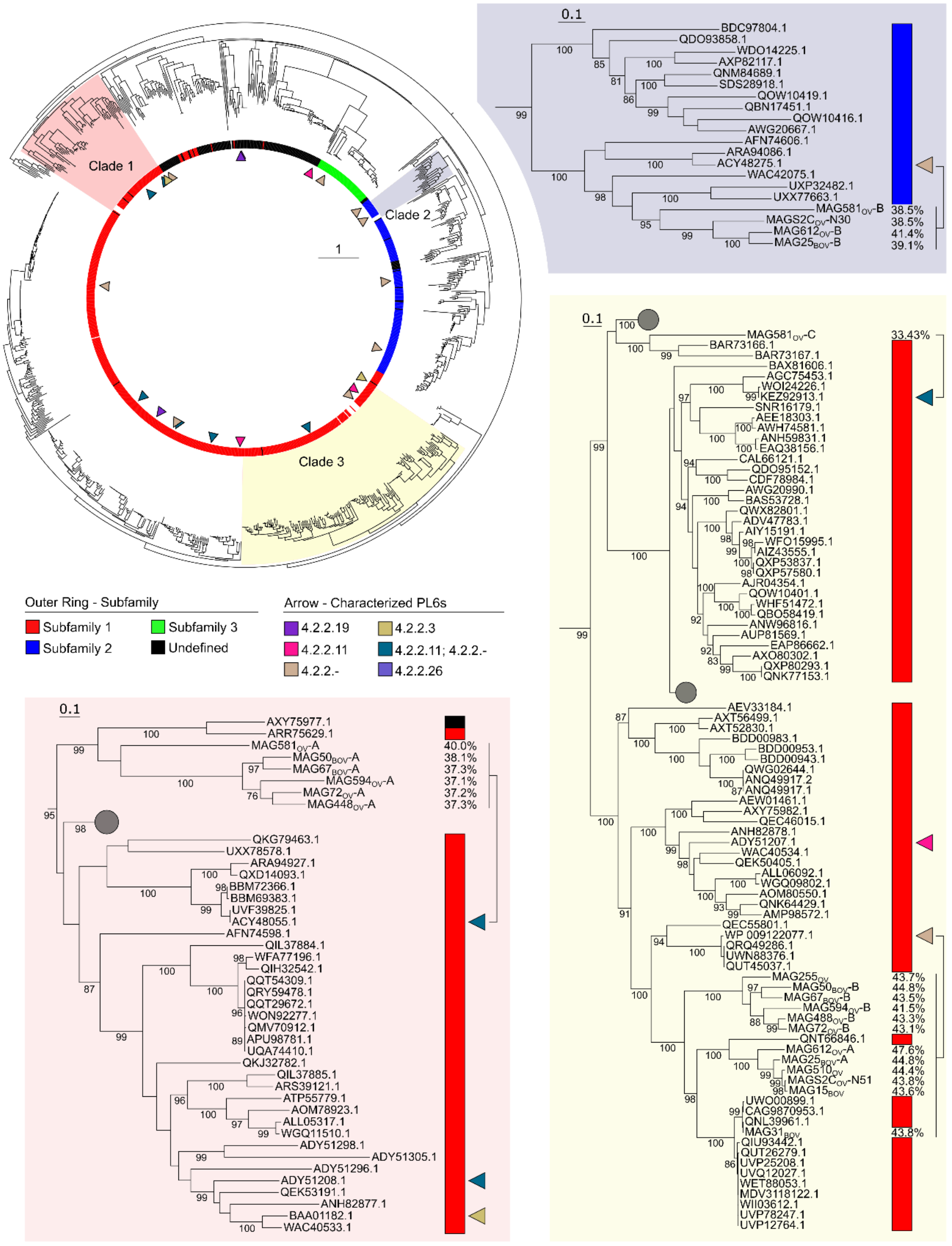

Supplementary Fig. 5: Similarity of *Bacteroidota* spp. PL6 members. SACCHARIS phylogeny of PL6 members within CAZy and *Bacteroidota* spp. members within this study. The full phylogeny (top left) highlights the three clades where *Bacteroidota* spp. PL6s were found (red – clade 1; blue – clade 2; yellow – clade 3). Clades were further pruned to identify closest related PL6 members (color highlighted as above), and the PL6 members within each clade was compared to the closest related characterized PL6 member. Clade 1 – ACY48055.1 (*Rhodothermus marinus* DSM 4252), clade 2 – ACY48275.1 (*Rhodothermus marinus* DSM 4252), and clade 3 - KEZ92913.1 (*Nonlabens ulvanivorans* PLR) and WP_009122077.1 (*Bacteroides clarus* YIT 12056).

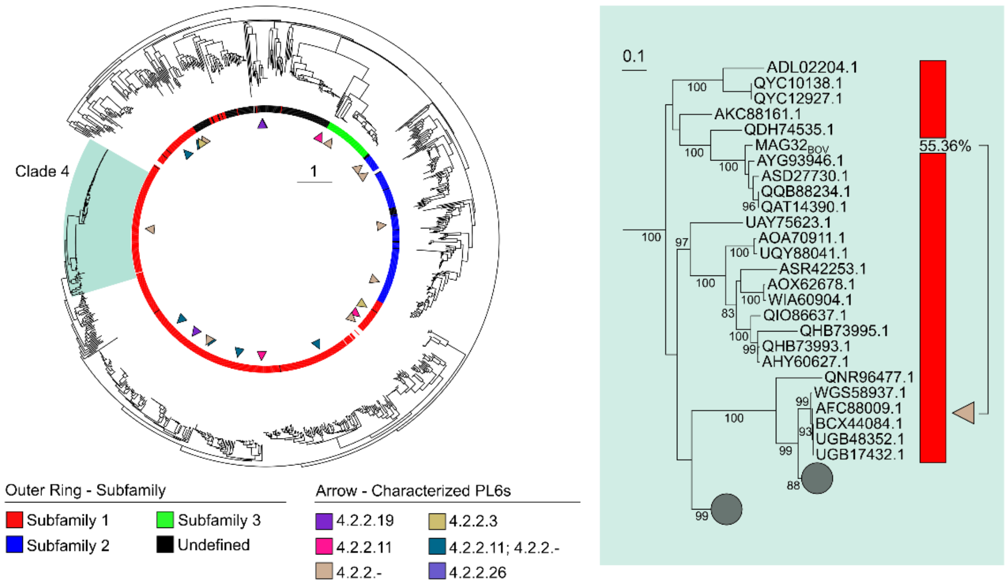

Supplementary Fig. 6: Similarity of *B. bullata* PL6 to other PL6 members. SACCHARIS phylogeny of PL6 members within CAZy and MAG32$_{BOV}$ PL6 member. The full phylogeny (left) highlights the MAG32$_{BOV}$ clade, which is further pruned (right) to display closest related PL6 members. The identity between MAG32$_{BOV}$ PL6 and the closest characterized PL6 member (AFC88009.1 - *Stenotrophomonas maltophilia* KJ-2) is displayed.

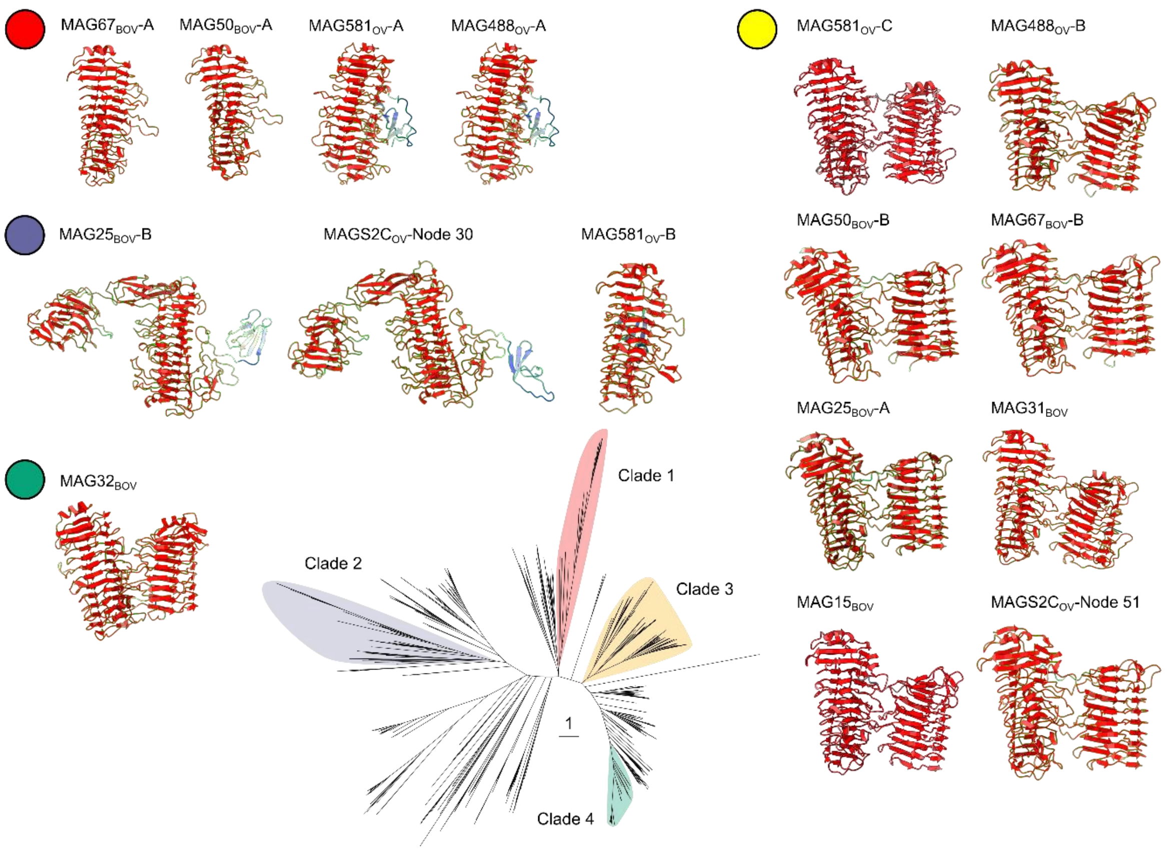

Supplementary Fig. 7: Predicted structure of alginate utilization loci (AUL) and alginate utilization cluster (AUC) PL6 members. AlphaFold [1] predicted structures of AUL and AUC PL6 members mapped to the PL6 phylogeny in Fig. 5**.** Structures are colored based on b factor (confidence). Structures are grouped by their corresponding clade.

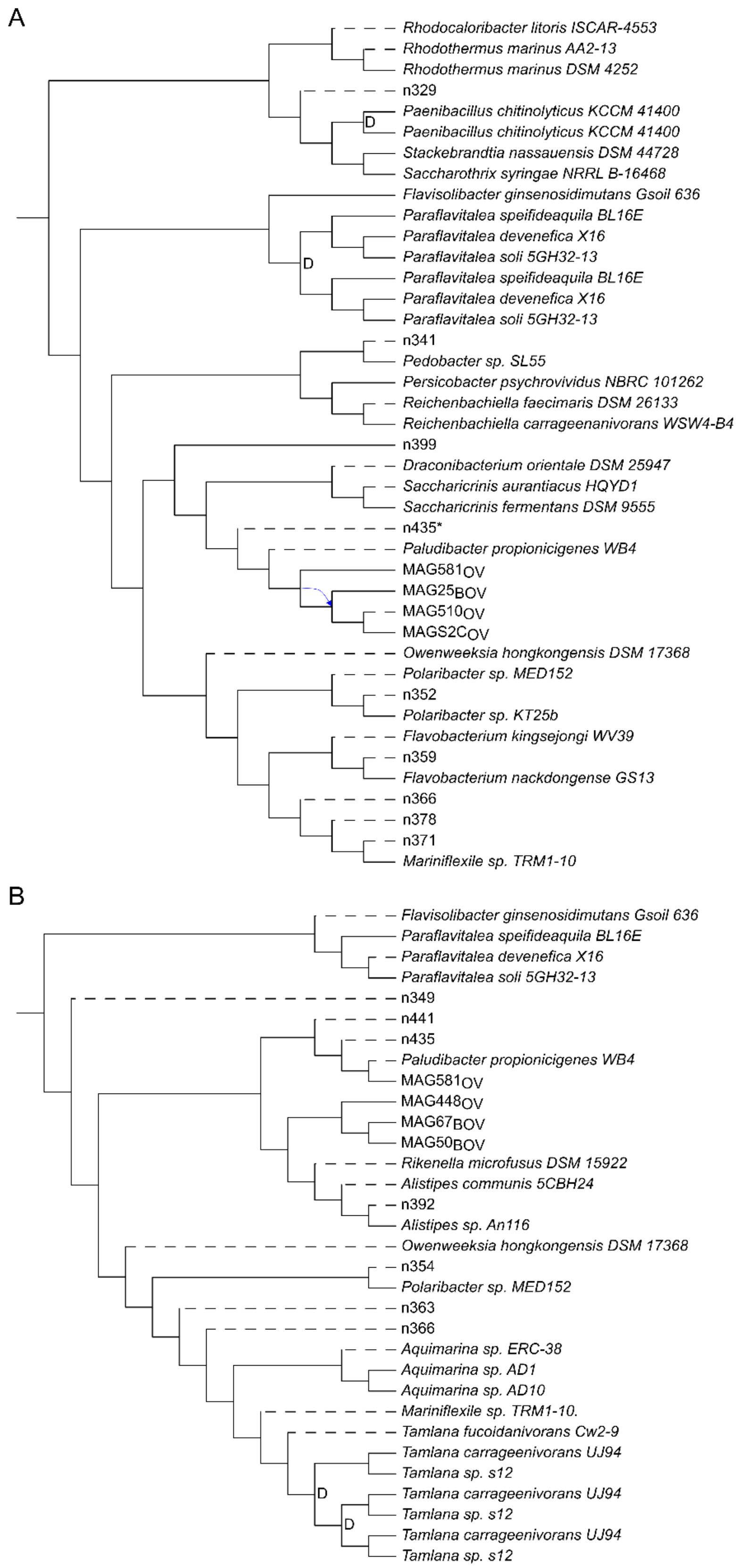

Supplementary Fig. 8: NOTUNG PL6 gene trees. PL6 Clade 2 (A) and Clade 1 (B) gene trees. Gene loss (dotted lines) and duplications "D" are marked per species and lines are drawn between clades represent predicted horizontal gene transfer events.

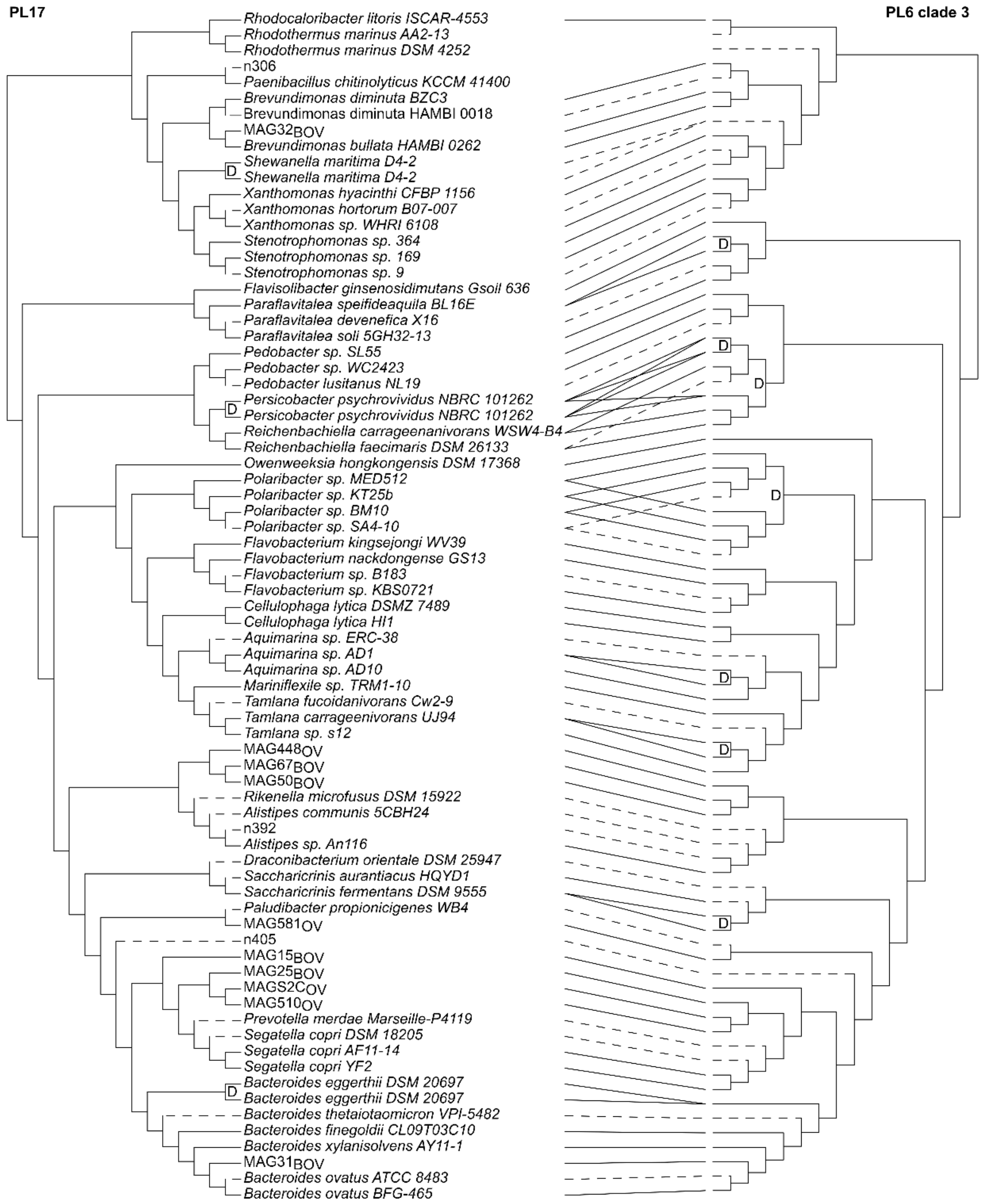

Supplementary Fig. 9: NOTUNG PL17 and PL6 Clade 3 gene trees. Comparison of PL17 and PL6 Clade 3 NOTUNG gene trees. Gene loss (dotted lines) and duplications “D” are marked per species and lines are drawn between trees to connect sequences within the same genome.

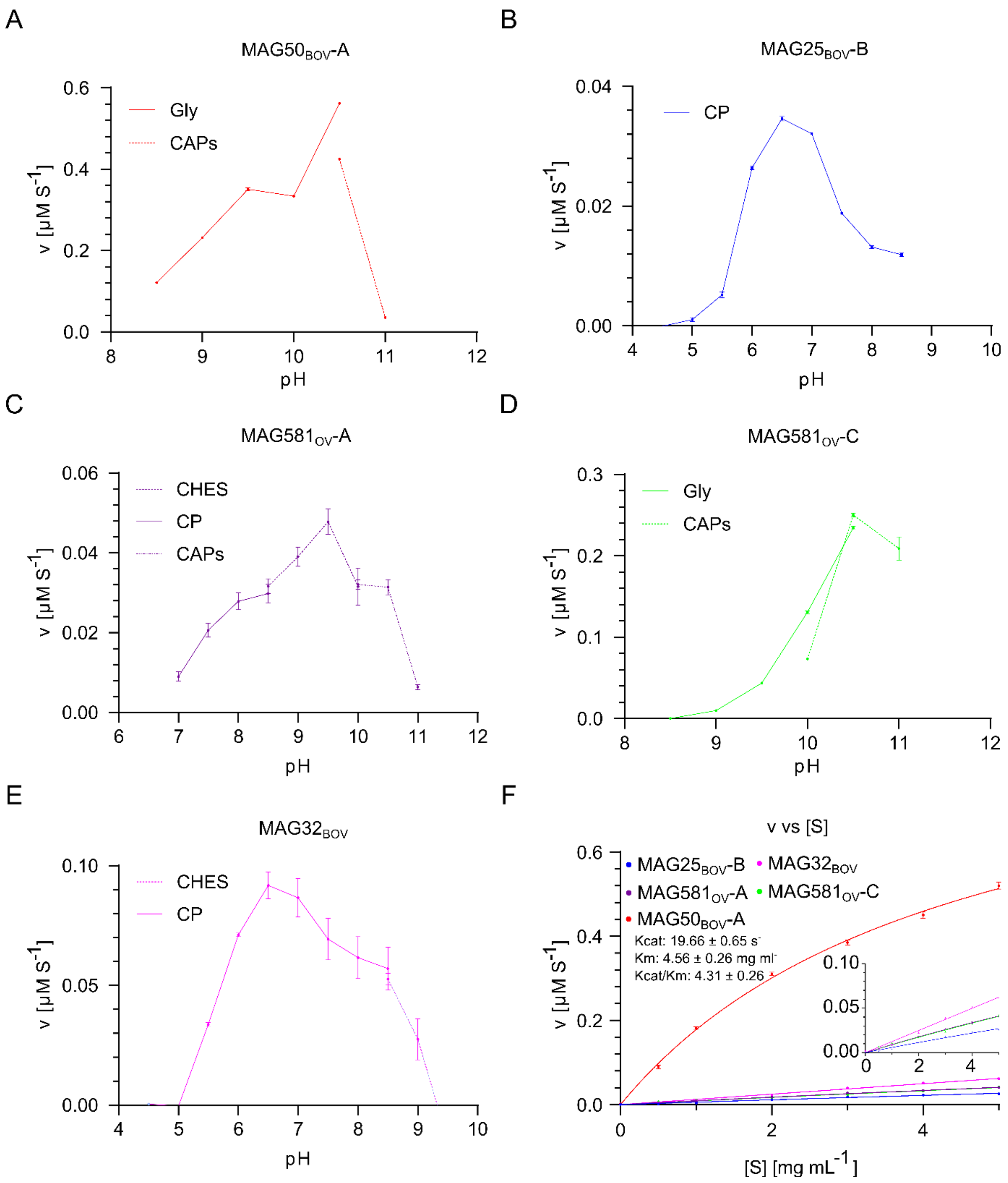

Supplementary Fig. 10: Activity of PL6 members of alginate types. A-E) pH optima of PL6 members within this study on brown seaweed alginate. Optima was determined using product formed (µM – calculated from absorbance at 232 nm) per second for 10-minute reactions. Buffers: Gly – Glycine, CAPs - 3-(Cyclohexylamino)propane-1-sulfonic acid, CP – Citrate-phosphate, CHES - N-cyclohexyl-2-aminoethanesulfonic acid. F) initial velocities of PL6 members within study on brown seaweed alginate. Velocities were determined using product formed (µM – calculated from absorbance at 232 nm) per second against substrate concentration (mg mL^{-1}) for 10-minute reactions. Enzyme catalytic efficiency was calculated for $MAG50_{BOV}$-A.

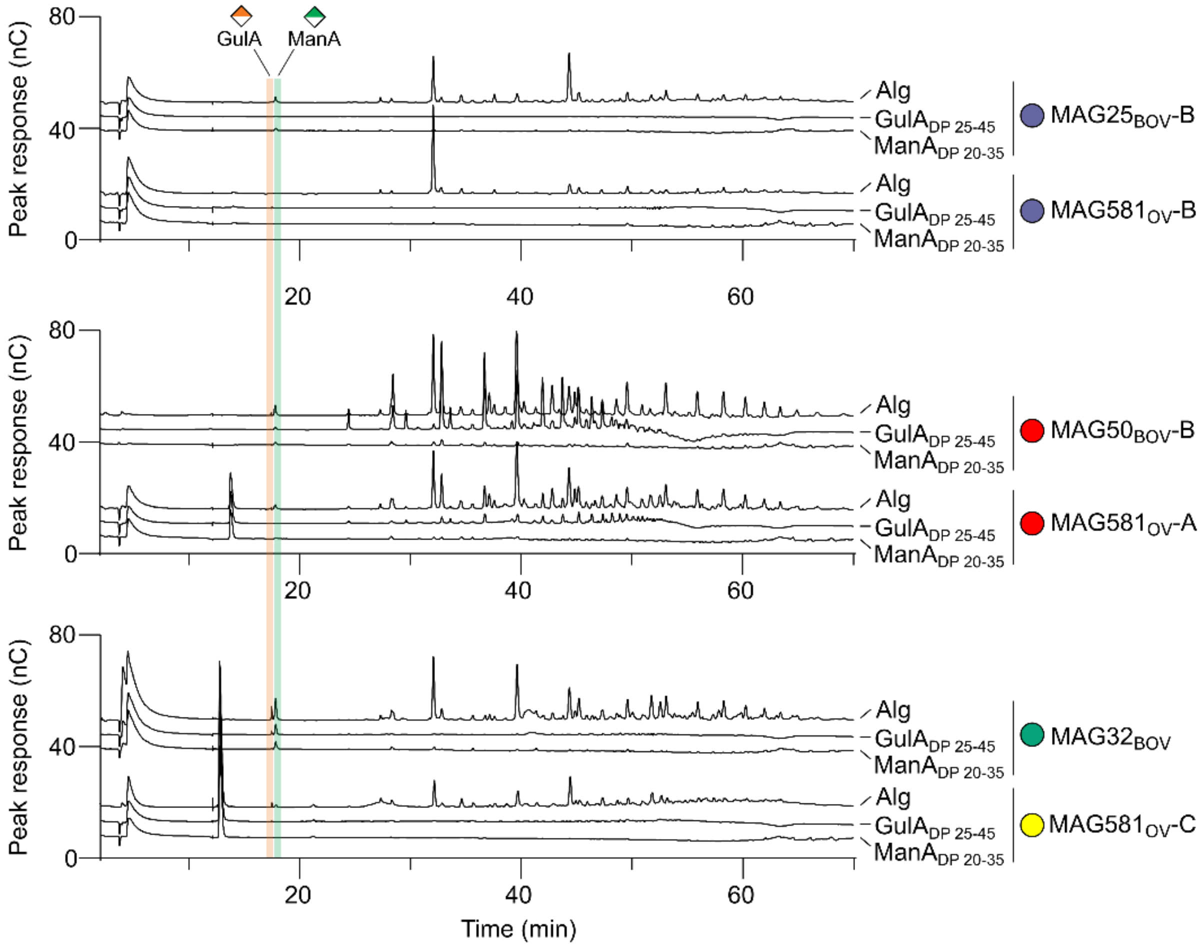

Supplementary Fig. 11: Characterization of PL6 members on alginate oligosaccharides. HPAEC-PAD product analysis of poly-GulA ($GulA_{DP 25-45}$) and poly-ManA ($ManA_{DP 20-35}$) digested with lamb and cattle-associated MAG PL6 enzymes. PL6 digests of alginate (Alg) not precipitated *via* EtOH were included as positive controls. Traces were separated into PL6 clade 2 (top), clade 1 (middle), and clade 3 and 4 (bottom) members. ManA and GulA represent monosaccharide standards.

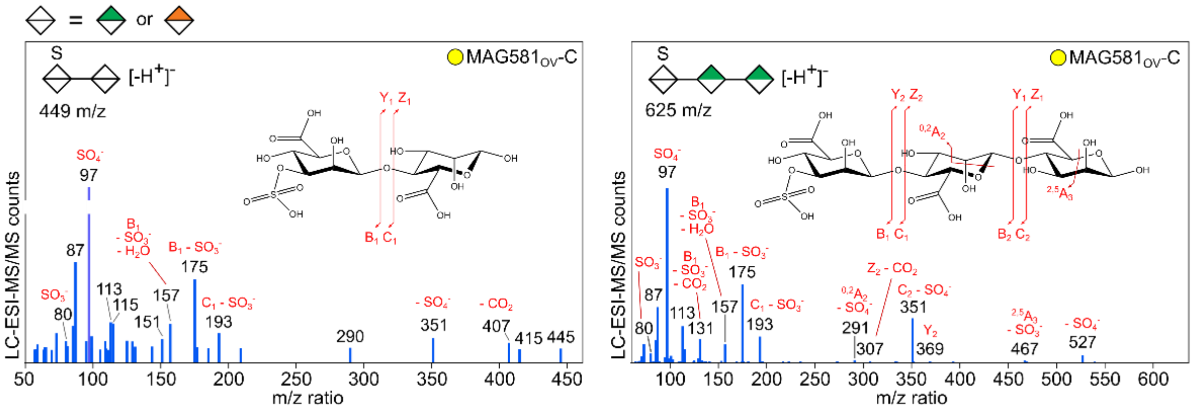

Supplementary Fig. 12: Sulfated alginate oligosaccharide enzyme products as identified by LC-ESI-MS/MS. Commercial alginic acid was incubated with MAG581-C enzyme and analyzed by LC-ESI-MS/MS. Oligosaccharide species for ions of *m/z* 449 and 625 were identified based on MS2 fragmentation data. Monosaccharide symbols are displayed according to the Symbol Nomenclature for Glycans system [2]. For monosaccharides unable to be discerned by LC-MS between mannuronate and guluronate, white symbols are used.

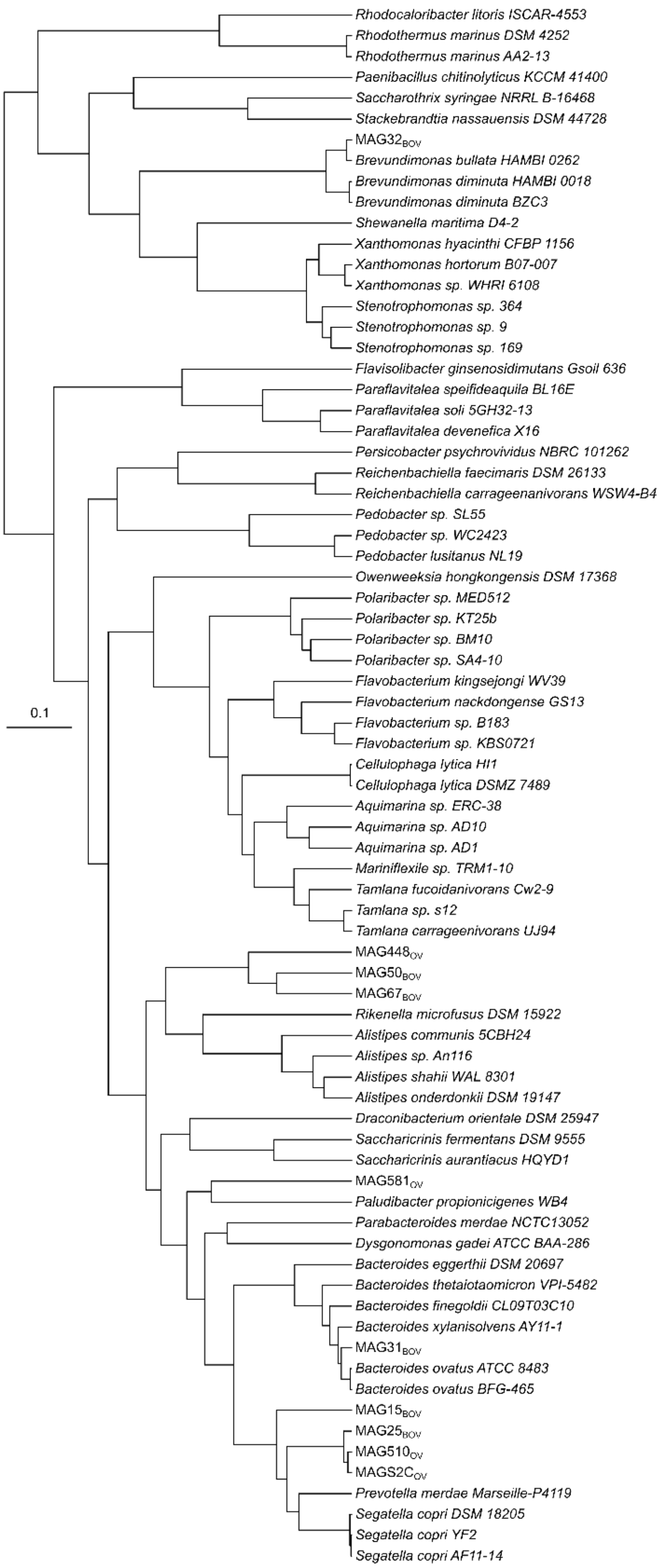

Supplementary Fig. 13: OrthoFinder generated species tree. The species tree was composed of AUL ruminant MAGs as well as NCBI genomes selected via their presence or absence (in the case of reference strains) of alginate lyases.

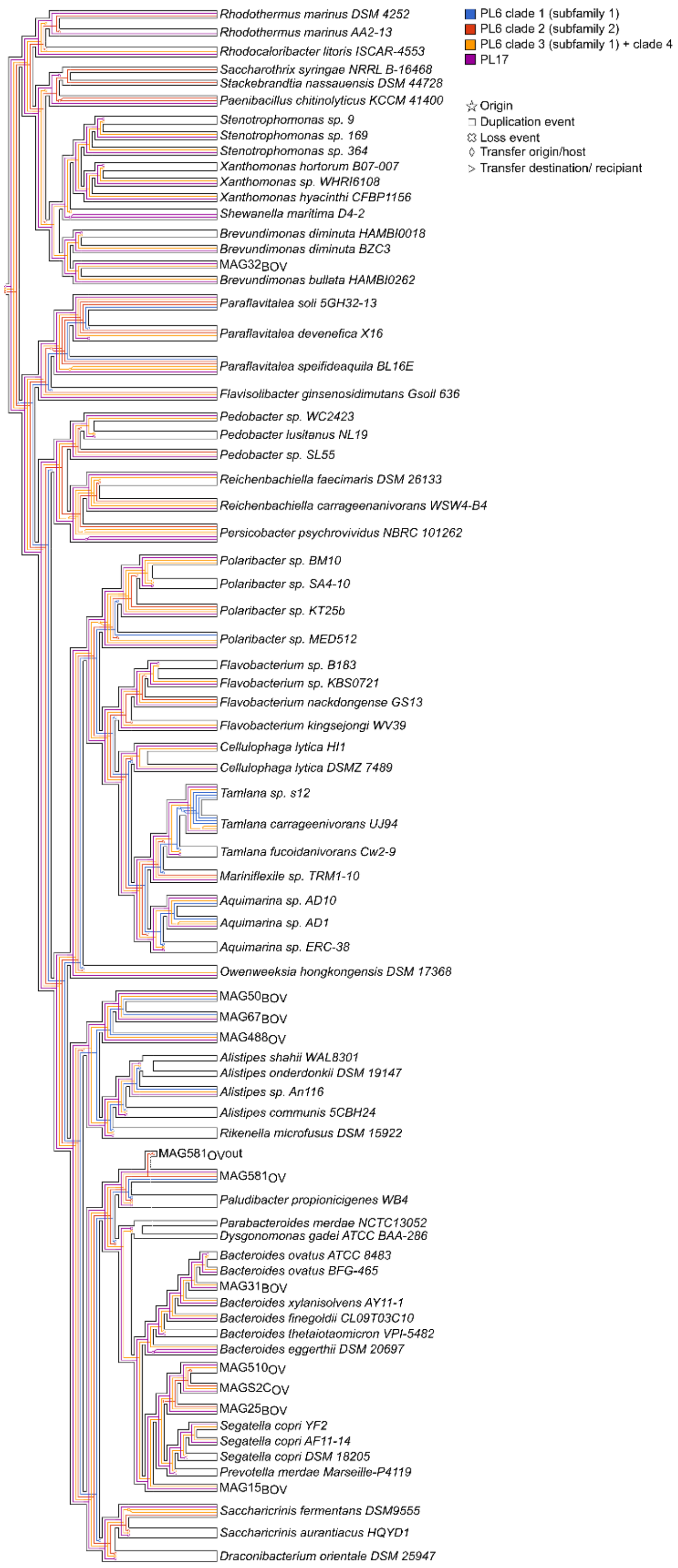

Supplementary Fig. 14: NOTUNG gene tree reconcilliations. All Notung gene trees were compiled and viewed via RecPhyloXML. Gene trees are represented via colored lines and the species tree is represented by the large white outline.

Supplementary Tables

Supplementary Table 1: Relative abundances (Mol%) of glycosidic linkages identified from the cell walls of *Saccharina latissima* via GC-MS (n=3).

PMAA	Avg. Mol %	PMAA	Avg. Mol %	PMAA	Avg. Mol %
3-Araf	Trace	4-Glcp	35.0±6.4	t-Rhap	Trace
5-Araf	Trace	6-Glcp	Trace	2-Rhap	Trace
t-Fucp	2.1±0.2	2,3-Glcp	Trace	3-Rhap	Trace
2-Fucp	1.2±0.1	3,4-Glcp	2.7±0.2	4-Rhap	Trace
3-Fucp	3.2±0.2	3,6-Glcp	Trace	2,3-Rhap	Trace
4-Fucp	1.7±0.4	4,6-Glcp	2.5±0.4	2,4-Rhap	Trace
2,3-Fucp	1.1±0.2	2,3,6-Glcp	Trace	3,4-Rhap	Trace
2,4-Fucp	0.8±0.1	2,4,6-Glcp	Trace	2,3,4-Rhap	Trace
3,4-Fucp	2.0±0.6	3,4,6-Glcp	2.6±0.8	t-Xylp	1.5±0.3
2,3,4-Fucp	5.1±1.0	2,3,4,6-Glcp	0.8±0.3	2-Xylp	Trace
t-Galp	Trace	t-Manp	Trace	3-Xylp	Trace
2-Galp	Trace	2-Manp	1.2±0.2	4-Xylp	Trace
4-Galp	Trace	3-Manp	Trace	2,4-Xylp	Trace
6-Galp	Trace	4-Manp	Trace	3,4-Xylp	Trace
3,4-Galp	1.4±0.2	2,3-Manp	Trace	2,3,4-Xylp	Trace
3,6-Galp	1.1±0.2	2,4-Manp	1.2±0.2	t-GalpA	Trace
4,6-Galp	Trace	2,6,Manp	Trace	2,4-GlcpA+2,4-GalpA	Trace
2,3,6-Galp	Trace	3,4-Manp	Trace	t-GlcpA	Trace
2,4,6-Galp	Trace	3,6-Manp	Trace	3-GlcpA	2.6±0.1
3,4,6-Galp	1.8±0.4	4,6-Manp	Trace	4-GlcpA	2.4±0.1
2,3,4,6-Galp	Trace	2,3,6-Manp	0.7±0.2	t-GulpA	Trace
2,4-Glcp+2,4-Galp	1.6±0.3	2,4,6-Manp	Trace	4-GulpA	10.1±1.8
t-Glcp	Trace	3,4,6-Manp	Trace	t-ManpA	Trace
3-Glcp	0.9±0.0	2,3,4,6-Manp	Trace	4-ManpA	4.6±0.6

Supplementary Table 2. The effect of 2.5% and 5% *S. latissima* on dry matter intake and apparent total tract digestibility of nutrients with Norwegian White lambs. Differences among the three treatment groups were assessed using one-way ANOVA (PROC ANOVA, SAS). This was a two-sided test, and if the global ANOVA was significant ($p < 0.05$), pairwise comparison were performed using Fisher's least significant difference (LSD) test to adjust for multiple comparisons.

	Control	2.5% *S. latissima*	5% *S. latissima*	SEM	P-value
DMI, kg/d	1.55	1.53	1.55	0.04	0.66
Digestibility (ATTD), %					
DM	81.1	80.0	78.9	2.63	0.27
OM	81.7	80.5	79.4	2.66	0.19
CP	83.5	81.6	79.6	2.57	0.012
NDF	78.5	77.7	76.2	3.13	0.36
Starch	99.3	99.1	99.0	0.28	0.13
Ash	74.4	74.2	74.0	2.56	0.96

DMI, Dry matter intake; ATTD, apparent total tract digestibility of nutrients, DM, dry matter; OM, organic matter; CP, crude protein; NDF, neutral detergent fiber.

Supplementary Table 3: The effect of 2.0% *S. latissima* on the degradability of nutrients and total volatile fatty acid production in the RUSITEC system. Differences among the two treatment groups were assessed using mixed-effect ANOVA (PROC MIXED, SAS) with treatment as a fixed effect, fermenter as a random effect and day of sampling as a repeated measure. This was a two-sided test, and significance was declared when $p < 0.05$. No adjustments for multiple comparisons was applied since only two groups were compared.

	Control	2% *S. latissima*	SEM	P-value
Degradability, %				
DM	57.1	58.4	0.80	0.0009
OM	58.0	57.3	1.67	0.45
CP	69.3	69.0	0.85	0.77
NDF	36.1	35.9	2.75	0.87
Total VFA, mmol	68.4	68.3	1.82	0.95

DM, dry matter; OM, organic matter; CP, crude protein; NDF, neutral detergent fiber; VFA, volatile fatty acids

Supplementary Table 4. Spearsman's rank correlation (two-sided) between relative abundance of lamb rumen MAGs encoding alginate lyases. Relative abundance values are based on CoverM output and include all samples (n=12) across the three dietary treatments (control, low and high seaweed dose). MAGs showing significant correlations (p < 001) are highlighted in the table and denoted with asterisks in Fig 2B.

MAG ID	rho	P-value	P-value adjusted (FDR)
$MAGS21C8_{ov}$	0.36	0.25	0.41
**$MAG510_{ov}$**	**0.95**	**0.00000195**	**0.0000195**
**$MAGS2C_{ov}$**	**0.89**	**0.00011**	**0.000548**
$MAG612_{ov}$	0.34	0.28	0.41
$MAG581_{ov}$	0.49	0.11	0.22
$MAG255_{ov}$	0.63	0.028	0.094
$MAG594_{ov}$	0.58	0.046	0.12
$MAG650_{ov}$	-0.25	0.43	0.47
$MAG448_{ov}$	0.30	0.35	0.44
$MAG72_{ov}$	-0.075	0.82	0.82

Supplementary Table 5: Species - gene tree reconciliation genomes.

GeneBank ID	Species name	GeneBank ID	Species name
GCA_006542665.1	*Alistipes communis* 5CBH24	GCA_040026395.1	*Pedobacter lusitanus* NL19
GCA_025145285.1	*Alistipes onderdonkii* DSM 19147	GCA_026625705.1	*Pedobacter* sp. SL55
GCA_025145845.1	*Alistipes shahii* WAL 8301	GCA_040822065.1	*Pedobacter* sp. WC2423
GCA_002161005.1	*Alistipes* sp. An116	GCA_036492425.1	*Persicobacter psychrovividus* NBRC 101262
GCA_003443695.1	*Aquimarina* sp. AD1	GCA_002005425.1	*Polaribacter* sp. BM10
GCA_003443715.1	*Aquimarina* sp. AD10	GCA_900105145.1	*Polaribacter* sp. KT25b
GCA_026222555.1	*Aquimarina* sp. ERC-38	GCA_000152945.2	*Polaribacter* sp. MED152
GCA_025146565.1	*Bacteroides eggerthii* DSM 20697	GCA_002163835.1	*Polaribacter* sp. SA4-10
GCA_040687835.1	*Bacteroides finegoldii* CL09T03C10	GCA_900290275.1	*Prevotella merdae Marseille*-P4119
GCA_001314995.1	*Bacteroides ovatus* ATCC 8483	GCA_025639805.1	*Reichenbachiella carrageenanivorans* WSW4-B4
GCA_024758985.1	*Bacteroides ovatus* BFG-465	GCA_900176375.1	*Reichenbachiella faecimaris* DSM 26133
GCA_022453665.1	*Bacteroides thetaiotaomicron* VPI-5482	GCA_011682235.2	*Rhodocaloribacter litoris* ISCAR-4553
GCA_029369765.1	*Bacteroides xylanisolvens* AY11-1	GCA_009936255.1	*Rhodothermus marinus* AA2-13
GCA_034424665.1	*Brevundimonas bullata* HAMBI 0262	GCA_000024845.1	*Rhodothermus marinus* DSM-4252
GCA_002205555.1	*Brevundimonas diminuta* BZC3	GCA_000427365.1	*Rikenella microfusus* DSM 15922
GCA_034424705.1	*Brevundimonas diminuta* HAMBI 0018	GCA_001660705.2	*Saccharicrinis aurantiacus* HQYD1
GCA_000190595.1	*Cellulophaga lytica* DSMZ 7489	GCA_000517085.1	*Saccharicrinis fermentans* DSM 9555
GCA_000750195.1	*Cellulophaga lytica* HI1	GCA_009498035.1	*Saccharothrix syringae* NRRL B-16468
GCA_900111425.1	*Draconibacterium orientale* DSM 25947	GCA_003465445.1	*Segatella copri* AF11-14
GCA_000213555.1	*Dysgonomonas gadei* ATCC BAA-286	GCA_020735445.1	*Segatella copri* DSM 18205
GCA_007970805.1	*Flavisolibacter ginsenosidimutans* Gsoil 636	GCA_015074785.1	*Segatella copri* YF2
GCA_003076475.1	*Flavobacterium kingsejongi* WV39	GCA_004295345.1	*Shewanella maritima* D4-2
GCA_004355225.1	*Flavobacterium nackdongense* GS13	GCA_000024545.1	*Stackebrandtia nassauensis* DSM 44728
GCF_023539155.1	*Flavobacterium* sp. B183	GCA_014621775.1	*Stenotrophomonas* sp. 169
GCF_002007065.3	*Flavobacterium* sp. KBS0721	GCA_009832905.1	*Stenotrophomonas* sp. 364
GCA_003425985.1	*Mariniflexile* sp. TRM1-10	GCA_031582865.1	*Stenotrophomonas* sp. 9
GCA_000236705.1	*Owenweeksia hongkongensis* DSM 17368	GCA_002893765.1	*Tamlana carrageenivorans* UJ94
GCA_004117095.1	*Paenibacillus chitinolyticus* KCCM 41400	GCA_045788535.1	*Tamlana fucoidanivorans* Cw2-9
GCA_000183135.1	*Paludibacter propionicigenes* WB4	GCA_016767215.1	*Tamlana* sp. s12
GCA_900445495.1	*Parabacteroides merdae* NCTC13052	GCA_002285515.1	*Xanthomonas hortorum* B07-007
GCA_011759375.1	*Paraflavitalea devenefica* X16	GCA_009769165.1	*Xanthomonas hyacinthi* CFBP 1156
GCA_003555545.1	*Paraflavitalea soli* 5GH32-13	GCA_040202385.2	*Xanthomonas* sp. WHRI 6108
GCA_032395985.1	*Paraflavitalea speifideaquila* BL16E		

Supplementary Table 6: NOTUNG event scores.

Gene	Event score	Duplication	Transfers	Losses
PL6 Clade 1	34.5	7	0	24
PL6 Clade 2	25	2	1	19
PL6 Clade unknown	19	2	0	16
PL17	25.5	3	0	21

Supplementary Table 7: Summary statistics for GC content of each gene within the MAGs, including sample size (n), median, the percentile values.

Genome	n	median	Lower 66 percentile	Upper 66 percentile	Lower 95 percentile	Upper 95 percentile
$MAG488_{OV}$	2220	57.72974	54.86443	61.86385	45.47009	65
$MAG510_{OV}$	1896	47.7842	44.3299	51.88518	37.60684	56.52428
$MAG581_{OV}$	1636	49.13295	45.39128	52.87731	39.09465	56.25431
$MAGS2C_{OV}$	2267	47.6874	44.1923	52.46637	34.73374	56.15165
$MAG15_{BOV}$	2389	51.00069	45.94257	55.07008	40.31642	60.27528
$MAG25_{BOV}$	2450	47.43751	43.66391	51.96409	34.11241	35.37508
$MAG31_{BOV}$	5267	42.50441	39.50617	46.71916	32.83264	49.91453
$MAG32_{BOV}$	3005	67.94872	65.2303	71.48594	60	74.4642
$MAG50_{BOV}$	1675	55.53265	52.63386	59.24242	45.73171	61.7062
$MAG67_{BOV}$	1869	58.23529	55.38462	61.7105	46.69029	64.0625

Supplementary Table 8: LC-MS Gradient conditions for separation of alginate oligosaccharides.

Time (min)	A (%)<br>10 mM ammonium formate<br>50 mM formic acid<br>80% acetonitrile<br>20% water	B (%)<br>10 mM ammonium formate<br>50 mM formic acid<br>20% acetonitrile<br>80% water
0	100	0
1	70	30
60	20	80
61	20	80
67	100	0
68	100	0
75	100	0

Supplementary Table 9: Parameters for ESI-MSn on the Orbitrap Fusion Tribrid.

Parameter (units)	Value
ESI	
Spray voltage: negative ion (V)	2500
Sheath Gas (Arb)	35
Aux Gas (Arb)	10
Sweep Gas (Arb)	1
Ion Transfer Tube Temp (°C)	325
Vaporizer Temp (°C)	250
**MS**	
Detector Type	Orbitrap
Orbitrap Resolution	120K
Mass Range	Normal
Scan Range (m/z)	150-2000
RF Lens (%)	50
**MS2**	
Collision Energy Type	Normalized
Isolation Mode	Quadrupole
Activation Type	HCD
Collision Energy Mode	Stepped
Collision Energies (%)	30,45,60,80
Detector Type	Orbitrap
Orbitrap Resolution	30K

Supplementary Table 10: List of abbreviations and acronyms.

Abbreviation / Acronym	Description
PL	Polysaccharide Lyase
DM	Dry Matter
PUL	Polysaccharide Utilization Loci
AUL	Alginate Utilization Loci
AUC	Alginate Utilization Cluster
RUSITEC	Rumen Simulation Technique
FLAPS	Fluorescently Labelled Polysaccharides
FLA-ALG	Fluorescent Alginate
FLA-SLAT	Fluorescent *S. latissima* Extract
DAPI	4’6-diamidino-2-phenylindole
MAG	Metagenomic Assembled Genome
PMAA	Permethylated Alditol Acetates
HWE	Hot Water Extraction
Sus(C/D)-like	Startch utilization system like
LC-MS	Liquid Chromatography Mass Spectrometry
ESI-MS	Electrospray ionization mass spectrometry
TLC	Thin-Layer Chromatography
HPAEC-PAD	High-Performance Anion-Exchange Chromatography with Pulsed Amperometric Detection
LFQ	Label-Free-Quantification
Linkages; n's indicate linkages followed by monosaccharide. Examples:	
4-Fucp	1,4-linked Fucose pyranose
t-Fucp	terminal Fucose pyranose
2,3-Fucp	2,3-linked Fucose pyranose
Fucp	Fucose pyranose
ManpA (M)	Mannuronic acid pyranose
GulpA (G)	Guluronic acid pyranose
Glcp	Glucose pyranose
Xylp	Xylose pyranose
Manp	Mannose pyranose
ΔHexA	Unsaturated uronic monosaccharide

Supplementary Text

Results

Text 1: Digestibility and degradability impacts of *S. latissima* in diets
Evaluating the digestibility of seaweed in animal diet is crucial for understanding its effect on the overall ruminal digestive activity and nutrient utilization. Thus, ruminal kelp digestion was assessed both in the *in vivo* lamb feeding and the *in vitro* bovine RUSITEC experiments.

For the lamb experiment, mean dry matter intake (DMI, kg/d) was not affected by the inclusion of *S. latissima* to diets. However, *S. latissima* inclusion at 5% (DM, basis) reduced apparent total tract digestibility of crude protein by 4.67% relative to the control diet (Supplementary Table 2). Digestibility of other tested dietary components were not significantly affected despite numerical differences. For the RUSITEC experiment, inclusion of 2% *S. latissima* increased the *in vitro* degradability of DM by 2.24%. There were no effects of *S. latissima* inclusion on organic matter, crude protein, neutral detergent fiber, or production of total volatile fatty acids (Supplementary Table 3).

Methods

Text 2: *S. latissima* distribution map
The distribution map for *S. latissima* (Fig. 1) was generated using R v4.2.2 [3] in RStudio (v2025.09.0) with the packages ggplot2 (v4.0.1) [4] and sf (v1.0-23) [5]. Global coastline data were downloaded from the Natural Earth dataset using the rnaturalearth (v1.1.0) [6] package. *S. latissima* occurrence records were retrieved from the Ocean Biodiversity Information System (OBIS) database (https://obis.org) [7] using the robis (v2.11.3) [8] package with the search query “Saccharina latissima”. These points were plotted as individual coordinates (in brown), alongside the locations of the *S. latissima* collection sites and animal trials from the current study. The final map was cropped to the Northern Hemisphere and adjusted in Inscape v1.4.2.

Text 3: Fluorescent Polysaccharides (FLAPS)
A hot water extraction (HWE) was performed on the dried, ball-milled *S. latissima* sample by incubating 1 g of sample in 40 mL distilled water for 8 h at 70 °C. Samples were centrifuged (3,000 × *g*, 10 min) and the supernatant was transferred to a new tube, and the hot water incubation was repeated twice more. Supernatants from the same sample were pooled and freeze-dried. The dried *S. latissima* HWE was de-starched by incubation with α-amylase (E-BLAAM, Megazyme) in 100 mM maleic acid buffer (pH 6.0) containing 100 mM NaCl and 3 mM $CaCl_2$ for 8 h at 70 °C, followed by an incubation with amyloglucosidase (E-AMGDPD, Megazyme) for 4 h at 50 °C. Resulting de-starched samples were extensively dialyzed (3,500 Da MWCO) against distilled water, before being freeze-dried. Fluorescently labelled *S. latissima* HWE (FLA-SLAT) was produced using a previously defined protocol [9], where the purified FLA-SLAT was freeze-dried, covered in aluminum foil and stably stored at -20 °C until further use. For FLA-SLAT incubations, 1 mL from each RUSITEC vessel was collected at the day 15 time point, and were centrifuged (5,000 × *g*, 10 min). Pellets were suspended in 1 mL phosphate buffered saline (PBS; pH 7.4) and washed twice with PBS before a final resuspension in 2 mL PBS. 50 µL of each resuspended RUSITEC microbial community sample was incubated with 50 µL of 0.4% FLA-SLAT for 1 day within an anaerobic chamber

(atmosphere: 85% N_2, 10% CO_2, 5% H_2, at 37 °C), where 40 µL aliquots were taken at the 1 h and 1 day time points. The 1 h and 1 day aliquots were immediately centrifuged (5,000 × *g*, 10 min), and the pellet was fixed with 4% (v/v) formaldehyde overnight at 4 °C. Fixed samples were centrifuged (5,000 × *g*, 10 min) and pellets were washed twice with PBS before being resuspended in 1 mL PBS and stored at 4 °C. Samples were diluted 1:10 and filtered onto a 25 mm, 0.2 µm pore size Isopore™ filter (Sigma, USA) using a gentle vacuum of <200 mbar. Dried filter pieces were counterstained with 4’6-diamidino-2-phenylindole (DAPI) and mounted on a glass microscope slide using a 4:1 mixture of Citiflour™ AFI mountant solution to Vectashield® vibrance antifade mounting medium (Vector Laboratories, USA). The fluorescence images were taken using an Echo Revolve R4K (Upright & Inverted Capability) microscope equipped with motorized LED fluorescence light, 5 MP CMOS monochrome camera, a Plan X Apo oil; 1,42 NA, Revolve, 60x oil immersion objective, with LED light cubes DAPI (EX: 385/30 EM: 450/50 DM: 425) and LED light cubes FITC (EX: 470/40 EM: 525/50 DM: 495). SR-SIM images for RUSITEC samples were visualized on a Zeiss ELYRA PS.1 (Carl Zeiss) using 561 and 488m lasers and BP 573-613, BP 502-538 and BP 420-480 + LP 750 optical filters. Z-stack images were taken with a Plan-Apochromat 63 Å~ /1.4 Oil objective and processed with the software ZEN2011 (Carl Zeiss). Images were exported to ACMEtool software (M. Seder, Technology GmbH, http://www.technobiology.ch and Max Planck Institute for Marine Microbiology, Bremen), where signals were evaluated according to Bennke, Reintjes [10]. Cell enumeration counts were plotted in GraphPad Prism v8.0.2 and displayed as relative abundance values.

Text 4: 16S rRNA gene sequencing

Taxonomic assigned ASV from the lamb rumen microbiome were combined with variant abundance table and processed using Phyloseq v1.46.0 [11]. Reads flagged as Eukaryota, Chloroplast or Mitochondria were removed before the sequences were normalized for downstream analysis. The beta diversity was investigated using a Non-metric Multidimensional scaling (NMDS) analysis based on Bray-Curtis dissimilarity in vegan [12].

RUSITEC community analysis, statistics, and plotting of the Bracken output files were performed using R v4.2.2 in R-studio v2022.02.3 [13] with the packages: phyloseq [11], ggplot2 [4], picante [14], rioja [15] and vegan [12]. Each sample was rarefied to 28,530 random reads using the “rarefy_even_depth(sample.size = 0.9*raremax)” function of phyloseq [11] prior to alpha- and beta-diversity analyses. NMDS analyses based on Bray-Curtis dissimilarity indices were performed using the “vegdist()” function of vegan to visualize the separation of RUSITEC microbial communities between the different treatments and time points. Stress was determined to be 0.097 with the “metaMDS()” function, and a Shepard diagram was used to determine the non-metric (R2= 0.991) and linear fit (R2= 0.968) between the observed dissimilarity. Statistical evaluation of the RUSITEC microbial communities was performed with an analysis of similarity (ANOSIM) using distance matrices [“anosim()” function, distance = bray, 999 permutations] and a permutational multivariate analysis of variance (PERMANOVA) using distance matrices [“vegdist()” function, method = bray, 999 permutations]. This analysis was followed by a pairwise comparison of the treatment or time point effect [pairwise.perm.manova()” function, method = Euclidian, “999 permutations] of RVAideMemoire [16]. Observed OTUs, Shannon, and inverse Simpson indices were calculated using the “estimate_richness()” function of phyloseq, as a measure of microbial community alpha-diversity. Changes in alpha-diversity were assessed in GraphPad Prism v8.0.2 using the Kruskal-Wallis test with a post-hoc Dunn’s multiple comparisons.

Text 5: Metaproteomics

After protein extraction, peptide samples were processed using a nano LC-MS/MS coupled to a timTOF Pro mass spectrometer (Bruker, Germany). The peptides were separated by an Aurora C18 reverse-phase (1.6 µm, 120 Å) 25 cm x 75 µm analytical column with an integrated emitter (IonOpticks, Melbourne, Australia). The temperature of the column was kept at 50 °C using the integrated oven. Equilibration of the column was performed before the samples were loaded (equilibration pressure 800 bar). The flow rate was set to 300 nl min^{-1} and the samples were separated using a solvent gradient from 2% to 25% solvent B over 70 minutes, and to 37% over 9 minutes. The solvent composition was then increased to 95% solvent B over 10 min and maintained at that level for an additional 10 min. In total, a run time of 99 min min was used for the separation of the peptides. Solvent A is 0.1% (v/v) formic acid in milliQ water, while solvent B is 0.1% (v/v) formic acid in LC-MS grade acetonitrile. The timsTOF Pro was run in positive ion data dependent acquisition PASEF mode with the control software Compass Hystar v5.1.8.1 and timsControl v1.1.19 68. The acquisition mass range was set to 100 – 1700 m z^{-1}. The TIMS settings were: 1 $K0^{-1}$ Start 0.85 V·s cm^{-2} and 1 $K0^{-1}$ End 1.4 V·s cm^{-2}, ramp time 100 ms, ramp rate 9.42 Hz, and duty cycle 100%. The capillary voltage was set at 1400 V, dry gas at 3.0 l min^{-1}, and dry temp at 180 °C. The MS/MS settings were the following: number of PASEF ramps 10, total cycle time 0.53 sec, charge range 0-5, scheduling target intensity 20,000, intensity threshold 2,500, active exclusion release after 0.4 min, and CID collision energy ranging from 27-45 eV.

Text 6: Recovery of virome scaffolds from lamb rumen

In addition to the prokaryote population, we attempted to recover viral content from the lamb metagenome co-assembly. This was done following the viral sequence identification SOP v3 (DOI:dx.doi.org/10.17504/protocols.io.bwm5pc86), with VirSorter2 v2.2.3 [17] and CheckV v0.8.1 [18]. DRAM-v.py was ran to annotate the sequences identified as viral, followed by manual curation according to the SOP. Annotated virome scaffolds are available in FigShare via https://doi.org/10.6084/m9.figshare.28925894. As the virome scaffolds were included in the sequence database for metaproteomics analysis, those with protein detection are provided in Supplementary Data 2. Further exploration of the rumen virome is beyond the scope of this study.

Supplementary References

1. Tunyasuvunakool K*, et al.* Highly accurate protein structure prediction for the human proteome. *Nature* **596**, 590-596 (2021).
2. Varki A*, et al.* Symbol nomenclature for graphical representations of glycans. *Glycobiology* **25**, 1323-1324 (2015).
3. R Core Team. R: A language and environment for statistical computing. R Foundation for Statistical Computing (2023).
4. Wickham H. ggplot2: Elegant gaphics for data analysis. Comprehensive R Archive Network (CRAN) (2016).
5. Pebesma E, Bivand R. Spatial Data Science: With Applications in R. Chapman and Hall/CRC (2023).
6. Massicotte P, South A. rnaturalearth: World Map Data from Natural Earth (2025).

7. OBIS. Ocean Biodiversity Information System. Intergovernmental Oceanographic Commission of UNESCO (2025).

8. Provoost P, Bosch S. robis: Ocean Biodiversity Information System (OBIS) Client (2020).

9. Reintjes G, Arnosti C, Fuchs BM, Amann R. An alternative polysaccharide uptake mechanism of marine bacteria. *The ISME Journal* **11**, 1640-1650 (2017).

10. Bennke CM*, et al.* Modification of a high-throughput automatic microbial cell enumeration system for shipboard analyses. *Appl Environ Microbiol* **82**, 3289-3296 (2016).

11. McMurdie PJ, Holmes S. phyloseq: An R package for reproducible interactive analysis and graphics of microbiome census data. *Plos One* **8**, (2013).

12. Oksanen JS*, et al.* Vegan: Community ecology package. version 0.1.4 edn. Comprehensive R Archive Network (CRAN) (2023).

13. RStudio Team. RStudio: Integrated development for R (v2022.02.3, Build 492). RStudio, PBC (2020).

14. Kembel SW*, et al.* Picante: R tools for integrating phylogenies and ecology. *Bioinformatics* **26**, 1463-1464 (2010).

15. Juggins S. rioja: Analysis of Quaternary Science Data. Comprehensive R Archive Network (CRAN) (2022).

16. Hervé M. RVAideMemoire: Testing and plotting procedures for biostatistics. Comprehensive R Archive Network (CRAN) (2023).

17. Guo J*, et al.* VirSorter2: A multi-classifier, expert-guided approach to detect diverse DNA and RNA viruses. *Microbiome* **9**, 37 (2021).

18. Nayfach S, Camargo AP, Schulz F, Eloe-Fadrosh E, Roux S, Kyrpides NC. CheckV assesses the quality and completeness of metagenome-assembled viral genomes. *Nat Biotechnol* **39**, 578-585 (2021).
