# Supplementary material for: Alginate foraging is conserved in geographically and taxonomically distinct ruminant microbiomes
Source: Nat Commun. 2026 Jul 16;17:6394. doi: 10.1038/s41467-026-72045-z (PMC13377030; doi:10.1038/s41467-026-72045-z)
Supplement: Supplementary file 2 — Description of Additional Supplementary Files [file 41467_2026_72045_MOESM2_ESM.pdf]

Description of Additional Supplementary Files

File name: **Supplementary Data 1**

Description: **Metagenome-Assembled Genome (MAG) information.** A) Lamb rumen MAG quality. B) Lamb rumen MAG taxonomic classification and relative abundance. C) Lamb rumen MAGs encoding genes annotated as alginate lyases. D) RUSITEC MAG quality. E) RUSITEC MAG taxonomic classification and relative abundance. F) RUSITEC MAGs encoding genes annotated as alginate lyases. G) Sequence similarity between alginate PUL genes found in lamb rumen and RUSITEC MAGs.

File name: **Supplementary Data 2**

Description: Metaproteomics data from the lamb feeding trial. A) All proteins detected (n= 10,312) from the lamb rumen microbiomes; control group and 5% S. latissima diet group. B) Detected proteins affiliated to *Prevotella* $MAG510_{ov}$. C) Detected proteins affiliated to *Prevotella* $MAGS2C_{ov}$. D) Organism-level normalization of alginate lyase PL6 detected within the Prevotella MAGs $MAG510_{ov}$ and $MAGS2C_{ov}$.
