# Supplementary material for: Alginate foraging is conserved in geographically and taxonomically distinct ruminant microbiomes
Source: Nat Commun. 2026 Jul 16;17:6394. doi: 10.1038/s41467-026-72045-z (PMC13377030; doi:10.1038/s41467-026-72045-z)
Supplement: Supplementary file 5 — Reporting Summary [file 41467_2026_72045_MOESM5_ESM.pdf]

nature portfolio

Corresponding author(s): Live H. Hagen and D. Wade Abbott

Last updated by author(s): Nov 12, 2025

Reporting Summary

Nature Portfolio wishes to improve the reproducibility of the work that we publish. This form provides structure for consistency and transparency in reporting. For further information on Nature Portfolio policies, see our Editorial Policies and the Editorial Policy Checklist.

Statistics

For all statistical analyses, confirm that the following items are present in the figure legend, table legend, main text, or Methods section.

n/a	Confirmed	
☐	☒	The exact sample size (*n*) for each experimental group/condition, given as a discrete number and unit of measurement
☐	☒	A statement on whether measurements were taken from distinct samples or whether the same sample was measured repeatedly
☐	☒	The statistical test(s) used AND whether they are one- or two-sided<br>*Only common tests should be described solely by name; describe more complex techniques in the Methods section.*
☒	☐	A description of all covariates tested
☐	☒	A description of any assumptions or corrections, such as tests of normality and adjustment for multiple comparisons
☐	☒	A full description of the statistical parameters including central tendency (e.g. means) or other basic estimates (e.g. regression coefficient) AND variation (e.g. standard deviation) or associated estimates of uncertainty (e.g. confidence intervals)
☐	☒	For null hypothesis testing, the test statistic (e.g. *F*, *t*, *r*) with confidence intervals, effect sizes, degrees of freedom and *P* value noted<br>*Give P values as exact values whenever suitable.*
☒	☐	For Bayesian analysis, information on the choice of priors and Markov chain Monte Carlo settings
☒	☐	For hierarchical and complex designs, identification of the appropriate level for tests and full reporting of outcomes
☐	☒	Estimates of effect sizes (e.g. Cohen's *d*, Pearson's *r*), indicating how they were calculated

Our web collection on statistics for biologists contains articles on many of the points above.

Software and code

Policy information about availability of computer code

Data collection	No software code used for data collection. Processing of raw omics data is described under 'Methods'
Data analysis	All software packages used are described in Methods section, and are publicly or commercially available. They include: DADA2 pipeline in R v4.3.2, SILVA Database release 138.1, Phyloseq v1.46.0, metaSPAdes v3.13.0, MetaWRAP v1.3.2, CONCOCT v1.1.0, Maxbin2 v2.2.6, and Metabat2 v2.12.1., dRep v3.2.2, CheckM v1.1.3, CheckV v0.8.1, CoverM v0.6.1, dbCAN v3.0.7, Bakta v1.9.3, GTDB-Tk v2.4.0, trimmomatic v0.36, MegaHIT v1.2.9, 9 87, VAMB v3.0.2, DRAM v1.2.4 (DRAM.py and DRAM-v.py), VirSorter2 v2.2.3, Diamond BLASTp v2.1.8, Clustal Omega, OrthoFinder v2.5.5, SACCHARIS 2.0 v2.0.0.dev19, MUSCLE v5.1, trimAI v1.5.0, ModelTest—NG v0.1.7, RAxML-NG v1.2.2, NOTUNG v3.0-beta, RecPhyloXML, FragPipe v16.3, MSFragger v3.3, Philospopher v4.0.0, Perseus v1.6.15.0, Xcalibur and Freestyle software packages, ZEN2011 and ACMEtool software, GraphPad Prism v8.0.2. R v4.2.2 in R-studio v2022.02.3 7 was used with the packages gggenes, gdist, gghalves, ggrepel, phyloseq, ggplot2, picante, rioja and vegan. The codes generated in this study are available at Zenodo, doi:10.5281/zenodo.14515212.

For manuscripts utilizing custom algorithms or software that are central to the research but not yet described in published literature, software must be made available to editors and reviewers. We strongly encourage code deposition in a community repository (e.g. GitHub). See the Nature Portfolio guidelines for submitting code & software for further information.

Data

Policy information about availability of data

All manuscripts must include a data availability statement. This statement should provide the following information, where applicable:

- Accession codes, unique identifiers, or web links for publicly available datasets
- A description of any restrictions on data availability
- For clinical datasets or third party data, please ensure that the statement adheres to our policy

Raw metagenomic sequencing data have been deposited to the Sequence Read Archive (SRA) under the project accession numbers PRJEB83690 (lamb in vivo feeding experiment) and PRJNA1200888 (bovine RUSITEC experiment). The metagenome-assembled genomes (MAGs) are publicly available via Figshare (lamb rumen MAGs doi: 10.6084/m9.figshare.28024343; bovine RUSITEC MAGs, doi: 10.6084/m9.figshare.28024394). The proteomics data, including the complete database, have been deposited in the ProteomeXchange Consortium (http://proteomecentral.proteomexchange.org) via the PRIDE partner repository with the dataset identifier PXD059090. All LC-MS data is deposited to the GlycoPost repository under the ID GPST000612. Source Data are provided with this paper.

Research involving human participants, their data, or biological material

Policy information about studies with human participants or human data. See also policy information about sex, gender (identity/presentation), and sexual orientation and race, ethnicity and racism.

Reporting on sex and gender	N/A
Reporting on race, ethnicity, or other socially relevant groupings	N/A
Population characteristics	N/A
Recruitment	N/A
Ethics oversight	N/A

Note that full information on the approval of the study protocol must also be provided in the manuscript.

Field-specific reporting

Please select the one below that is the best fit for your research. If you are not sure, read the appropriate sections before making your selection.

[x] Life sciences [ ] Behavioural & social sciences [ ] Ecological, evolutionary & environmental sciences

For a reference copy of the document with all sections, see nature.com/documents/nr-reporting-summary-flat.pdf

Life sciences study design

All studies must disclose on these points even when the disclosure is negative.

Sample size	For the in vivo trial, we used 24 lambs which allowed for 8 lambs per dietary group. Three beef heifers were recruited as rumen donors for the RUSITEC experiment. The exact number for samples used for the various analysis are indicated in the Material and Methods section. These samples sizes were chosen based on previous studies, and is considered sufficient to detect relevant responses while minimizing animal use.
Data exclusions	No data was excluded
Replication	The reported findings were detected in different host species (lamb and cattle), experimental set-up (in vivo feeding trial and ex vivo RUSITEC experiment) and on two different continents (Norway and Canada).
Randomization	For the in vivo feeding trail, 24 weaned ewe lambs with body weight 37.3 ± 1.6 kg were randomly assigned to three dietary treatment groups (n = 8 per group). All lambs had free access to clean drinking water and were fed the experimental diet ad libitum twice a day (at 08:00 h and 14:00 h) in individual pens. Samples from the the donor heifers used in the RUSITEC was pooled in equal proportions prior to the incubation.
Blinding	Researchers were not blinded during sample collection, but a randomized scheme was applied during omics sample preparation. Bioinformatic analysis was same for all diet groups, and processed together under identical conditions.

Reporting for specific materials, systems and methods

We require information from authors about some types of materials, experimental systems and methods used in many studies. Here, indicate whether each material, system or method listed is relevant to your study. If you are not sure if a list item applies to your research, read the appropriate section before selecting a response.

Materials & experimental systems

n/a	Involved in the study
☒	☐ Antibodies
☒	☐ Eukaryotic cell lines
☒	☐ Palaeontology and archaeology
☐	☒ Animals and other organisms
☒	☐ Clinical data
☒	☐ Dual use research of concern
☒	☐ Plants

Methods

n/a	Involved in the study
☒	☐ ChIP-seq
☒	☐ Flow cytometry
☒	☐ MRI-based neuroimaging

Animals and other research organisms

Policy information about studies involving animals; ARRIVE guidelines recommended for reporting animal research, and Sex and Gender in Research

Laboratory animals	A total of 24 lambs (Ovis aries) of the commercial meat breed Norwegian White (weanded ewe lambs) with an average age of 132 days at start of the experiment, and a body weight 37.3 ± 1.6 kg were used in the in vivo feeding experiment. For the ex vivo (RUSITEC) experiment, rumen inoculum was obtained from three ruminally cannulated Angus-cross heifers (Bos taurus) previously adapted to a barley silage and barley straw-based diet. The heifers were 16 months of age.
Wild animals	The study did not involve wild animals
Reporting on sex	The in vivo feeding trial was conducted on ewe lambs (Norwegian White), while the ex vivo RUSITEC experiment was based on beef heifers (Angus cross).
Field-collected samples	For the in vivo feeding trial, samples were collected at the end point of the experiment period (35 days). All animals were slaughtered at a commercial slaughterhouse (Rudshøgda, Nortura SA, Norway) and their intact gastrointestinal tracts were directly moved to a working bench. The stomach was opened, and the reticulo-rumen content was hand-mixed prior to sampling. The mixed sample was transferred into a sterile strainer blender bag (0.50 mm pore size; Stomacher® 400 Seward BA 6041, Worthing, UK) and gently squeezed to separate the fluid and particle phases. The phases were then sampled into cryotubes, transported in liquid nitrogen to the laboratory and stored at −80 °C until analysis.<br>For the ex vivo (RUSITEC) experiment, the rumen contents were collected from three ruminally cannulated beef heifers 2 h pre-feeding from four different sites within the rumen and squeezed through PECAP mesh (mesh size 250 µm; PA66CG-250 136 cm, Sefar Nytal, Gilbert Saguenay, QC, CA). Both the solid and liquid proportions were pooled in equal proportions from each heifer, and transported to the laboratory in an insulated thermos kept at 39 °C.
Ethics oversight	In vivo: All animal procedures were approved by the committee overseeing the rules and regulations governing animal experiments in Norway under the surveillance of the Norwegian Food Safety Authority (FOTS-ID: 16406).<br>Ex vivo: Donor heifers used in this experiment were cared for in accordance with the guidelines of the Canadian Council on Animal Care (2009) and were approved by the Institutional Animal Care and Use Committee (ACC2304).

Note that full information on the approval of the study protocol must also be provided in the manuscript.

Plants

Seed stocks	*Report on the source of all seed stocks or other plant material used. If applicable, state the seed stock centre and catalogue number. If plant specimens were collected from the field, describe the collection location, date and sampling procedures.*
Novel plant genotypes	*Describe the methods by which all novel plant genotypes were produced. This includes those generated by transgenic approaches, gene editing, chemical/radiation-based mutagenesis and hybridization. For transgenic lines, describe the transformation method, the number of independent lines analyzed and the generation upon which experiments were performed. For gene-edited lines, describe the editor used, the endogenous sequence targeted for editing, the targeting guide RNA sequence (if applicable) and how the editor was applied.*
Authentication	*Describe any authentication procedures for each seed stock used or novel genotype generated. Describe any experiments used to assess the effect of a mutation and, where applicable, how potential secondary effects (e.g. second site T-DNA insertions, mosiacism, off-target gene editing) were examined.*
