# Supplementary material for: Alginate foraging is conserved in geographically and taxonomically distinct ruminant microbiomes
Source: Nat Commun. 2026 Jul 16;17:6394. doi: 10.1038/s41467-026-72045-z (PMC13377030; doi:10.1038/s41467-026-72045-z)
Supplement: Supplementary file 6 — Transparent Peer Review file [file 41467_2026_72045_MOESM6_ESM.pdf]

nature portfolio

Peer Review File

Alginate foraging is conserved in geographically and taxonomically distinct ruminant microbiomes

Corresponding Author: Dr Live Hagen

This file contains all reviewer reports in order by version, followed by all author rebuttals in order by version.

Version 0:

Reviewer comments:

Reviewer #1

(Remarks to the Author)
The manuscript entitled "Microbial alginate foraging is conserved in geographically and taxonomically distinct ruminant microbiomes" by Ferrillo et al. investigated the digestion and utilization of brown algae, specifically Saccharina latissima, within the gut microbiome of different ruminant species. The study employed both in vivo and in vitro experimental systems to demonstrate how gut microbiota break down this algae using specific microbial catabolic pathways, emphasizing the significance of this process in carbon cycling and sustainable biomass resources. Overall the study is relevant, however, the following comments should be well addressed.

1. In the introduction, the authors mention how ruminant microbial communities interact with novel carbon sources but lack a detailed discussion on the diversity and functional characteristics of these microbial communities. It is recommended that this aspect be elaborated to strengthen subsequent arguments.
2. The paper does not fully consider the potential ecological impacts when discussing how ruminants adapt to novel carbon sources. The possibilities of niche competition and changes in microbial abundance with high proportions of algal feed are not adequately explored. It is advised to include this dimension.
3. The study identified alginate utilization loci (AULs) in several MAGs. However, the authors acknowledge that, "due to genome fragmentation, the susC/susD pair and the alginate lyases were found towards the edges on two separate contigs, and thus, a complete AUL could not be determined". This suggests that while the study identifies key components of AULs, it is limited by the incompleteness of genome reconstruction and cannot present a complete AUL for all identified bacteria. This could hinder a full understanding of the mechanisms of alginate utilization.
4. The study notes that not all PL6 enzymes demonstrated efficient digestion of alginate. While some PL6 members produced substantial amounts of unsaturated products, others showed variations in catalytic efficiencies and activity. This suggests that the study could benefit from further investigation into the precise activities of each PL6 enzyme. While the study mentions that the complexity of M/G ratio and the ability of PL6 enzymes to change mode of action are factors, more detailed characterization could be beneficial.
5. The metaproteomic analysis identified key proteins involved in alginate degradation, but it also mentions that other PL enzymes within AULs were not detected at the protein level. The study suggests some reasons for this, such as inclusion level, compartmentalized secretion, or insufficient adaptation time. However, further investigation into why these enzymes were not detected could be beneficial to enhance the understanding of the full metabolic response.

(Remarks on code availability)

Reviewer #2

(Remarks to the Author)
This manuscript addresses the question on seaweed catabolism in the rumen ecosystem of diverse livestock (lamb and cattle) and an 'artificial rumen' (RUSITEC). The authors provided orthogonal evidence (e.g., from multi-omics, physiology, biochemistry) that the ability to digest seaweed in diverse livestock around the globe likely is driven by alginate lyases with significant homology between different populations of mainly Bacteriodota. Thus, it seems that seaweed selects for specific members of microbiomes that are able to digest alginate regardless of the host species, which has implications not only for

our fundamental understanding but also for seaweed as a supplement to mitigate the emission of climate active gases produced by livestock. Although I think this result is exciting and impactful, the manuscript requires improvements to provide more evidence for some of the claims made.

Main suggestions:

The strongest claim, which sort of reflects the main outcome of this study, is that the environment selects in terms of host animals and their ability to digest seaweed. As soon as there is seaweed in the rumen of any livestock, bacteria of the genus Bacteriodota, which encode the necessary gene cluster for digesting seaweeds, will express those genes and therefore help the host animals regardless of their diversity and geographical location. Thus, alginate catabolism appears to be a core functional trait 'performed by autochthonous members of the ruminant microbiome'. As this ability appears to be widespread, the authors speculate that ancient horizontal gene transfer events (possibly from a marine bacterium) must have contributed to the ubiquitous presence of those gene clusters in diverse rumen microbiomes across species and geographical boarders. The evidence provided to support this claim is weak, though. The reasons are as follows. Neither suppl. figure S4 nor S5 provide sufficient evidence of HGT (e.g., insufficient description of origin of MAGs, unclear bootstrap support, unclear nested clades). Thus, I suggest to apply proper phylogenetic methods (e.g., NOTUNG; Reconciliation methods such as a comparison between gene and species trees) and/or parametric or alignment-based methods. Ideally, this work will include a set of marine bacteria associated with seaweed and for which the presence of these gene clusters is known. This comparison will help to start with disentangling the origin of those gene clusters. Divergence times can be estimated using coalescence theory. As this work is at the heart of this study, I suggest to show those results in the main paper and not the supplement.

The work nicely links the sequence diversity (end evolution) of genes, the structure of enzymes and their activity. However, I was missing a structural comparison between the key enzymes using FoldSeek. I am sure this will provide additional insights into how the evolution of genes is linked to the function and structure of their encoded enzymes.

I was was not able to find a proper synteny analysis although the authors claimed that this has been done. Figure 4a just shows the structure of the AULs but does not provide proper synteny information. There are many tools available for visualising syntenic relationships across multiple genomes such as https://academic.oup.com/bioinformatics/article/39/3/btad121/7072460 to only refer to one of them as an example. Any of those should be applied for synteny analyses.

Maybe I missed this (line 388), but would it be possible to try to estimate the relative abundance of those ruminal bacteria with the gene clusters for metabolising alginate? As 16S sequencing was done, this should be doable.

The abstract is a bit unusual as there is barely any information about the results and a brief discussion including concrete implications is missing, too. Hence, I suggest rewriting it.

Figure 3b is a bit too small considering its importance.

Depending on the outcome of the evolutionary analyses I have suggested above, the trees shown in the main manuscript likely need to be reconsidered. Maybe at least one of them can be moved to the supplement.

The doi:10.5281/zenodo.14515213 for looking at the code did not work for me. There was no entry at Zenodo under this DOI.

Last but not least, I was wondering about the novelty of this work as the authors referred to similar work several times in their manuscript. Thus, this needs to be made clear. Also elaborate more on the implications.

(Remarks on code availability)
The doi:10.5281/zenodo.14515213 for looking at the code did not work for me. There was no entry at Zenodo under this DOI.

Reviewer #3

(Remarks to the Author)
Ferrillo et al. study the ability and mechanisms of rumen microbiota to degrade non-terrestrial (alginate) polysaccharides. The authors found that several microbes from two different ruminants have the ability to degrade some of the polysaccharides derived from brown algae. The authors carefully analyzed the genes, their expression and for some also the specific degradation products. They also showed that some microbes bind alginate specifically using a fluorescence based assay. Overall this study provides new and important insights into the potential for the microbiota to provide ruminants with the ability to use forage that is not part of their typical diet. While I think this research will be an important contribution to

the body of knowledge on how the microbiota is involved in accessing different dietary components there are some concerns that would need to be addressed.

Major comments:

1. It is a bit surprising to me that microbes in the rumen have the ability to degrade alginate, which they usually do not have access to (except for the sheep which seem to be exposed to this regularly). I would expect that genes that are not used by these microbes would be lost or degrade over longer evolutionary times as they would represent an unnecessary physiological burden even if tightly regulated. Could these "alginate" degradation genes potentially be involved in the degradation of other polysaccharides that the rumen microbiota may "see" more frequently, which would explain their retention in their genomes. It would be great if the authors could speak to this "hypothesis" in their article particularly if the answer is "no". I do feel that the argument why these genes are retained is currently weak and it could be strengthened based on existing literature on glycan degradation pathways in gut microbiota across different animals.
2. The article has a LOT of abbreviations, many of which are not introduced, which makes it hard to read in some sections. While some of the glycan related abbreviations may be known to experts in that particular field a general reader such as myself really struggles with those. I would suggest to either write out terms, or provide a box in the article that gives an overview of the most used abbreviations.
3. The analysis of where the relevant genes and gene clusters have come from was a bit lacking. On page 21 there is a discussion that essentially concludes that these genes/gene clusters must have been in ruminant microbes for very long evolutionary times, but the evidence provided is weak. With your existing data you could do much more to investigate this question i.e. was it a recent gene transfer, are these genes very mobile etc. Generally I would actually expect high gene mobility based on the literature, that has shown that the glycan degradation gene clusters move around a lot by HGT. You could for example do more extensive phylogenetic analysis (and show them in more detail) of the relevant genes checking what free living and other gut microbes they have been found in, and if the 16S rRNA gene phylogenies are congruent with those gene phylogenies, which would support long term retention of the genes in those lineages, or alternative (my expectation) that the phylogenies of the genes and the 16S rRNA gene are not congruent which would indicate rampant horizontal gene transfer. Additionally, you can look at GC biases and GC skew to see if these cluster may be from recent integration events. Finally, you can map the reads from your metagenomes to the contigs and see if you have any obvious dips in read coverage, which would indicate heterogeneity in the relevant genome regions due to insertions and deletions.
4. The data in Figure 6B is a bit unexpected in that binding to the FLA-SLAT is increased even in controls after 1 day. It would be good to explain better what these observed patterns could mean. I would have for example expected much higher binding in the 50% samples. Also, is there any replication of this data? i.e. could differences be tested for statistically. Right now this data is kind of qualitative.
5. The analysis of the metaproteomic data in Figure 3 is a bit limited and is quite "cherry picked". I would be interested to know what some of the other very significant/different proteins are. I think understanding the response to the seaweed diet more holistically would be good. Adding gene names for more proteins to the volcano plot would be great and then at least one paragraph discussing some of the additional observed differences.
6. I wonder if it would make sense to add some framing regarding the hologenome theory of evolution to this manuscript. Essentially acquisition of microbes for rapid acquisition of new functions would argue for the hologenome theory, of course your current evidence may not support the theory too much, but once you look more about where these genes came from there may be a clearer story.

Minor comments:

1. The last sentence of the abstract (conclusion sentence) is vague. This could be more specific and stronger.
2. Graphical abstract is great, very clear.
3. Introduction is very clear.
4. L. 98, delete "high" or rewrite to similarity or identity. Homology is an absolute and there is not levels of homology.
5. L. 104, replace "their" with "kelp"
6. L. 104 to 108, long sentence in passive voice, split the sentence and rewrite to active voice.
7. L. 108 to 110, unclear to me what these compounds are, add some simple explanations for broader audience
8. Page 6, too many not explained abbreviations
9. L. 152, Shannan should be Shannon, also explain what the reader is supposed to learn from the comparison of these indices.
10. L. 157, whose "metabolic metrics", the sheep? For the reactors I would not know what this means. Please explain better.
11. L. 173, "We therefore extended…" I do not understand what this refers to. How did the refinement work? Did you look at the neighboring genes of the genes of interest?
12. L. 179 – 186 this paragraph is much clearer in its writing, maybe you can model some of the previous paragraphs on it.
13. L. 200, since you specify that they were not detected in "fiber attached rumen samples", were they detected in the non-fiber associated fraction?
14. Line, 205, delete "After filtering,"
15. Line 206, add "the" before detection
16. L. 210, add "a" before "dose-dependent"
17. L. 215, so how similar are the two respective MAGs in terms of ANI and how does this relate to the PL6 similarity?
18. L. 216, What do you mean by "functional redundancy"? some more specific explanation may be needed.
19. L. 230, fragmentation is usually due to strain variability in the population that you bin into one MAG. You can re-assemble these MAGs after read mapping to them, and then use linkage graph analysis for example with Bandage to see what fragments are linked together, this will allow you to deduce if all AUL genes are in one cluster. i.e. the linkage analysis shows you which contigs are neighboring.
20. Figure 3, I am wondering if you normalized the protein abundance data to each organism? If not some of the differences could be caused by differences in overall organism abundance and not in differences in within organism protein abundances i.e. compositionality of the data could be an issue here. You may want to double check that your results are the same for organism normalized data.

21. L. 253, replace homology with similarity, again, homology does not have levels
22. L. 254, delete “homology”
23. Figure 4B, Why do some of the arrow head not have numbers? Why is there no “green” square in the legend? The subfamily, clade terms being different (?) is confusing
24. Line 261, replace “metagenomics” with “metaproteomics”
25. Line 260, something is missing in that sentence, it is incomplete
26. Line 260, insert “The representations of AULs…”
27. Line 270, “amino acid homology” is incorrect, see comments above
28. Line 278, no clue what poly-MG is
29. Line 280, “exo-type” not sure if this is a scientific term
30. Line 284, endo-poly-MG???
31. Figure 5A, What is GulA in Gel picture, and GulA, ManA and Alg. In chromatogram, add to caption? What was the negative control in the gel, please describe in caption.
32. Figure 5 B & C, can you also show the negative controls for these measurements to show that these are specific mass peaks
33. Line 334, remove the “-“ between 10 and 7, this would be a terribly small number otherwise
34. Line 337 and 338, I do not understand what is referred to here. Needs to be written more clearly.
35. Line 339, what does it mean that the control also takes up the SLAT
36. Line 344/345, How do you explain this observation, it is counterintuitive, so it would be good so say something about it in way of explanation.
37. L. 338, do you really mean central role in general, or specifically a central role in alginate degradation.
38. L. 391, delete “protein”, proteins are not expressed, genes are expressed and the product of gene expression are proteins.
39. L. 392, No idea what “inclusion level” refers to, also no idea about “specificity of induction products”
40. L. 450, non-sequitur between the two sentences, make sure to add a transition that links the sentences
41. L. 454-476, rewrite based on major comment, currently the argument is not very clear.
42. L. 477, very vague ending of the sentence, maybe say something more specific about what the clues are and what we learn from it, right now I have to guess.
43. L. 488, the argument for the “be native to the ruminant microbiome” was not well presented above
44. L. 489-491, vague, be more specific about what these hypotheses are
45. L. 674, change > to <
46. L. 698, Since no details about peptide preparation are given either provide more text here, or provide an open access reference, or put it into the supplemental materials.
47. L. 713, Please add some information of how the data was normalized
48. L. 720, add all parameters for the imputation sampling
49. L. 722, It sounds as if you did not apply a correction for multiple hypothesis testing? Please do double check that things remain significant when you correct for multiple hypothesis testing using either the permutation based FDR or the Benjamini Hochberg FDR. Generally it would be better to report FDRs or q-values rather than p-values, which are not as meaningful in high-dimensional omics data.
50. L. 822, change “is” to “are”

Signed: Manuel Kleiner

(Remarks on code availability)

Version 1:

Reviewer comments:

Reviewer #1

(Remarks to the Author)
The manuscript shows substantial improvement, but several critical issues require further attention:
1.We acknowledge the inclusion of NOTUNG reconciliation and GC-content analysis addressing HGT concerns. However, the failure to perform codon usage bias analysis and exclusion of marine Bacteroidota genomes renders the vertical inheritance claim speculative. Provide CAI/ΔENc’ metrics and integrate marine lineages (e.g., Zobellia) to conclusively reject ancient HGT.
2.The supplemental enzyme data (poly-M/poly-G activity) is noted. Nevertheless, the absence of kinetic constants (kcat/Kmkcat/Km) for all PL6-substrate combinations and undefined activity thresholds invalidates functional conclusions. Tabulate full Michaelis-Menten parameters and adopt CAZy efficiency benchmarks immediately.
3.Claims of "compartmentalized secretion" or "extraction inefficiency" require empirical validation via spike-in recovery experiments. Quantify losses of commercial alginate lyases (e.g., Sphingomonas AlyA) in rumen matrices—not theoretical speculation.
4.Attributing reduced Shannon diversity to "technical factors" without co-occurrence networks or SCFA profiling is unacceptable. Correlate Prevotella dominance with taxon displacement and measure metabolic outputs to prove functional resilience.
5."Dose-dependent" MAG claims lack multiplicity-corrected p-values and effect sizes. Publicly archive raw data with full statistical documentation—retract any unsupported inference.

(Remarks on code availability)

Reviewer #2

(Remarks to the Author)
Many thanks for addressing all of my suggestions. I think the manuscript has significantly improved and the results are very robust now. Congratulations!

(Remarks on code availability)

Reviewer #3

(Remarks to the Author)
Thank you for doing comprehensive revisions and addressing my comments in detail. Manuscript is greatly improved. Great job!

Manuel Kleiner

(Remarks on code availability)

Open Access This Peer Review File is licensed under a Creative Commons Attribution 4.0 International License, which permits use, sharing, adaptation, distribution and reproduction in any medium or format, as long as you give appropriate credit to the original author(s) and the source, provide a link to the Creative Commons license, and indicate if changes were made.
In cases where reviewers are anonymous, credit should be given to 'Anonymous Referee' and the source.
The images or other third party material in this Peer Review File are included in the article's Creative Commons license, unless indicated otherwise in a credit line to the material. If material is not included in the article's Creative Commons license and your intended use is not permitted by statutory regulation or exceeds the permitted use, you will need to obtain permission directly from the copyright holder.
To view a copy of this license, visit https://creativecommons.org/licenses/by/4.0/

Response to Reviewer Comments

We greatly appreciate the reviewers' helpful comments and suggestions, as well as their overall positive feedback on our work! Their input has undoubtedly improved the clarity and strengthened the findings presented in the manuscript, and we believe this is reflected in the revised version. Due to the distribution of workload during the revision process, the order of the two first authors have been adjusted, while maintaining shared first authorship. In addition, we have modified the manuscript title, now reading "*Alginate foraging is conserved in geographically and taxonomically distinct ruminant microbiomes*" to clarify that the study concerns alginate degradation by microbes, rather than implying degradation of microbial alginate.

Please note that the line numbers in the responses correspond to the revised manuscript file *without* tracked changed. A version with tracked changes is available ("Tingley rumenAUL -TC").

Reviewer #1 (Remarks to the Author):

The manuscript entitled "Microbial alginate foraging is conserved in geographically and taxonomically distinct ruminant microbiomes" by Ferrillo et al. investigated the digestion and utilization of brown algae, specifically Saccharina latissima, within the gut microbiome of different ruminant species. The study employed both in vivo and in vitro experimental systems to demonstrate how gut microbiota break down this algae using specific microbial catabolic pathways, emphasizing the significance of this process in carbon cycling and sustainable biomass resources. Overall the study is relevant, however, the following comments should be well addressed.

Q1.1: In the introduction, the authors mention how ruminant microbial communities interact with novel carbon sources but lack a detailed discussion on the diversity and functional characteristics of these microbial communities. It is recommended that this aspect be elaborated to strengthen subsequent arguments.

RESPONSE: To address this concern, the text has been modified on **line 79-83** to include:

"In most cases, these events have been observed to occur within Bacteroidota, a phylum known to harbor complex polysaccharide utilization loci (PULs) or systems dedicated to saccharifying distinct polysaccharides. Exchange of PULs within Bacteroidota, promotes the acquisition of novel genetic machinery and the ability to subsist on available polysaccharide substrates [19]."

References added (numbers correspond to the main text):

19. Coyne MJ, Zitomersky NL, McGuire AM, Earl AM, Comstock LE. Evidence of extensive DNA transfer between bacteroidales species within the human gut. mBio 5, e01305-01314 (2014).

Q1.2: The paper does not fully consider the potential ecological impacts when discussing how ruminants adapt to novel carbon sources. The possibilities of niche competition and changes in microbial abundance with high proportions of algal feed are not adequately explored. It is advised to include this dimension.

RESPONSE: To address this concern, we have included new text describing adaptation to novel feedstocks and niche partitioning. The text has been modified in **line 477-494** to read:

*"Alginate is produced by Pseudomonas spp. found natively within the rumen [56, 57]. However, it differs from seaweed alginates, containing lower M/G ratios and high levels of O-2, O-3 acetylation of mannuronic acid [57, 58]. Therefore, although we show that dietary S. latissima results in expression of AULs, it remains to be shown if alginate produced by autochthonous bacteria help drive the evolution of these pathways. It has been previously speculated that xanthan gum utilization loci in human gut Ruminococcaceae evolved under selection of exopolysaccharides before being exposed to structural analogs found in food additives [59]. Likewise, it is possible that structurally related alginate substrates present a common niche for microbiota to fill in the rumen. One distinguishing factor is the absence of carbohydrate esterases in the seaweed-alginate associated MAGs, which is a conceivable requirement for bacterial alginate metabolism, suggesting there is differential specificity [13, 60]. It is unclear from our data how adaptation to alginate effects other microorganisms, as lower Shannon diversity with the 50% RUSITEC trial (**Supplementary Figure 3**) suggests overall lower microbial diversity and increasing algal content did not result in a noticeable shift in community structure. This suggests alginate does not function as a prebiotic in the rumen. In another study, it was found that carrageenan supplementation drastically altered the fecal microbiome composition, but not the rumen microbiome composition [61]. Thus, the role of the lower gut microbiota in alginate digestion should be explored in future work."*

<u>References added (numbers correspond to the main text):</u>

56. Kibegwa FM, Bett RC, Gachuiri CK, Machuka E, Stomeo F, Mujibi FD. Diversity and functional analysis of rumen and fecal microbial communities associated with dietary changes in crossbreed dairy cattle. Plos One 18, e0274371 (2023).

57. Urtuvia V, Maturana N, Acevedo F, Peña C, Díaz-Barrera A. Bacterial alginate production: an overview of its biosynthesis and potential industrial production. World J Microbiol Biotechnol 33, 198 (2017).

58. Remminghorst U, Rehm BH. Bacterial alginates: from biosynthesis to applications. Biotechnol Lett 28, 1701-1712 (2006).

59. Ostrowski MP, et al. Mechanistic insights into consumption of the food additive xanthan gum by the human gut microbiota. Nat Microbiol 7, 556-569 (2022).

60. Cha QQ, et al. Comparison of Alginate Utilization Pathways in Culturable Bacteria Isolated From Arctic and Antarctic Marine Environments. Front Microbiol 12, 609393 (2021).

61. Tingley JP, et al. Global distribution of microbial carrageenan foraging pathways reveals widespread latent traits within the genetic "dark matter" of ruminant intestinal microbiomes. bioRxiv, 2025.2003.2004.641501 (2025).

Q1.3: The study identified alginate utilization loci (AULs) in several MAGs. However, the authors acknowledge that, "due to genome fragmentation, the susC/susD pair and the alginate lyases were found towards the edges on two separate contigs, and thus, a complete AUL could not be determined". This suggests that while the study identifies key components of AULs, it is limited by the incompleteness of genome reconstruction and cannot present a complete AUL for all identified bacteria. This could hinder a full understanding of the mechanisms of alginate utilization.

RESPONSE: We thank the reviewer for this comment. Sequence fragmentation is an issue that is common in short read metagenomics and is a technical limitation of the technique (PMID: 30815668 and 35240043), as there can be many factors which may influence genome assembly (PMID: 27698619). Despite the limitation of sequencing data, we feel there are many high quality and complete mechanisms described which improve our understanding of alginate utilization within this manuscript.

Q1.4: The study notes that not all PL6 enzymes demonstrated efficient digestion of alginate. While some PL6 members produced substantial amounts of unsaturated products, others showed variations in catalytic efficiencies and activity. This suggests that the study could benefit from further investigation into the precise activities of each PL6 enzyme. While the study mentions that the complexity of M/G ratio and the ability of PL6 enzymes to change mode of action are factors, more detailed characterization could be beneficial.

RESPONSE: Although we agree that further characterization may provide some benefit, enzyme studies with alginate substrates are hampered due methodological limitations related to ManA and GulA chemistry (these C5 epimers are difficult to resolve by chromatographic techniques) and the lack of pure, commercially available substrates. Polymannuronic and polyguluronic

oligosaccharides can be up to 25% contaminated [Carbosynth], and purification of these substrates requires multiple methods and instrumentation (PMID: 22148348 and preprint doi.org/10.1101/2024.12.11.627697). For these reasons and those mentioned within the manuscript, commonly, alginate lyase papers do not often include structural determinants for specificity on mannouronosyl and guluronosyl configured substrates.

In an attempt to strengthen the study and provide more insight into the activities of PL6s reported here, we have performed digests for all the PL6 enzymes on two new substrates: commercially available polymannuronic acid and polyguluronic acid (Supplementary Figure 10). Further, kinetics were included for $MAG50_{BOV}$-A on alginate in Supplementary Figure 10. Minor activity was confirmed for all enzymes and confirmed using HPAEC-PAD (Supplementary Figure 11).

<u>New Supplementary Figure 10:</u>

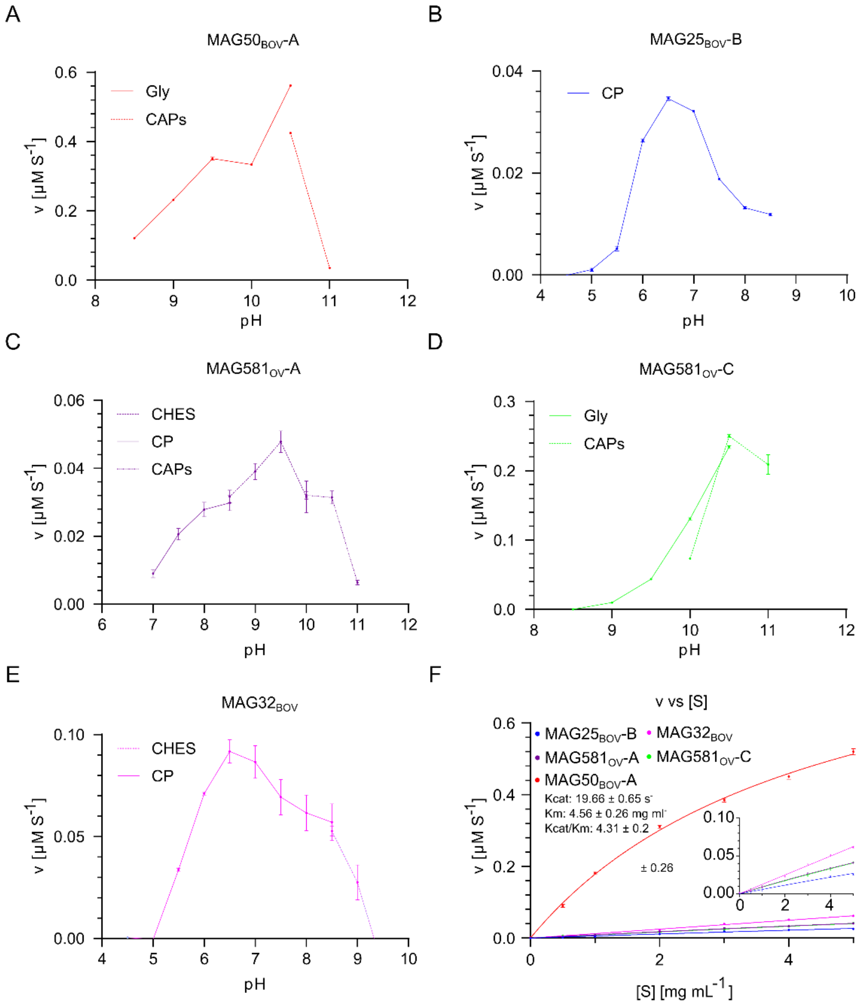

New Supplementary Figure 11:

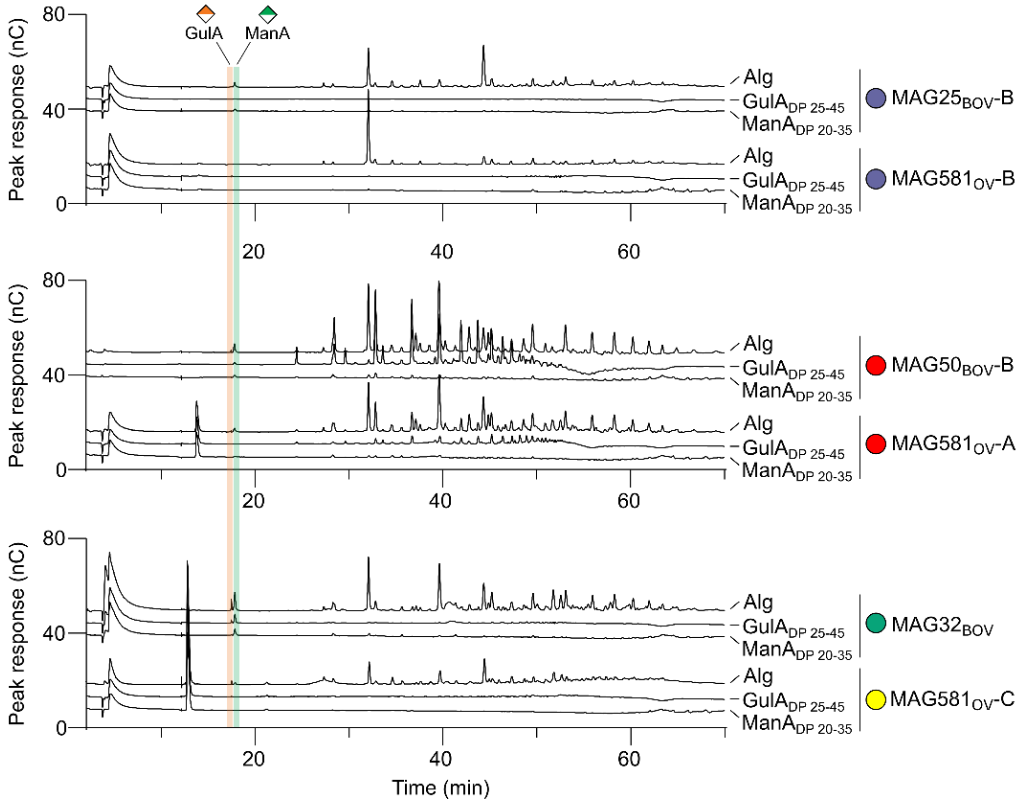

Q1.5: The metaproteomic analysis identified key proteins involved in alginate degradation, but it also mentions that other PL enzymes within AULs were not detected at the protein level. The study suggests some reasons for this, such as inclusion level, compartmentalized secretion, or insufficient adaptation time. However, further investigation into why these enzymes were not detected could be beneficial to enhance the understanding of the full metabolic response.

RESPONSE: While metaproteomics is a powerful method to detect active microbial functions in complex microbiomes, it is not 100% infallible and there can be technical limitations that lead to an absence of protein detection from a given PUL. As PULs are membrane associated, not all proteins are equally amendable to the extraction methods that are used to recover the proteome prior to digestion and LC-MS/MS analysis. Moreover, many PUL-associated CAZymes are fibre adherent, and can be inaccessible during the fibre dissociation steps that are used prior to protein extraction. In addition to the biological reasons that are already outlined in the manuscript, we have amended the text to discuss plausible technical reasons PLs were not detected in our metaproteome analysis (see **line 412-418**).

“This could be due to several technical factors, such as their amenability to protein extractions methods and their absolute levels being below proteomic detection thresholds. Moreover a plethora of biological factors could plausibly explain an absence of detection, including alginate specificity and/or percent inclusion level required to necessitate expression, compartmentalized secretion, specificity of induction products (i.e., which digest products induce AULs) [47, 48]*, or insufficient adaptation time for efficient utilization of seaweed. Others have suggested that alginate polymers decompose slowly* [46, 49]*.”*

Reviewer #2 (Remarks to the Author):

This manuscript addresses the question on seaweed catabolism in the rumen ecosystem of diverse livestock (lamb and cattle) and an 'artificial rumen' (RUSITEC). The authors provided orthogonal evidence (e.g., from multi-omics, physiology, biochemistry) that the ability to digest seaweed in diverse livestock around the globe likely is driven by alginate lyases with significant homology between different populations of mainly Bacteriodota. Thus, it seems that seaweed selects for specific members of microbiomes that are able to digest alginate regardless of the host species, which has implications not only for our fundamental understanding but also for seaweed as a supplement to mitigate the emission of climate active gases produced by livestock. Although I think this result is exciting and impactful, the manuscript requires improvements to provide more evidence for some of the claims made.

Main suggestions:

Q2.1: The strongest claim, which sort of reflects the main outcome of this study, is that the environment selects in terms of host animals and their ability to digest seaweed. As soon as there is seaweed in the rumen of any livestock, bacteria of the genus Bacteriodota, which encode the necessary gene cluster for digesting seaweeds, will express those genes and therefore help the host animals regardless of their diversity and geographical location. Thus, alginate catabolism appears to be a core functional trait 'performed by autochthonous members of the ruminant microbiome'. As this ability appears to be widespread, the authors speculate that ancient horizontal gene transfer events (possibly from a marine bacterium) must have contributed to the ubiquitous presence of those gene clusters in diverse rumen microbiomes across species and geographical boarders. The evidence provided to support this claim is weak, though. The reasons are as follows. Neither suppl. figure S4 nor S5 provide sufficient evidence of HGT (e.g., insufficient description of origin of MAGs, unclear bootstrap support, unclear nested clades). Thus, I suggest to apply proper phylogenetic methods (e.g., NOTUNG; Reconciliation methods such as a comparison between gene and species trees) and/or parametric or alignment-based methods. Ideally, this work will include a set of marine bacteria associated with seaweed and for which the presence of these gene clusters is known.

This comparison will help to start with disentangling the origin of those gene clusters. Divergence times can be estimated using coalescence theory. As this work is at the heart of this study, I suggest to show those results in the main paper and not the supplement.

RESPONSE: We thank the reviewer for their helpful recommendations to use alternative phylogenetic methods. We have included new analysis in this work including reconciliation and parametric methods. We believe this is now the first time these programs have been used to study HGT in PUL systems within the field. This are reported in the new version of the manuscript in the following ways:

Supplementary Figures 5 and 6 were improved by adding clear bootstrap values and kept in the study to represent closely related sequences. Further, NOTUNG (as recommended by the reviewer) was used to reconcile PL6 paralog groups and PL17 family members (See Figure 4; Supplementary Figures 4, 7, 12 and 13, and Supplementary Tables 4 and 5) and addressed in a new section of the manuscript in **lines 301-311**, which now reads:

*"The similarities between Bacteroidota PL6s from the gastrointestinal tracts of terrestrial animals and those found in marine/marine sediment microbiota suggested that some level of gene transfer may be involved in the acquisition of alginate saccharification. NOTUNG [38] was used to reconcile OrthoFinder species trees with PL17 and PL6s gene trees across 75 genomes from marine environments and terrestrial gastrointestinal tracts (**Supplementary Table 4**). PL6 members were either separated into paralog clades or kept together as an ortholog group. The presence and absence of PL6 and PL17 members were predicted to be a result of gene duplication and loss events, rather than horizontal gene transfer events (HGT). The only HGT event predicted was observed within Clade 2 between terrestrial host MAGs: MAG581OV (Paludibacteraceae sp.) and the Prevotellaceae family (MAG25BOV and MAGS2COV) (**Supplementary Figure 8; Supplementary Table 5**)"*

GC content and skew (source data only) was plotted for all AUL PLs against genome open reading frames (Figure 5) and addressed in **lines 313-315**:

"*Furthering this data, the GC content of the AUL/AUC-encoded PLs was comparable to the remainder of their respective genomes (**Figure 5C**), suggesting these genes were not part of a HGT event*."

Divergence times were estimated using BEAST2 analysis; however, the error was too large and was therefore omitted from the text.

The conclusion of these new analyses is that the origin of these genes is predicted to be inherited from a common ancestor, and there is limited evidence of horizontal gene transfer within this study. This finding not only helps to strengthen the current manuscript, but we believe, will provide a new benchmark for predicting PUL origins in the CAZyme field. The text

has been modified to accommodate these new findings, as outlined above. Figure 5 was further modified to match the new findings (please see figure below). We are thankful for the Reviewer's suggestions, which helped to clarify our conclusions.

<u>Modified Figure 5:</u>

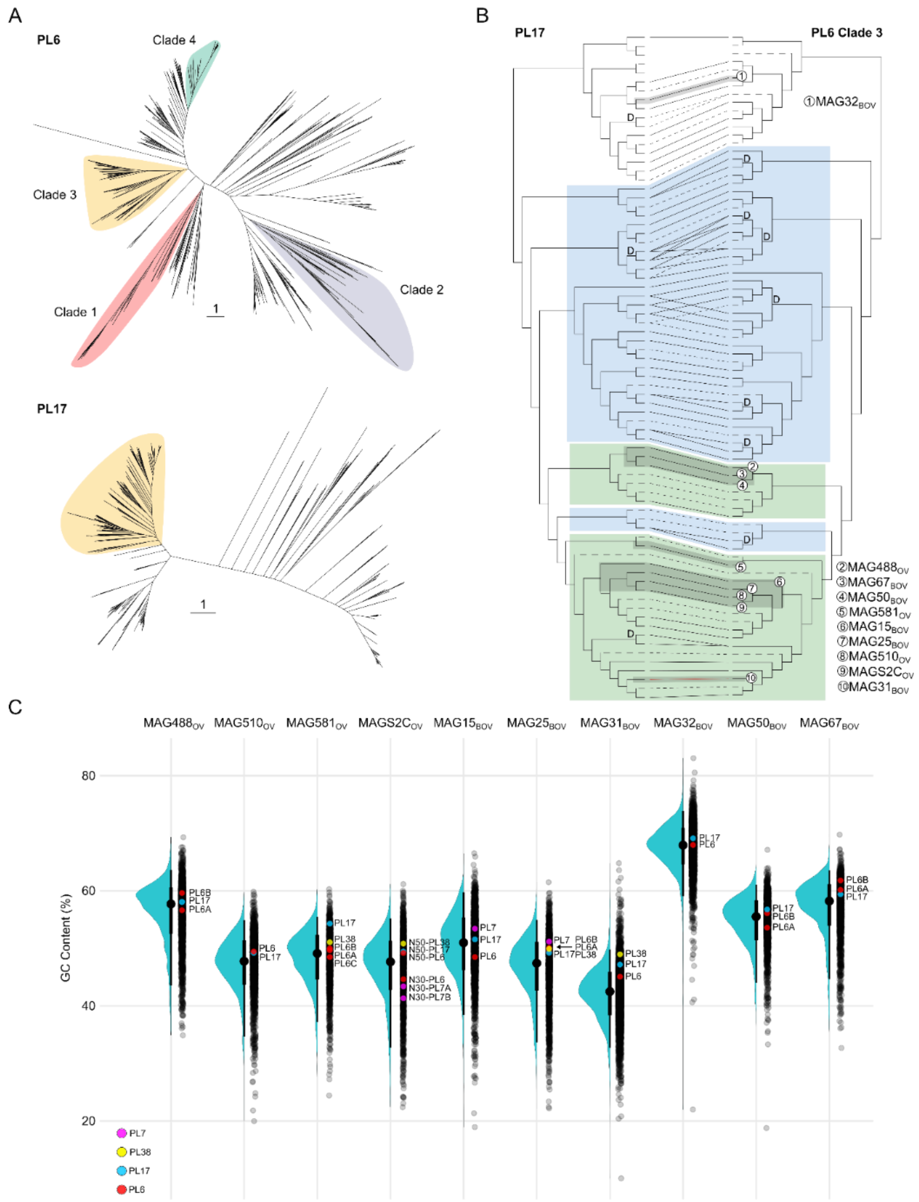

<u>References added (numbers correspond to the main text):</u>

38. Stolzer M, Lai H, Xu M, Sathaye D, Vernot B, Durand D. Inferring duplications, losses, transfers and incomplete lineage sorting with nonbinary species trees. Bioinformatics 28, i409-i415 (2012).

Q2.2: The work nicely links the sequence diversity (end evolution) of genes, the structure of enzymes and their activity. However, I was missing a structural comparison between the key enzymes using FoldSeek. I am sure this will provide additional insights into how the evolution of genes is linked to the function and structure of their encoded enzymes.

RESPONSE: We appreciate the reviewer's positive feedback, and the suggestion of using FoldSeek. Analyses using both FoldSeek and the Dali server were conducted. Unfortunately, these results did not provide any more insights than the primary sequence alignments done using Muscle or ClustalOmega, and therefore, did not improve data quality. However, to provide insights into the sequence-structure relationships of PL6s, AlphaFold models of all PL6s were generated and mapped to the corresponding phylogenetic clade. This figure is now included as Supplementary Figure 7.

New Supplementary Figure 7:

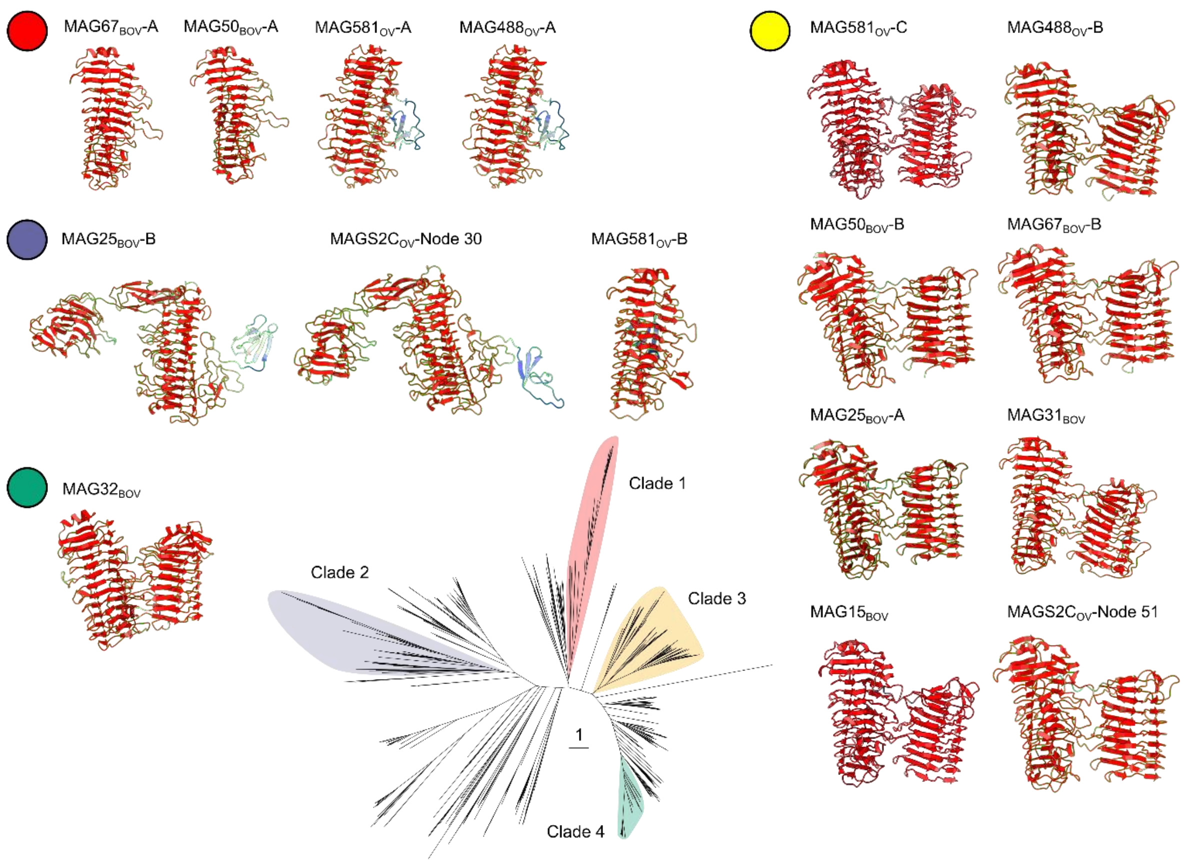

Q2.3: I was not able to find a proper synteny analysis although the authors claimed that this has been done. Figure 4a just shows the structure of the AULs but does not provide proper synteny information. There are many tools available for visualising syntenic relationships across multiple genomes such as https://academic.oup.com/bioinformatics/article/39/3/btad121/7072460 to only refer to one of them as an example. Any of those should be applied for synteny analyses.

RESPONSE: We appreciate the suggestion to consider specialized tools for synteny analysis and acknowledge that such tools are appropriate in many context. However, synteny analysis within the carbohydrate active enzyme and polysaccharide utilization loci field is not often done using a dedicated program. Also, due to the relatively small size of PULs, the available programs do not appear to work well as they were designed for analysis of chromosomal synteny (PMID: 35240043 and 31420336). In an attempt to address the reviewer's comment, we tried working with several programs for this analysis. We were unsuccessful in applying NgenomeSyn, however, its core software minimap2 was successfully used. Still, the results provided less insight for the study compared to amino acid alignments of individual proteins. We also attempted the new synteny program Lovis4u (preprint doi.org/10.1101/2024.09.11.612399), however, synteny programs based on ortho-groups incorrectly grouped all PL6 members. Therefore, we revisited the data and improved the alignment between AUL/AUC sequences using Clustal Omega (Supplementary Data 1, sheet 7: *PUL similarities*). These results were mapped manually to the figure and include links between PL family members to generate a new, high-quality figure (Figure 4). We think this outcome provides a more accurate and convincing analysis of synteny between the AULs.

Q2.4: Maybe I missed this (line 388), but would it be possible to try to estimate the relative abundance of those ruminal bacteria with the gene clusters for metabolising alginate? As 16S sequencing was done, this should be doable.

RESPONSE: We thank the reviewer for this comment. Relative abundance estimates are best performed via CoverM (Supplementary Data 1, sheet 2: *Lamb MAG tax.*) as 16S sequence data is not suitable for strain level detection (which would be crucial for the AUL encoding *Prevotella* strains). CoverM analysis was conducted and included in Supplementary Data 1. To clarify this in the manuscript, we have added the following text to **line 212:**

*"The relative abundance of all MAGs can be found in **Supplementary Data 1**."*

Q2.5: The abstract is a bit unusual as there is barely any information about the results and a brief discussion including concrete implications is missing, too. Hence, I suggest rewriting it.

RESPONSE: Thank for the suggestion. We have rewritten parts of the abstract to introduce more results, a brief discussion, and a concluding statement to highlight the implications of the study.

Q2.6: Figure 3b is a bit too small considering its importance.

RESPONSE: The overall size of Figure 3b, as well as the font size of the text within, has been increased.

Q2.7: Depending on the outcome of the evolutionary analyses I have suggested above, the trees shown in the main manuscript likely need to be reconsidered. Maybe at least one of them can be moved to the supplement.

RESPONSE: The manuscript has been modified to include the evolutionary analysis and NOTUNG reconciliation (please see response to first comment). The PL6 tree that was formally in Figure 4 has now been moved to Supplementary Figure 5 and 6.

Q2.8: The doi:10.5281/zenodo.14515213 for looking at the code did not work for me. There was no entry at Zenodo under this DOI.

RESPONSE: We thank the Reviewer for finding this oversight. The issue has been addressed and the data is now open access under the original DOI.

Q2.9: Last but not least, I was wondering about the novelty of this work as the authors referred to similar work several times in their manuscript. Thus, this needs to be made clear. Also elaborate more on the implications.

RESPONSE: We thank the reviewer for pointing out this unclarity. This manuscript significantly improves the knowledge of livestock adaptation to novel feedstock. To our knowledge, this is the first manuscript to describe ability of the ability of ruminant microbiomes to degrade seaweed polysaccharides, as most studies focus only on microbial composition. This finding is confirmed using two geographically distinct systems to demonstrate the conservation of alginate utilization between two different ruminant genera (Bos and Ovis). Further, in response to reviewer concerns, this article is the first to use gene-species tree reconciliations to confirm or disprove horizontal gene transfer of seaweed degrading enzymes in mammalian systems and provides a new benchmark for substantiating claims of PUL origins.

We have edited the conclusion accordingly to read (**lines 522-527**):

"Here, using molecular informatic approaches, we investigated the ability of two geographically and taxonomically distinct ruminant microbiomes to adapt to S. latissima, a widely distributed, commercially grown brown seaweed. Our study demonstrates that rumen Bacteroidota species contain highly conserved AULs that are inherited through vertical transmission from ancestral species, and are upregulated in S. latissima supplemented diets."

Reviewer #2 (Remarks on code availability):

The doi:10.5281/zenodo.14515213 for looking at the code did not work for me. There was no entry at Zenodo under this DOI.

RESPONSE: We apologize for the inconvenience; the issue has now been solved. Please also see the response to Q2.8.

Reviewer #3 (Remarks to the Author):

Ferrillo et al. study the ability and mechanisms of rumen microbiota to degrade non-terrestrial (alginate) polysaccharides. The authors found that several microbes from two different ruminants have the ability to degrade some of the polysaccharides derived from brown algae. The authors carefully analyzed the genes, their expression and for some also the specific degradation products. They also showed that some microbes bind alginate specifically using a fluorescence based assay. Overall this study provides new and important insights into the potential for the microbiota to provide ruminants with the ability to use forage that is not part of their typical diet. While I think this research will be an important contribution to the body of knowledge on how the microbiota is involved in accessing different dietary components there are some concerns that would need to be addressed.

Major comments:

Q3.1: It is a bit surprising to me that microbes in the rumen have the ability to degrade alginate, which they usually do not have access to (except for the sheep which seem to be exposed to this regularly). I would expect that genes that are not used by these microbes would be lost or degrade over longer evolutionary times as they would represent an unnecessary physiological burden even if tightly regulated. Could these "alginate" degradation genes potentially be involved in the degradation of other polysaccharides that the rumen microbiota may "see" more frequently, which would explain their retention in their genomes. It would be great if the authors could speak to this "hypothesis" in their article particularly if the answer is "no". I do feel that the argument why these genes are retained is currently weak and it could be strengthened based on existing literature on glycan degradation pathways in gut microbiota across different animals.

RESPONSE: We appreciate the Reviewer for highlighting this point, which indeed capture a central aspect of our findings. As we believe that this a key feature of the study, we agree that it would be useful to address this more directly in the manuscript. We thank the reviewer for this insightful comment, and in response, we have modified the main text at **lines 477 to 487** to state:

"Alginate is produced by Pseudomonas spp. found natively within the rumen [56, 57]. However, it differs from seaweed alginates, containing lower M/G ratios and high levels of O-2, O-3 acetylation of mannuronic acid [57, 58]. Therefore, although we show that dietary S. latissima results in in expression of AULs, it remains to be shown if alginate produced by autochthonous bacteria help drive the evolution of these pathways. It has been previously speculated that xanthan gum utilization loci in human gut Ruminococcaceae evolved under selection of exopolysaccharides before being exposed to structural analogs found in food additives [59]. Likewise, it is possible that structurally related alginate substrates present a common niche for microbiota to fill in the rumen. One distinguishing factor is the absence of carbohydrate esterases in the seaweed-alginate associated MAGs, which is a conceivable requirement for bacterial alginate metabolism, suggesting there is differential specificity [13, 60]."

See response to Q1.2 (Reviewer #1) for reference added.

Q3.2: The article has a LOT of abbreviations, many of which are not introduced, which makes it hard to read in some sections. While some of the glycan related abbreviations may be known to experts in that particular field a general reader such as myself really struggles with those. I would suggest to either write out terms, or provide a box in the article that gives an overview of the most used abbreviations.

RESPONSE: Although this shortform nomenclature carbohydrates is common within the glycobiology field, we appreciate that this system may be confusing to the general readership. However, we are unsure what the journal's policy is on including an inset for abbreviations. We would be happy to include if approved by the Editor. Alternatively, we have generated a supplementary table (Supplementary Table 8) to define each abbreviation. A reference to the table is indicated in **line 843**. Additionally, we have removed abbreviations such as GIT (gastrointestinal tracts) and MLG (mixed-linkage glucans).

Q3.3: The analysis of where the relevant genes and gene clusters have come from was a bit lacking. On page 21 there is a discussion that essentially concludes that these genes/gene clusters must have been in ruminant microbes for very long evolutionary times, but the evidence provided is weak. With your existing data you could do much more to investigate this question i.e. was it a recent gene transfer, are these genes very mobile etc. Generally I would actually expect high gene mobility based on the literature, that has shown that the glycan degradation gene clusters move around a lot by HGT. You could for example do more extensive phylogenetic analysis (and show them in more detail) of the relevant genes checking what free living and other gut microbes they have been found in, and if the 16S rRNA gene phylogenies are congruent with those gene phylogenies, which would support long term retention of the genes in those lineages, or alternative (my expectation) that the phylogenies of the genes and

the 16S rRNA gene are not congruent which would indicate rampant horizontal gene transfer. Additionally, you can look at GC biases and GC skew to see if these cluster may be from recent integration events. Finally, you can map the reads from your metagenomes to the contigs and see if you have any obvious dips in read coverage, which would indicate heterogeneity in the relevant genome regions due to insertions and deletions.

RESPONSE: A similar point was also raised in Reviewer #2's Comment 1 (Q2.1). In response to both reviewers' comments, we performed additional analyses as outlined in detail above. We agree that these new analyses significantly strengthened the manuscript, and we thank the reviewers for the valuable feedback.

Q3.4: The data in Figure 6B is a bit unexpected in that binding to the FLA-SLAT is increased even in controls after 1 day. It would be good to explain better what these observed patterns could mean. I would have for example expected much higher binding in the 50% samples. Also, is there any replication of this data? i.e. could differences be tested for statistically. Right now this data is kind of qualitative.

RESPONSE: This data was done with a single replicate as a qualitative measurement, repeated at two concentrations (2% and 50%). We believe these data confirm the interaction of microorganisms within the bovine rumen microbiome with alginate and provides a valuable contribution to the manuscript; however, we agree that without replications and statistical analysis, the data should be removed as a main figure. Therefore, we have removed the quantitative bar chart, and moved the fluorescent imaging and corresponding material and methods to Supplementary Figure 4 and Supplementary Text 2, respectively. We have modified the text accordingly and moved the results to **lines 162-167**, with a reference to the elements in Supplementary Information.

Q3.5: The analysis of the metaproteomic data in Figure 3 is a bit limited and is quite "cherry picked". I would be interested to know what some of the other very significant/different proteins are. I think understanding the response to the seaweed diet more holistically would be good. Adding gene names for more proteins to the volcano plot would be great and then at least one paragraph discussing some of the additional observed differences.

RESPONSE: We appreciate the Reviewer's interest in the metaproteomics data and the suggestion to provide a broader perspective on the ruminal microbiome response to seaweed diet. In the initial stages of the analysis, we did indeed attempt a pathway-oriented interpretation of metaproteomics data. This proved challenging as several of the proteins had ambiguous functions, making confident reconstruction of metabolic pathways and cross feeding difficult. To avoid overly speculative interpretations, we therefore chose to focus on proteins directly relevant for alginate deconstruction (i.e. alginate lyases). While we do agree on the

value of a holistic interpretation, expanding the manuscript with additional (and speculative) discussions may not be ideal given its current length. However, we agree that highlighting additional proteins with a significant response the diet may provide valuable information for further investigations. As such we have highlighted proteins significantly (FDR-adjusted p-value) up-regulated proteins (only seen in *S. latissima*-diet) in the volcano plot.

Q3.6: I wonder if it would make sense to add some framing regarding the hologenome theory of evolution to this manuscript. Essentially acquisition of microbes for rapid acquisition of new functions would argue for the hologenome theory, of course your current evidence may not support the theory too much, but once you look more about where these genes came from there may be a clearer story.

RESPONSE: Hologenome theory is certainly an interesting perspective to explore in the context of the ruminant microbiome gaining alginate-foraging capabilities. However, we believe this comment, which is a great continuation to **Q3.C1 above**, has been somewhat addressed in our response to Q3.C1, Q1.2 and Q2.1 and does not require a specific response herein. Across our research to produce this manuscript as well as its revision (e.g. Q3.C1), we found a lack of evidence to suggest that the hosts behaviour in selective foraging on dietary seaweed alginate acts as the primary driver for the essential acquisition of alginate-degradation in ruminant microbiomes. Rather, a hypothesis emerges that ruminal alginate digestion is plausibly a latent trait that is carried within the microbiome of ruminant livestock. See **Q3.C1**, **Q1.2** and **Q2.1** for more details.

<u>Minor comments:</u>

1. The last sentence of the abstract (conclusion sentence) is vague. This could be more specific and stronger.

RESPONSE: We have made significant changes to the abstract in response to Reviewer #2 comments (Q2.5). The concluding sentence has been changed to be more conclusive:

"The conservation of these systems indicates that the ruminant microbiome retains a latent capacity to metabolize marine polysaccharides."

2. Graphical abstract is great, very clear.

3. Introduction is very clear.

RESPONSE to minor comment 2 and 3: We're glad to hear that these sections were well received.

4. L. 98, delete “high” or rewrite to similarity or identity. Homology is an absolute and there is not levels of homology.

RESPONSE: Thank you for the helpful comment. “Homology” has been replaced with “identity”. These edits have been made through the manuscript.

5. L. 104, replace “their” with “kelp”

RESPONSE: This change has been made.

6. L. 104 to 108, long sentence in passive voice, split the sentence and rewrite to active voice.

RESPONSE: The sentence has been modified, and now reads:

"To determine the linkage composition of S. latissima, kelp harvested off west coast of Canada was subjected to GC-MS based glycosidic linkage analysis using a recently established procedure optimized for unfractionated cell wall of brown seaweeds."

7. L. 108 to 110, unclear to me what these compounds are, add some simple explanations for broader audience

RESPONSE: Please see response Q3.2 above.

8. Page 6, too many not explained abbreviations

RESPONSE: Please see response Q3.2 above.

9. L. 152, Shannan should be Shannon, also explain what the reader is supposed to learn from the comparison of these indices.

RESPONSE: The spelling error has been corrected. Shannon diversity is now explained in greater detail in **lines 487-491**:

*“It is unclear from our data how adaptation to alginate effects other microorganisms, as lower Shannon diversity with the 50% RUSITEC trial (**Supplementary Figure 3**) suggests overall lower microbial diversity and increasing algal content did not result in a noticeable shift in community structure.”*

10. L. 157, whose “metabolic metrics”, the sheep? For the reactors I would not know what this means. Please explain better.

RESPONSE: The text has been modified to:

*" In both rumen systems, the digestibility of seaweed was observed with no detrimental impacts (**Supplementary Text 1**).”*

11. L. 173, “We therefore extended…” I do not understand what this refers to. How did the refinement work? Did you look at the neighboring genes of the genes of interest?

RESPONSE: Thank you for this comment. We have modified the text to describe "neighboring TonB-dependent receptors."

12. L. 179 – 186 this paragraph is much clearer in its writing, maybe you can model some of the previous paragraphs on it.

RESPONSE: Following revisions, several of the previous paragraphs have been improved.

13. L. 200, since you specify that they were not detected in “fiber attached rumen samples”, were they detected in the non-fiber associated fraction?

RESPONSE: According to relative MAG abundance, these two *Prevotella* MAGs, $MAG510_{OV}$ and $MAGS2C_{OV}$, demonstrated a clear dose-dependent response to inclusion of *S. latissima* in the fiber-attached samples. Their MAG abundance in the rumen fluid samples was showing a similar dose-dependent pattern, yet with detection, albeit low, in two of the control sample replicates. The abundance estimated of all MAGs in fluid and fibre-attached fraction for all samples are available in Supplementary Data 1; sheet 2 *Lamb MAG tax*.) *Prevotella* is well known as a dominant member of the planktonic rumen community, where they primarily utilize soluble and easily accessible substrates rather than complex plant fiber (which are more commonly associated with fibre-attached microbial populations).

14. Line, 205, delete “After filtering,”

RESPONSE: This correction has been made.

15. Line 206, add “the” before detection

RESPONSE: This correction has been made.

16. L. 210, add “a” before “dose-dependent”

RESPONSE: This correction has been made.

17. L. 215, so how similar are the two respective MAGs in terms of ANI and how does this relate to the PL6 similarity?

RESPONSE: According to Sourmash, $MAG510_{OV}$ and $MAGS2C_{OV}$ has ANI 98.19% which does correlate to the high degree of similarities between the PL6s in these two MAGs. This was further corroborated via the species trees developed with OrthoFinder.

18. L. 216, What do you mean by “functional redundancy”? some more specific explanation may be needed.

RESPONSE: Thank you for pointing out this imprecise phrasing, we have now added "*functional redundancy in their substrate specificity*" to the end of the sentence to provide more clarity.

19. L. 230, fragmentation is usually due to strain variability in the population that you bin into one MAG. You can re-assemble these MAGs after read mapping to them, and then use linkage graph analysis for example with Bandage to see what fragments are linked together, this will allow you to deduce if all AUL genes are in one cluster. i.e. the linkage analysis shows you which contigs are neighboring.

RESPONSE: In response to this comment, we attempted to reassemble the genome of $MAG510_{OV}$, by mapping the quality-trimmed reads back to the original $MAG510_{OV}$ contigs using BWA MEM. To ensure high specificity, reads from samples with the highest abundance of the MAG were recruited, and only primary alignments with a mapping quality score ≥ 30 were retained using Samtools. Reassembly was performed with SPAdes in --*careful* mode, using the original MAG contigs as guiding sequences (--*trusted-contigs*). Unfortunately, this approach only marginally improved the genome reconstruction and failed to confidently resolve the region of interest. The resulting assembly graph was dominated by unlinked nodes, likely a consequence of low coverage. The high degree of strain heterogeneity necessitates the stringent filtering to ensure specificity, which in turn results in lower coverage – a trade-off between sensitivity and completeness. Resolving these issues may require isolation-level sequencing and/or high quality long-read sequencing, which fall outside the scope of the current study. Nevertheless, we

greatly appreciate this valuable suggestion and intend to implement it in future studies facing similar challenges.

20. Figure 3, I am wondering if you normalized the protein abundance data to each organism? If not some of the differences could be caused by differences in overall organism abundance and not in differences in within organism protein abundances i.e. compositionality of the data could be an issue here. You may want to double check that your results are the same for organism normalized data.

RESPONSE: In the current analysis, protein abundance data were not normalized at organism level, as our focus was to identify differentially abundant proteins across the diets. To address the Reviewers valid concern, we performed an organisms-level normalization of the detected alginate lyases (PL6) for $MAG510_{ov}$ and $MAGS2C_{ov}$. Specifically, the non-log transformed LFQ intensity of the target PL6 was normalized to the total LFQ intensities of all proteins assigned to that MAG in that sample. The observed pattern remained consistent with the original interpretation. We have included the following description in the Materials and Methods (**line 786-790**):

"To account for differences in organism abundance, organism-level normalization was performed for target enzymes. For each MAG and sample, the linear LFQ intensities of the target protein was divided by the total LFQ intensities for all proteins assigned to that MAG. Normalized values are provided in ***Supplementary Data 2.***

21. L. 253, replace homology with similarity, again, homology does not have levels

RESPONSE: This correction has been made (and throughout the manuscript).

22. L. 254, delete "homology"

RESPONSE: This correction has been made.

23. Figure 4B, Why do some of the arrow head not have numbers? Why is there no "green" square in the legend? The subfamily, clade terms being different (?) is confusing

RESPONSE: This figure has been modified and no longer includes this phylogenetic tree.

24. Line 261, replace "metagenomics" with "metaproteomics"

RESPONSE: Thank you for noticing this error, the correction has been made.

25. Line 260, something is missing in that sentence, it is incomplete

RESPONSE: This figure caption has been modified to:

"Figure 4: Synteny between lamb and cattle alginate AULs*. OrthoFinder was used to generate a species tree composed MAGs containing AUL/AUCs. ClustalOmega, BLAST, and SACCHARIS were used to identify orthologs and similarity between AUL/AUC genes. The AULs and AUC were created with the gggenes package. Bolded genes were identified within the lamb metaproteomics."*

26. Line 260, insert "The representations of AULs…"

RESPONSE: See above change.

27. Line 270, "amino acid homology" is incorrect, see comments above

RESPONSE: This correction has been made.

28. Line 278, no clue what poly-MG is

RESPONSE: This phrase has been improved (**line 289**): "...an enzyme active on poly-MG alginate blocks (consisting of both Guluronate: G and Mannuronate: M)."

29. Line 280, "exo-type" not sure if this is a scientific term

RESPONSE: Exo-type is a commonly used description of enzymes that attack the terminal end of a substrate and release chemical identical products. In the case of carbohydrate active enzymes, exo-type attack the non-reducing end. This concept was proposed in the following review: DOI: 10.1042/bj3210557.

30. Line 284, endo-poly-MG???

RESPONSE: Please see responses above (28 and 29).

31. Figure 5A, What is GulA in Gel picture, and GulA, ManA and Alg. In chromatogram, add to caption? What was the negative control in the gel, please describe in caption.

RESPONSE: The controls have been described in greater detailed in the Figure 6B caption: "TLC and HPAEC-PAD product analysis of brown seaweed alginate digested with lamb and cattle-associated MAG PL6 enzymes. ManA and GulA represent monosaccharide standards."

32. Figure 5 B & C, can you also show the negative controls for these measurements to show that these are specific mass peaks

RESPONSE: The figures shown in the manuscript are MS2 fragmentations which were not observed within the control samples:

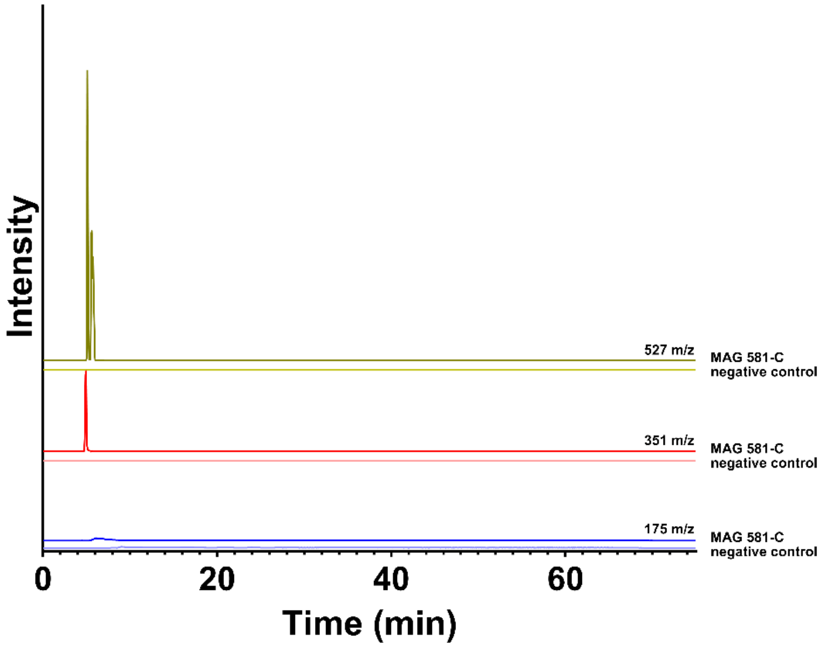

The exception is m/z 175 (the predicted monosaccharide). However, this peak does not fragment as a carbohydrate:

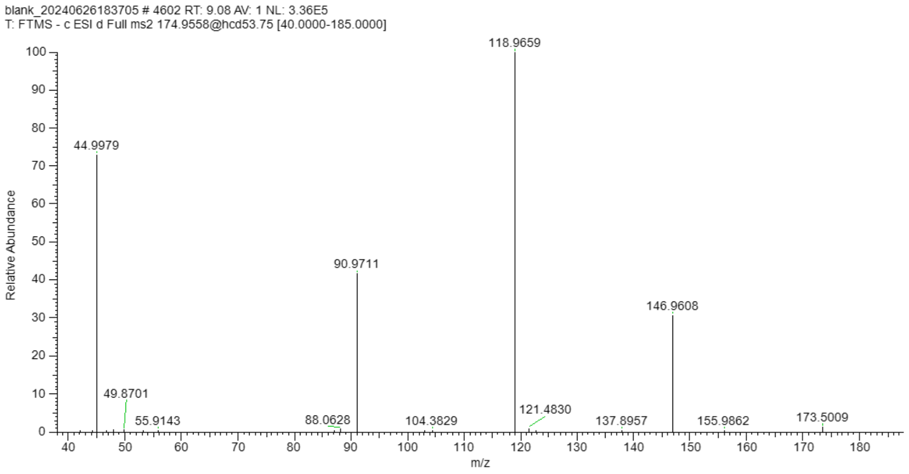

This control data was not included in the manuscript as it is typically not included.

33. Line 334, remove the “-“ between 10 and 7, this would be a terribly small number otherwise

RESPONSE: The quantitative analysis (auto cell counting) has been removed because there was only a single replicate. The imaging has been moved to Supplementary Figure 4 because it shows a qualitative response of microbial cells to fluorescent probes at two concentrations. The controls for this image are imaged under the same conditions.

34. Line 337 and 338, I do not understand what is referred to here. Needs to be written more clearly.

RESPONSE: See comment 33.

35. Line 339, what does it mean that the control also takes up the SLAT

RESPONSE: Please see comment 33.

36. Line 344/345, How do you explain this observation, it is counterintuitive, so it would be good so say something about it in way of explanation.

RESPONSE: See comment 33.

37. L. 338, do you really mean central role in general, or specifically a central role in alginate degradation.

RESPONSE: To improve this statement, we have removed "central". The sentence (**line 408-410**) now reads:

"This suggests that a limited number of specific rumen microbial species play roles by occupying a specialized niche for digestion of seaweed-derived polysaccharides."

38. L. 391, delete “protein”, proteins are not expressed, genes are expressed and the product of gene expression are proteins.

RESPONSE: This correction has been made.

39. L. 392, No idea what “inclusion level” refers to, also no idea about “specificity of induction products”

RESPONSE: The text has been modified to address this issue on **line 414-418**:

"Moreover, a plethora of biological factors could plausibly explain an absence of detection, including alginate specificity and/or percent inclusion level required to necessitate expression, compartmentalized secretion, specificity of induction products (i.e., which digest products induce AULs) [47, 48], or insufficient adaptation time for efficient utilization of seaweed. Others have suggested that alginate polymers decompose slowly [46, 49]."

40. L. 450, non-sequitur between the two sentences, make sure to add a transition that links the sentences

RESPONSE: The text has been improved as suggested (**lines 471-476**), and now reads:

"Recent studies have also revealed that wild Svalbard reindeer populations are adapting to the warming Arctic climate by supplementing their diet with kelp, driven by an increasing prevalence of ice-locked pastures [55]. How these animals survived a radical change in their diet and lifestyle, and the evolutionary processes that drove microbial adaptation towards seaweed polysaccharide catabolism have not been thoroughly studied."

41. L. 454-476, rewrite based on major comment, currently the argument is not very clear.

RESPONSE: As requested, this text has been substantially modified (**lines 477-516**). We would refer the reviewer to the text for evaluating this new section.

42. L. 477, very vague ending of the sentence, maybe say something more specific about what the clues are and what we learn from it, right now I have to guess.

RESPONSE: We agree that there is not a suitable system to study this currently. At this stage of the discussion we thought it appreciate to speculate of some alternative ecological mechanism for the evolution of this pathway.

43. L. 488, the argument for the "be native to the ruminant microbiome" was not well presented above

RESPONSE: This concept is now discussed in greater detail, please see:

Lines 402-405: *"Likewise, PL17 members were found co-located with PL6 subfamily 1 members in Clade 3 [13, 16]. In contrast, members of PL6 subfamily 2 and subfamily 1 Clade 1 appear to be ancillary components, likely arising from gene duplication events."*

Lines 500-503*: "The conservation of PL6 and PL17 pairs within Bacteroidota species from geographically and taxonomically distinct ruminants suggests that these genetic loci were acquired through one or a series of evolutionary events, possibly via speciation or duplication. However, there is little evidence of horizontal gene transfer."*

Lines 508-509: *"In this manner, the maintenance of seaweed alginate catabolism in the absence of substrate availability represents a "latent trait" within the rumen microbiome."*

44. L. 489-491, vague, be more specific about what these hypotheses are

RESPONSE: To avoid confusion, we have removed the reference to hypotheses and changed the concluding sentence to read (**line 529-531**):

"This suggests that ruminant-associated microorganisms may have adapted to other exotic polysaccharides and novel forages by the retention of latent traits that are carried within the microbiome of ruminant livestock."

45. L. 674, change > to <

RESPONSE: This correction has been made.

46. L. 698, Since no details about peptide preparation are given either provide more text here, or provide an open access reference, or put it into the supplemental materials.

RESPONSE: This information has now been included in the Methods section, **line 757-759**.

47. L. 713, Please add some information of how the data was normalized

RESPONSE: Data normalization was performed using FragPipe's build-in IonQuant tool, with median centered normalization (--normalization 1) enabled. This is now indicated in the text.

48. L. 720, add all parameters for the imputation sampling

RESPONSE: This information has now been included.

49. L. 722, It sounds as if you did not apply a correction for multiple hypothesis testing? Please do double check that things remain significant when you correct for multiple hypothesis testing using either the permutation based FDR or the Benjamini Hochberg FDR. Generally it would be

better to report FDRs or q-values rather than p-values, which are not as meaningful in high-dimensional omics data.

RESPONSE: We have now applied permutation-based FDR corrected p-values for a more rigorously statistical analysis. As a result, a substantial portion of the detected proteins are no longer considered significant at 5% FDR, however, the key protein SusD remains significantly up-regulated in the seaweed-supplemented dietary group. In the revised version of Figure 3A, all proteins which are significantly (FDR-adjusted $p < 0.05$) upregulated in the seaweed-supplementary diet are highlighted with callouts including protein function, MAG-affiliation and the FDR-adjusted p-value. Accordingly, the following sentence has been added to the Methods section (**line 784-786**):

"Protein groups with significant changes in LFQ intensities were identified using a two-sided Student's T-test with a permutation-based FDR correction (FDR < 0.05, S0=0.1)."

50. L. 822, change "is" to "are"
RESPONSE: This correction has been made.

Response to Reviewer #1 Comments

REVIEWER COMMENTS
Q1: We acknowledge the inclusion of NOTUNG reconciliation and GC-content analysis addressing HGT concerns. However, the failure to perform codon usage bias analysis and exclusion of marine Bacteroidota genomes renders the vertical inheritance claim speculative. Provide CAI/ΔENc’ metrics and integrate marine lineages (e.g., Zobellia) to conclusively reject ancient HGT.

RESPONSE: Regarding the codon usage bias analysis: we feel that GC content bias as well as exhaustive species-gene tree analysis is more than sufficient to underpin the phylogeny of these enzymes and microorganisms. Further analysis would be redundant. Furthermore, we are confused by the reviewer’s request to include marine *Bacteroidota* in the analysis. Marine organisms were selected based upon the phylogenetic relationship to the clades discussed in this study, which is why *Zobellia* was not included. **Numerous other marine *Bacteroidota* were incorporated** in this analysis, including: *Aquimarina* sp. AD1; *Aquimarina* sp. AD10; *Aquimarina* sp. ERC-38; *Cellulophaga lytica*; *Cellulophaga lytica* HI1; *Draconibacterium orientale* DSM 25947; *Dysgonomonas gadei* ATCC BAA-286; *Flavobacterium kingsejongi* WV39; *Flavobacterium nackdongense* GS13; *Flavobacterium* sp. B183; *Flavobacterium* sp. KBS0721; *Mariniflexile* sp. TRM1-10; *Owenweeksia hongkongensis* DSM 17368; *Polaribacter* sp. BM10; *Polaribacter* sp. KT25b; *Polaribacter* sp. MED152; *Polaribacter* sp. SA4-10; *Saccharicrinis aurantiacus* HQYD1; *Saccharicrinis fermentans* DSM 9555; *Tamlana carrageenivorans* UJ94; *Tamlana fucoidanivorans* Cw2-9; *Tamlana* sp. s12.

Q2: The supplemental enzyme data (poly-M/poly-G activity) is noted. Nevertheless, the absence of kinetic constants (kcat/Kmkcat/Km) for all PL6-substrate combinations and undefined activity thresholds invalidates functional conclusions. Tabulate full Michaelis-Menten parameters and adopt CAZy efficiency benchmarks immediately.

RESPONSE: As stated numerous times both in the manuscript and in revision, this is not a trivial task, nor was this work a major focus of this manuscript. Both substrate heterogeneity, viscosity, and the substrate-product conversion of endo- active enzymes prevent proper Michaelis-Menten kinetic determination, and defined substrates not commercially available. This is well understood in the alignate lyase literature. Further, the production of non-carbohydrate end products, including DEH (see **Figure 6B** and **C**), results in UV signal decay, masking real-time generation of unsaturated products and preventing steady state kinetics analysis.

Q3: Claims of "compartmentalized secretion" or "extraction inefficiency" require empirical validation via spike-in recovery experiments. Quantify losses of commercial alginate lyases (e.g., Sphingomonas AlyA) in rumen matrices—not theoretical speculation.

RESPONSE: We strongly presume the reviewer can appreciate that metaproteomic analysis of endogenous rumen microbiomes is an extremely complex process and is not an exact science. What the reviewer is proposing is a time-intensive, technical benchmarking of general metaproteomic methodology, which is out-of-scope and a complete experimental repeat. Moreover, we would like to highlight that we observed **clear differential detection of**

key transport (SusC) and binding lipoproteins (SusD) as well as alginate specific PLs co-located within the same alginate AULs in the rumen of animals consuming alginate (see **Figure 3**). Decades of PUL research has repeatedly showcased their substrate specificity and their regulatory response to specific substrate exposure. Moreover, our lab has shown over the years that substrate specific PULs, which have been elucidated at high resolution in culture studies (see **Figure 2** doi: 10.1038/s41467-019-08812-y), are scantly detected in their entirety in the complex gut metaproteome of an animal fed the same substrate (see Figure 5, DOI: 10.1038/s41467-020-19585-0). We strongly feel our discussion on the topic is a careful and balanced assessment of the limitations of metaproteomics, while acknowledging the strength of the partial AUL detection. We also note that **Prof. Manuel Kleiner** who is **Reviewer #3** and is one of most prominent leading experts in metaproteomics, found our responses acceptable.

Q4: Attributing reduced Shannon diversity to "technical factors" without co-occurrence networks or SCFA profiling is unacceptable. Correlate Prevotella dominance with taxon displacement and measure metabolic outputs to prove functional resilience.

RESPONSE: We believe the reviewer is responding to reviewer comment 3. The reference of "technical factors" is not relevant to these data and we do not make any claims that Shannon diversity being reduced as a result technical factors. We believe the reviewer may be referring to the 50% inclusion trial which demonstrated a reduction in Shannon diversity in comparison to the control trials. The measurement of metabolic factors is completely beyond the scope of this study.

Q5: "Dose-dependent" MAG claims lack multiplicity-corrected p-values and effect sizes. Publicly archive raw data with full statistical documentation—retract any unsupported inference.

RESPONSE: To address this comment, we assessed the correlation between MAG relative abundance and dietary seaweed inclusion levels (0%, 2.5% and 5.0%) using Spearman's rank correlation. For each MAG encoding alginate lyases, as highlighted in Figure 2, we calculated the Spearman's correlation coefficient (rho) as an effect size, along with the raw p-value. We then applied Benjamini-Hochberg FDR method to correct for multiple testing (adjusted p-value). The results clearly support our interpretation, with $MAG510_{ov}$ and $MAGS2C_{ov}$ showing **strong (0.95 and 0.89, respectively) and statistically significant (adjusted p < 0.001) positive correlation with increasing seaweed inclusion level**. The correlation coefficient, raw p-value and FDR-adjusted p-value are now provided in Supplementary Table 4, and MAGs with p < 0.001 are marked in Figure 2B using asterisk. Relevant information has been included in Figure legend (Figure 2), and in Material and Methods. All underlying data are available in Supplementary Data 1, and the full statistical analysis script has been deposited in Zenodo, under an updated DOI (doi: 10.5281/zenodo.14515212).
